# Reduction of CO_2_ by a masked two-coordinate cobalt(i) complex and characterization of a proposed oxodicobalt(ii) intermediate†‡

**DOI:** 10.1039/c8sc02599a

**Published:** 2018-11-09

**Authors:** Lisa Roy, Malik H. Al-Afyouni, Daniel E. DeRosha, Bhaskar Mondal, Ida M. DiMucci, Kyle M. Lancaster, Jason Shearer, Eckhard Bill, William W. Brennessel, Frank Neese, Shengfa Ye, Patrick L. Holland

**Affiliations:** a Max Planck Institute for Chemical Energy Conversion Stiftstraße 34-36 Mülheim an der Ruhr D-45470 Germany; b Department of Chemistry, University of Rochester Rochester New York 14618 USA; c Department of Chemistry, Yale University New Haven Connecticut 06520 USA patrick.holland@yale.edu; d Department of Chemistry and Chemical Biology, Baker Laboratory, Cornell University Ithaca New York 14853 USA; e Department of Chemistry, Trinity University San Antonio Texas 78212 USA; f Max Planck Institute for Coal Research Kaiser-Wilhelm-Platz 1 Mülheim an der Ruhr D-45470 Germany shengfa.ye@kofo.mpg.de; g CSIR Central Mechanical Engineering Research Institute Durgapur 713209 India

## Abstract

Fixation and chemical reduction of CO_2_ are important for utilization of this abundant resource, and understanding the detailed mechanism of C–O cleavage is needed for rational development of CO_2_ reduction methods. Here, we describe a detailed analysis of the mechanism of the reaction of a masked two-coordinate cobalt(i) complex, L^*t*Bu^Co (where L^*t*Bu^ = 2,2,6,6-tetramethyl-3,5-bis[(2,6-diisopropylphenyl)imino]hept-4-yl), with CO_2_, which yields two products of C–O cleavage, the cobalt(i) monocarbonyl complex L^*t*Bu^Co(CO) and the dicobalt(ii) carbonate complex (L^*t*Bu^Co)_2_(μ-CO_3_). Kinetic studies and computations show that the κ*N*,η^6^-arene isomer of L^*t*Bu^Co rearranges to the κ_2_*N*,*N′* binding mode prior to binding of CO_2_, which contrasts with the mechanism of binding of other substrates to L^*t*Bu^Co. Density functional theory (DFT) studies show that the only low-energy pathways for cleavage of CO_2_ proceed through bimetallic mechanisms, and DFT and highly correlated domain-based local pair natural orbital coupled cluster (DLPNO-CCSD(T)) calculations reveal the cooperative effects of the two metal centers during facile C–O bond rupture. A plausible intermediate in the reaction of CO_2_ with L^*t*Bu^Co is the oxodicobalt(ii) complex L^*t*Bu^CoOCoL^*t*Bu^, which has been independently synthesized through the reaction of L^*t*Bu^Co with N_2_O. The rapid reaction of L^*t*Bu^CoOCoL^*t*Bu^ with CO_2_ to form the carbonate product indicates that the oxo species is kinetically competent to be an intermediate during CO_2_ cleavage by L^*t*Bu^Co. L^*t*Bu^CoOCoL^*t*Bu^ is a novel example of a thoroughly characterized molecular cobalt–oxo complex where the cobalt ions are clearly in the +2 oxidation state. Its nucleophilic reactivity is a consequence of high charge localization on the μ-oxo ligand between two antiferromagnetically coupled high-spin cobalt(ii) centers, as characterized by DFT and multireference complete active space self-consistent field (CASSCF) calculations.

## Abstract 2

The detailed mechanism of CO_2_ reduction by a cobalt(i) complex is investigated using computations and kinetics.

## Graphical abstract

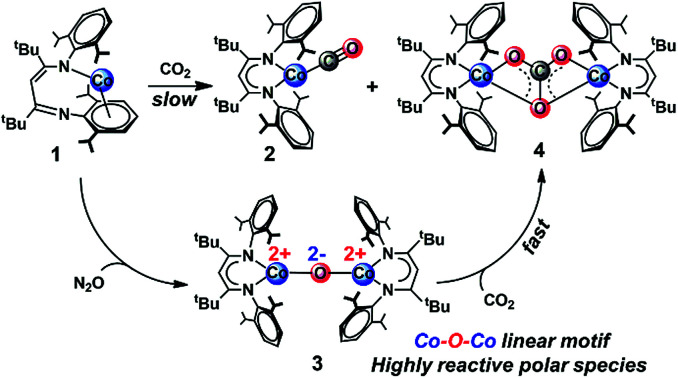

## Introduction

Terminal oxo complexes of transition metals are often invoked as intermediates in hydrocarbon activation,^1–3^ oxygen atom transfer^4,5^ and water oxidation.^6,7^ Dinuclear oxo-bridged systems have garnered less attention, even though they have numerous important roles in reactive metallocofactors,^8,9^ materials,^10^ catalysts,^11^ and physiological processes.^12^ Like other first row transition metals, cobalt has received growing interest for catalysis due to its versatility and low cost.^13^ In particular, cobalt species containing doubly bridged oxo- or hydroxo-bridged cobalt subunits^14^ are in focus in the context of water oxidation.^15,16^ However, dinuclear Co–O–Co compounds with a single oxo bridge, the simplest bridging cobalt oxo species, are rare and only two examples have been reported. Stauber *et al.* presented a formally dianionic dicobalt(iii) oxo species, for which XAS and computational results indicated an unusual electronic structure comprising two cobalt(ii) ions, a bridging oxyl radical and an additional “hole” on the supporting ligand.^17^ The earlier example of a dicobalt oxo species by Zhang *et al.* does not include sufficient spectroscopic details to establish the oxidation states of the metals.^18^ Thus, understanding the chemical behavior of dicobalt(ii) oxo complexes remains elusive.

Here, we describe a new oxodicobalt(ii) complex that arises in the context of CO_2_ reduction. Carbon dioxide is a persistent environmental pollutant and a C1 feedstock for chemical industries, which has inspired a large amount of research on transition metal catalysts for CO_2_ reduction.^19–21^ The practical motivations for CO_2_ transformation are accompanied by fundamental interest in the detailed mechanisms and charge localization in reduced CO_2_ intermediates.^19,22^ Here, we focus on cobalt complexes, which are under active study because they are adept at catalytic reductions of CO_2_ to CO.^13,23–31^

In the work described here, we use the reduced, unsaturated cobalt site in L^*t*Bu^Co (1), a “masked” two-coordinate complex supported by the β-diketiminate ligand 2,2,6-6-tetramethyl-3,5-bis[(2,6-diisopropylphenyl)imino]hept-4-yl (L^*t*Bu^).^32^ Its reaction with CO_2_ cleaves a C–O bond in CO_2_, and we use a combination of experimental kinetics, density functional theory (DFT), and highly correlated domain-based local pair natural orbital coupled cluster theory with single, double, and perturbative triple excitations (DLPNO-CCSD(T)) to show cooperation of two Co ions for facilitating a bimetallic reaction pathway for CO_2_ reduction. We demonstrate that a likely intermediate is the Co^2+^–O^2−^–Co^2+^ complex L^*t*Bu^CoOCoL^*t*Bu^, which can be independently synthesized using N_2_O. We investigate the electronic structure and reactivity of this new oxodicobalt(ii) complex in detail, including multi-reference complete active space self-consistent field (CASSCF) calculations.

## Results and discussion

### Activation of CO_2_ by L^*t*Bu^Co

**Scheme 1:**
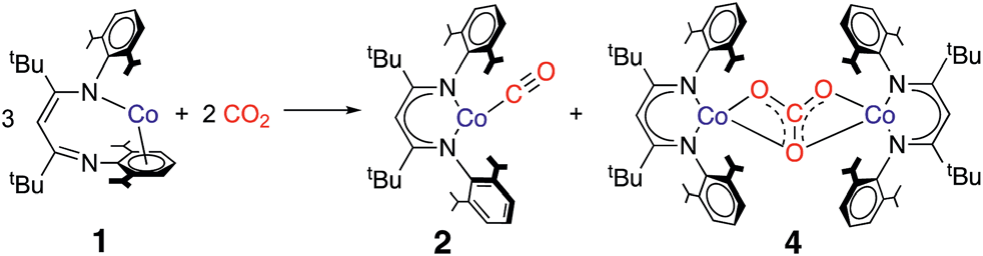
Reaction of L^*t*Bu^Co (1) with CO_2_ to form 2 and 4.

The cobalt(i) source L^*t*Bu^Co (1) was synthesized and characterized earlier,^32^ and features a supporting β-diketiminate ligand that is bound in an unusual k^1^,η^6^ binding mode as shown at the left of Scheme 1. The reactivity of 1 includes the binding of Lewis bases like THF, CO, N_2_, pyridine and PPh_3_, and the cleavage of C–F bonds in fluoroarenes.^32,33^ We also reported the activation of O_2_ at 1.^34^ Each of these reactions causes ligand rearrangement to the more typical κ^2^-binding mode of the β-diketiminate ligand in the three-coordinate or four-coordinate products. This led to a description of 1 as a “masked” low-valent two-coordinate Co complex. However, in these studies, a two-coordinate isomer of 1 with κ^2^-binding of diketiminate was never formed; kinetic studies of the reaction of 1 with pyridine showed a first-order dependence on pyridine coordination which, when combined with supporting DFT investigations, indicated that the incoming ligand coordinates *prior* to “slipping” of the arene.^32^ Computational studies on the reaction with CO indicated a similar associative pathway, in which ligand binding precedes diketiminate rearrangement.^35^ The high reactivity of 1 toward small molecules, in conjunction with our interest in understanding the role of low-valent cobalt complexes in CO_2_ reduction, prompted us to investigate its reaction with CO_2_. We were limited to aliphatic hydrocarbon solvents, due to the reaction of 1 with arenes and ethers.^32^

The addition of 10 molar equivalents of CO_2_ gas to L^*t*Bu^Co in cyclohexane-*d*_12_ causes a slow color change from brown to red-orange over several hours at 10 °C. ^1^H NMR spectroscopy shows the appearance of new paramagnetically shifted peaks (Fig. S3‡). Comparison of the resultant spectra to those of independently synthesized compounds shows that the products are a 1 : 1 mixture of previously reported L^*t*Bu^Co(CO) (2)^35^ and the new carbonate-bridged compound L^*t*Bu^Co(μ-OCO_2_)CoL^*t*Bu^ (4) (Scheme 1). Comparison to an internal integration standard indicates that 2 and 4 are each formed in >86% spectroscopic yield. These two products can be separated and isolated as pure solids in 66% and 40% yields, respectively, with the lower isolated yields attributed to losses during crystallization.

### Characterization of the dicobalt(ii) carbonate product

**Fig. 1:**
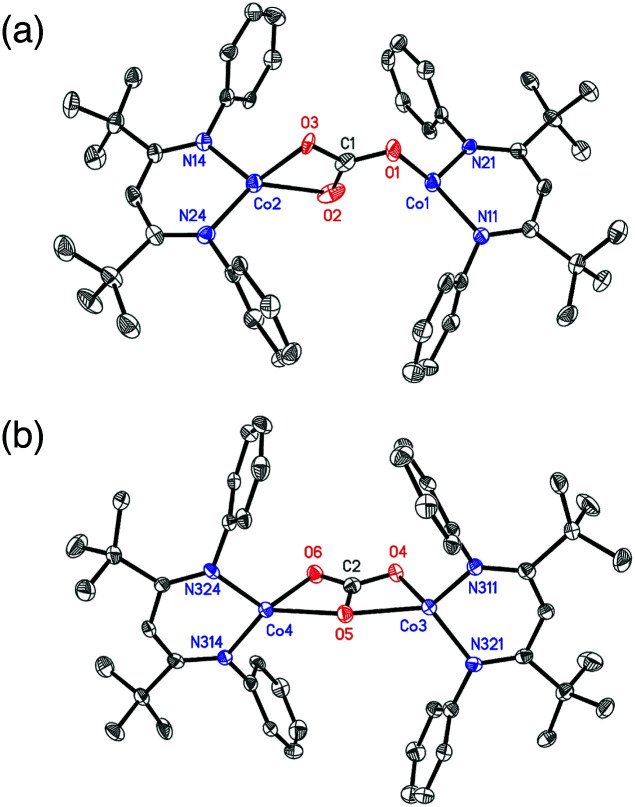
The two crystallographically independent molecules in the crystal structure of 4: (a) μ-η^1^:η^2^ form, with the major disorder component shown; (b) μ-η^2^:η^2^. H atoms and ^i^Pr groups are omitted for clarity. Thermal ellipsoids are shown at 50% probability.

X-ray quality crystals of 4 were grown from toluene, and analysis of the diffraction data revealed two independent sites in the asymmetric unit with different carbonate binding modes to the Co centers: (site a) μ-η^1^:η^2^ and (site b) μ-η^2^:η^2^ (Fig. 1). Site a also had disorders in the core, with two conformations of μ-η^1^:η^2^ carbonate in a 77 : 23 ratio that differ by which Co atom is η^1^ and which is η^2^. The major component in site a is discussed here for simplicity. Two binding modes of a bridging carbonate were also observed in the crystal structure of an analogous Fe complex.^36^ Most M–O distances are shorter in the Co compound, which is attributable to its smaller ionic radius. However, the Co(1)–O(1) bond in the η^1^:η^2^ molecule (1.921(4) Å) (see Table 1) is longer than the analogous Fe–O bond (1.881(1) Å),^36^ suggesting that π-bonding in the η^1^–Fe–O interaction is greater than that for the Co–O interaction. Complex 4 has averaged *D*_2h_ symmetry in solution, as ascertained by the presence of seven signals in the ^1^H NMR spectrum, suggesting that the carbonate interconverts rapidly between the η^1^:η^2^ and η^2^:η^2^ binding modes in solution.

**Table 1:** Selected interatomic distances (Å) and angles (°) in the η^1^:η^2^ and η^2^:η^2^ forms of 4 and comparison to the calculated structure^a^

	η^1^:η^2^	η^2^:η^2^	
		Expt.	Calc.
Co(1)–O(1)/Co(3)–O(4)	1.921(4)	1.970(3)	2.01
Co(1)–O(2)/Co(3)–O(5)	2.720(8)	2.218(3)	2.23
Co(2)–O(2)/Co(4)–O(5)	2.138(5)	2.211(3)	2.25
Co(2)–O(3)/Co(4)–O(6)	1.985(3)	1.979(2)	2.00
C(1)–O(1)/C(2)–O(4)	1.276(8)	1.321(4)	1.28
C(1)–O(2)/C(2)–O(5)	1.290(7)	1.266(4)	1.32
C(1)–O(3)/C(2)–O(6)	1.294(7)	1.263(4)	1.28
O(1)–C(1)–O(2)/O(4)–C(2)–O(5)	121.8(6)	117.4(3)	117.60
O(2)–C(1)–O(3)/O(5)–C(2)–O(6)	116.1(6)	117.4(3)	117.61
O(3)–C(1)–O(1)/O(4)–C(2)–O(6)	122.1(6)	125.1(3)	124.79

a The optimized geometry with *S* = 3. The BP86/B1 (B1 = TZVP basis set on Co, O, N and carbonate C, and def2-SVP on the rest of the atoms) level of theory was used to model the η^2^:η^2^ conformer. See the ESI for computational details.

### Experimental characterization of a cobalt(ii) oxo complex

**Scheme 2:**
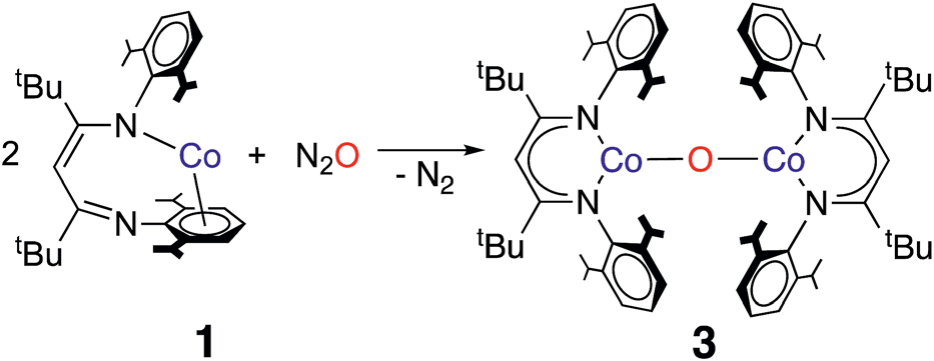
Synthesis of 3 from L^*t*Bu^Co and nitrous oxide.

**Fig. 2:**
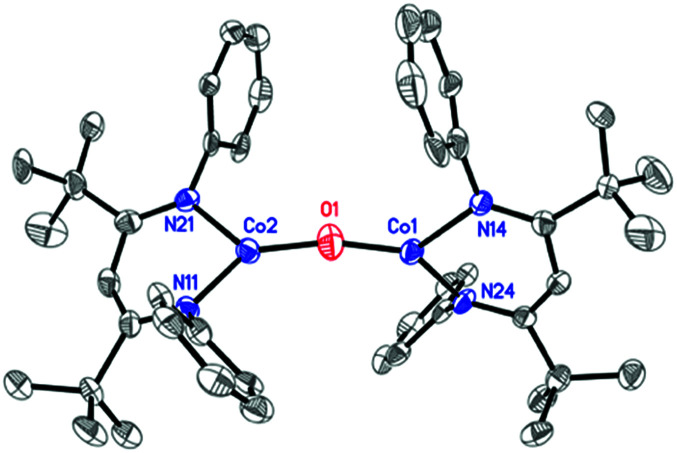
X-ray crystal structure of 3. Thermal ellipsoids are shown at 50% probability. H atoms and isopropyl groups are omitted for clarity.

The formation of a carbonate bridge in 4 implicates an unobserved oxocobalt species as an intermediate (see the following sections for calculations). In fact, in an earlier study on CO_2_ reduction by L^*t*Bu^FeNNFeL^*t*Bu^, Sadique *et al.* proposed that the formation of a carbonate bridge proceeds through an oxodiiron(ii) intermediate.^37^ Despite a number of attempts made using ^1^H NMR and UV-vis spectroscopy at temperatures between −80 °C and 25 °C, we observed no intermediates during the formation of 4 from L^*t*Bu^Co and CO_2_. Therefore, we chose to experimentally test the accessibility of an oxodicobalt(ii) species by synthesizing it through a different route. We added 1 equiv. of N_2_O to a solution of 1 in hexane at room temperature for 4 h, and after workup, red-orange 3 was isolated in 51% yield (Scheme 2). The solid-state structure (Fig. 2 and Table 2) shows 3 to be a dinuclear cobalt complex bridged by a single oxygen atom. The Co–O distance of 1.704(4) Å is much shorter than that in the only other fully characterized oxo-bridged dicobalt complex, which is four coordinated at each cobalt atom and has a Co–O bond distance of 1.995(11) Å.^17^ The Co–O bond in 3 is shorter than the distance of 1.784(3) Å found in ([Me_2_NN]Co)_2_(μ-O)_2_ ([Me_2_NN] = 2,4-bis[(2,6-dimethylphenyl)imino]pent-3-yl), a bis(μ-oxo)dicobalt(iii) complex reported by Dai *et al.*^38^ The Co–O–Co in 3 is slightly bent at 166.9(3)°. The Co–N bond distances are also shorter (<1.96 Å) than those in four-coordinate diketiminatocobalt(ii) complexes^32^ and agree well with those observed for other three-coordinate cobalt(ii) complexes.^39^ The C–C and C–N bond distances within the β-diketiminate of 3 are within the error of the analogous distances in the known three-coordinate cobalt(ii) compound L^*t*Bu^CoCl,^40^ suggesting that there is no change in the oxidation state of the supporting ligand (see ESI Section O‡). The redox innocence importantly implies a physical oxidation state of +2 for both metal centers.

**Table 2:** Selected interatomic distances (Å) and angles (°) in 3 and comparison to the calculated structure^a^

	Expt.	Calc.
Co1–O1	1.704(4)	1.72
Co1–N24	1.913(4)	1.89
Co1–N14	1.950(4)	1.91
Co2–O1	1.699(4)	1.72
Co2–N11	1.919(4)	1.89
Co2–N21	1.949(4)	1.91
Co1–O1–Co2	166.9(3)	165.5
O1–Co1–N24	136.7(2)	134.5
O1–Co1–N14	124.2(2)	123.8
N24–Co1–N14	98.7(2)	101.7
O1–Co2–N11	135.2(2)	135.5
O1–Co2–N21	125.3(2)	122.9
N11–Co2–N21	99.4(2)	101.6
N24–N14–N21–N11	85.8	82.3

a The optimized geometry of the *S* = 0 ground state (from antiferromagnetic coupling of cobalt site spins) using the BP86/B1 (B1 = TZVP basis set on Co, O, and N; def2-SVP on the rest of the atoms) level of theory. See the ESI for computational details.

In order to further test the metal oxidation state, we collected cobalt K-edge X-ray absorption spectroscopy (XAS) data for 3 and several previously reported compounds: three-coordinate cobalt(ii) complex L^*t*Bu^CoCl,^39^ four-coordinate oxygen-coordinated cobalt(ii) complex L^*t*Bu^Co(μ-OH)_2_CoL^*t*Bu^, and four-coordinate oxygen-coordinated cobalt(iii) complex L^*t*Bu^Co(μ-O)_2_CoL^*t*Bu^.^34^ The pre-edge and edge features overlapped in all of the compounds, including the previously reported cobalt(ii) and cobalt(iii) analogues (Fig. S10‡), indicating that XAS does not unambiguously distinguish the oxidation level. This ambiguity is unfortunate, but fairly common.^41^ Comparison of cobalt(ii) and cobalt(iii) species using X-ray photoelectron spectroscopy gave similarly ambiguous results.

The presence of an oxo in 3 is particularly notable, given the paucity of cobalt(ii) oxo complexes.^17,18^ Co^III^_2_(μ-O)_2_ complexes have also been described.^34,38^ Another relevant comparison is to the diiron(ii) complex [L^*t*Bu^Fe]_2_O,^42^ which has the same supporting ligand and connectivity as 3. The M–O bond length is shorter for cobalt (Co–O = 1.704(4) Å) than iron (Fe–O = 1.7503(4) Å), as expected from the smaller ionic radius of cobalt(ii) *versus* iron(ii). The method of preparing 3 is also interesting, because N_2_O is often kinetically inert, particularly in reactions with late transition metal complexes.^43–45^ This serves as another demonstration of the high reactivity of the masked two-coordinate complex L^*t*Bu^Co toward cleaving strong bonds.^32,34^

### Electronic structure of the Co–O–Co core and its connection to reactivity

Previous studies have highlighted the correlation between reactivity and the electronic structure.^46–49^ We first carried out broken symmetry density functional theory (DFT) calculations on 3 using the BP86 functional. Geometry optimization with BP86 and a mixed basis set combination, B1 (triple-ζ quality TZVP^50^ basis set on Co, O, N, and selected C atoms, and a double ζ quality split-valence basis set, def2-SVP,^51^ on the rest of the atoms, in a polarizable continuum solvent model, CPCM,^52^ using *ε* = 2.3 for benzene, and D3BJ empirical dispersion), gave a core geometry that is in good agreement with the X-ray crystal structure, including the slight Co–O–Co bending, as shown in Table 2. Using this geometry, single-point calculations with pure BP86, *meta*-GGA M06L, and hybrid B3LYP density functionals all predicted a local high-spin d^7^ configuration for each cobalt center. The two cobalt(ii) centers are antiferromagnetically coupled, which is achieved through three pathways as indicated by three spin-coupled pairs with overlaps in the range of 0.2 to 0.5. The metal–ligand interactions are largely ionic, because doubly occupied metal d-centered orbitals and the spin coupled orbital pairs have >90% Co d-parentage. A similar electronic structure is observed for the hypothetical high spin ferromagnetically coupled species. These analyses indicated the electronic structure of 3 to be a dicobalt(ii) oxo irrespective of the density functional employed (see ESI Section U‡).

**Fig. 3:**
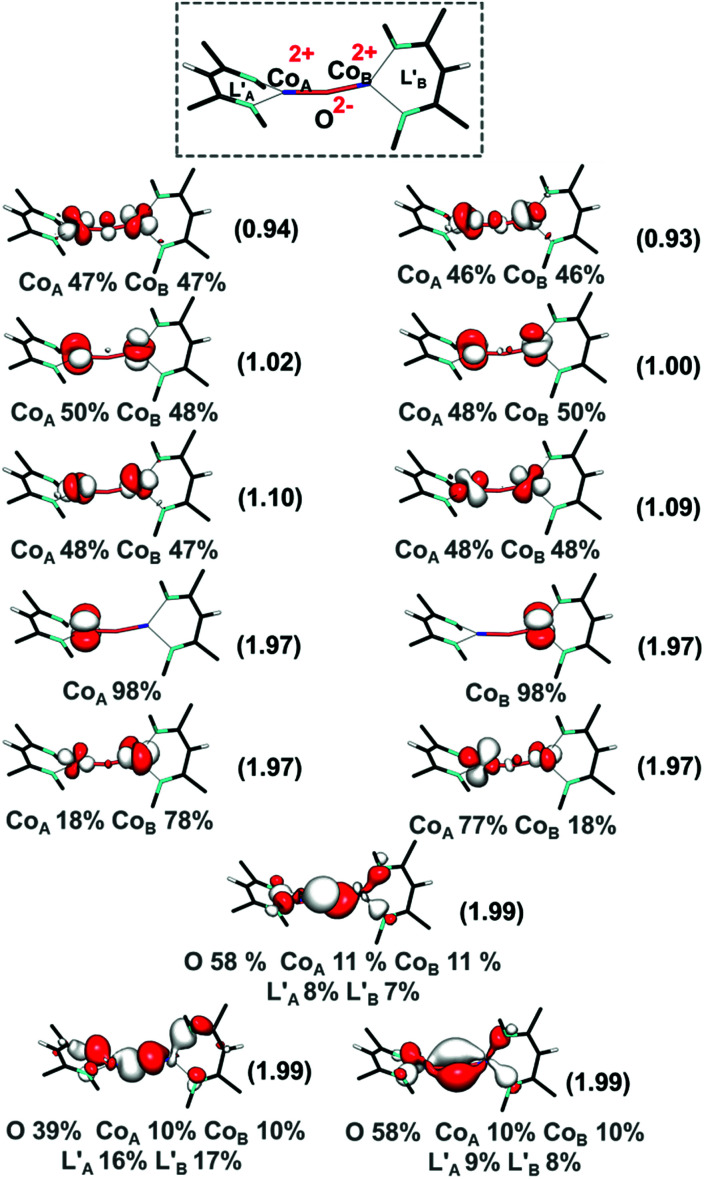
CASSCF(20,13) natural orbitals along with occupation numbers in parentheses for the *S* = 0 state of intermediate 3. Atomic contributions to each orbital are also shown.

The unusual electronic structure of another dicobalt oxo species^17^ encouraged us to examine the electronic structure of intermediate 3 more carefully. Therefore, we pursued CASSCF calculations (on the DFT-optimized geometry) using an active space that distributes 20 electrons into 13 orbitals (CASSCF(20,13)), the ten cobalt 3d-based orbitals and the three 2p orbitals of the oxo ligand. For the present purpose of analyzing metal–ligand bonding, it is not necessary to employ an enlarged active space including double d and/or p shells of the metal center and the oxo ligand. As shown in Fig. 3, the singlet wavefunction has strong multireference character, with several competing configuration state functions having a weight in the range of 0.2–6.2% (Table S5‡). Inspection of the natural orbitals obtained from the CASSCF(20,13) calculations shows that complex 3 is best described as having two high spin cobalt(ii) centers bridged by an oxo ligand, Co^II^–O^2−^–Co^II^. Notably, the electronic structure of 3 differs from that proposed for the previously reported Co–O–Co complex, which contains two cobalt(ii) ions, an oxyl ligand, and a “hole” in the supporting ligand.^17^ Note that due to the substantial multireference character, one cannot apply the CCSD approach to the singlet state. The septet CASSCF(20,13) solution is essentially single reference in nature, because the leading configuration accounts for 88% of the wavefunction. We further enlarged the active space to include diketiminate π and π* orbitals (CASSCF(24,17)), which accounts for the possibility of ligand-based radicals, as discussed above for another dicobalt oxo complex found in the literature.^17^ However, the larger CASSCF(24,17) active space predicts an identical bonding picture to CASSCF(20,13) (for details, see ESI, Section P‡). Therefore, we discuss only the CASSCF(20,13) solution.

In line with the DFT results, the Co–O interaction computed by CASSCF for 3 is very polar, with only ∼20% cobalt 3d character in the oxygen-based orbitals and predominant cobalt d-parentage (>90%) in the metal-based orbitals. This is quite different from high-valent mononuclear metal–oxo species like ferryls, which feature more covalent metal–ligand interactions.^53–56^ For example, in [Fe(O)(TMC)(NCCH_3_)]^2+^ (TMC = tetramethylcyclam), there is 56% Fe(d) and 32% O(p) character in the Fe–O σ-bond and 54% Fe(d) and 36% O(p) in the Fe–O π-bond.^53^ The different bonding picture in 3 can be attributed to the lower oxidation state of cobalt and the competitive bonding of the two metal centers with the oxo ligand. Furthermore, the calculated high electron density on the oxygen atom, as found from the population analysis (Mulliken gross atomic charges on O = −0.6195 a.u. and on Co centers = 0.6036 and 0.5989 a.u. at the BP86/def2-TZVPP level of theory; see ESI, Section U‡), is consistent with facile nucleophilic attack on CO_2_. The bonding picture in 3 contrasts with high-valent metal–oxo intermediates, in which covalent metal–oxo interactions govern the electrophilic reactivity.^46,57,58^

### Kinetic studies on the CO_2_ reduction pathway

Initial insight into the mechanism of CO_2_ reduction by L^*t*Bu^Co was gained through kinetic studies using ^1^H NMR spectroscopy. After injection of a solution of excess CO_2_ into a solution of 1 in C_6_D_12_ at 10 °C, the reaction was monitored by NMR spectroscopy (see ESI Section C for details‡). The concentrations of 1, 2 and 4 fit to exponential decays over more than six half-lives. The first-order rate constant of 3.7 ± 0.5 × 10^−4^ s^−1^ was independent of the flooding concentration of CO_2_, indicating that the rate law has the form rate = *k*[1]. The zero-order dependence of the rate on [CO_2_] indicates that the rate-limiting step occurs prior to CO_2_ binding and prevents the use of kinetic measurements to elucidate steps after CO_2_ binding. Hence, we use computations to evaluate these steps below.

**Scheme 3:**
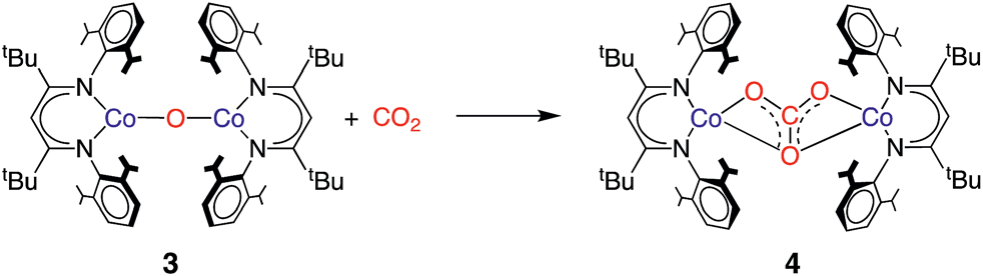
Reaction of CO_2_ with 3 to form 4.

We considered that 3 could be formed as an intermediate that could react with CO_2_ rapidly to give 4; if its consumption were more rapid than its formation, it might not be observed during the reaction. With pure samples of 3 in hand, we tested this hypothesis. Treating a solution of 3 in C_6_D_12_ with 1.5 equivalents of CO_2_ for 2 min at 10 °C resulted in a high conversion (75%) of 3 to 4 (Scheme 3 and Fig. S5‡). The much more rapid reaction of CO_2_ with 3 compared to 1, furnishing the same product, indicates that the reaction of the oxo species with CO_2_ is kinetically competent to be a step in the formation of 2 and 4 from 1 and CO_2_.

### Computational investigations on the mechanism of CO_2_ reduction

**Chart 1:**
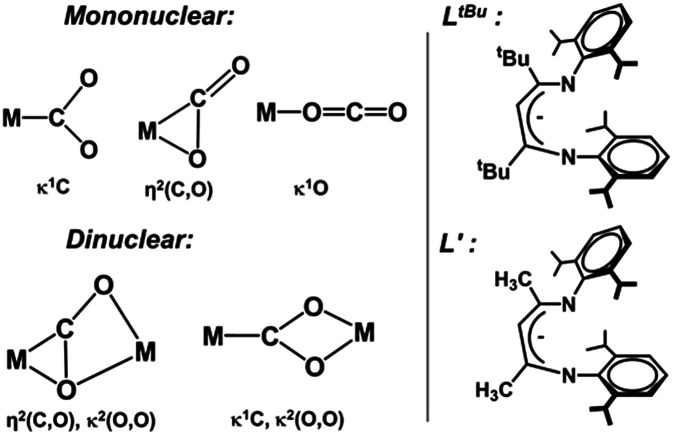
(Left) Different binding modes of CO_2_ to mononuclear and dinuclear metal sites. (Right) Actual and truncated ligand frameworks.

To gain additional insight into the reaction mechanism, we pursued computations using a model in which the bulky β-diketiminate ligand (L^*t*Bu^) was slightly truncated to L′ (2,4-bis[(2,6-diisopropylphenyl)imino]pent-3-yl, Chart 1, right) where *t*Bu substituents are replaced by methyl groups. The overlay of the crystal structure of 1 and the optimized geometry with the truncated ligand (Fig. S11 and Table S2‡) shows that truncation leads to negligible differences in key metrical parameters. As above, the BP86(CPCM,D3BJ)/B1 level of theory was employed to optimize geometries and compute frequencies. We chose the local coupled-cluster approach with the DLPNO-CCSD(T) method to verify the reliability of crucial stationary points obtained with the *meta*-GGA M06L and hybrid B3LYP methods. We note that the CCSD(T) approach cannot be applied to complexes involving two antiferromagnetically coupled metal centers, such as the open-shell singlet state of complex 3. Therefore, we performed the DLPNO-CCSD(T) calculations on the spin-aligned state of the bimetallic complexes. Although the CASSCF approach accounts for static correlation of electrons correctly (*i.e.* near-degeneracy effects), to produce reliable energies, it has to be followed by CASPT2 or NEVPT2 corrections to capture the dynamic correlation for instantaneous electron motions. Because of the exorbitant computational cost, this combined approach was not used to evaluate the energies of all intermediates and transition states. Instead, we employed open-shell DLPNO-CCSD(T), a straightforward approach, to construct reliable potential energy surfaces. For more details about the computational methods and basis sets used, see ESI, Section A.‡

#### Ligand isomerization and CO_2_ binding

We first focused on the ligand framework rearrangement and CO_2_ binding, for which different CO_2_ binding modes were considered as shown in Chart 1 (left). Our earlier kinetic studies demonstrated that coordination of pyridine to 1 induces isomerization of the β-diketiminate ligand from the κ*N*,η^6^-arene isomer to the traditional κ^2^*N*,*N′* form in less than 1 s at −40 °C, with a barrier of Δ*G*^‡^ = 10.1 kcal mol^−1^.^32^ The associative nature of this isomerization was supported by computational studies, which indicated that pyridine and CO each induce arene slipping.^35^ On the other hand, we find here that CO_2_*does not* provide analogous assistance in arene slipping. Therefore, in contrast to the binding of pyridine and CO, the arene slip/imine flip isomerization must occur prior to CO_2_ coordination, and this explains the slow rate of the reaction.

**Fig. 4:**
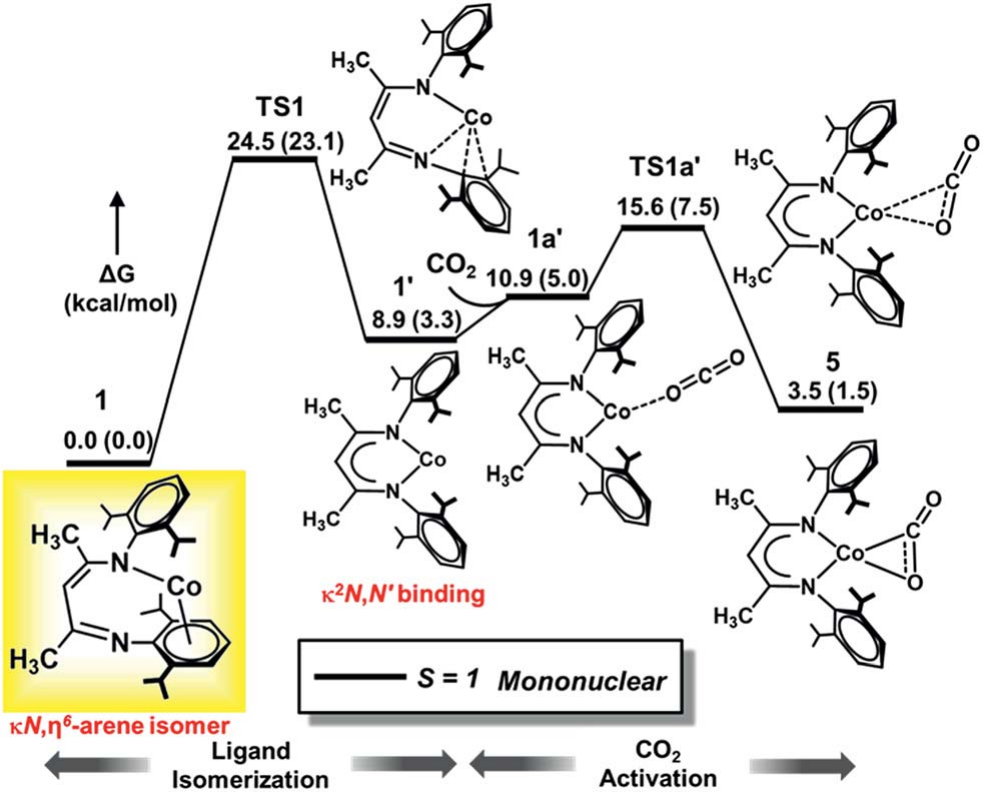
Computed DLPNO-CCSD(T)/def2-TZVPP Gibbs free energy (Δ*G*) profile for isomerization of 1 (L′Co) to 1*′* followed by CO_2_ activation. Relative free energy values (in kcal mol^−1^) computed with M06L/def2-TZVPP are given in parentheses. The isolated species is shown in the yellow box, with L′ depicted instead of L^*t*Bu^.

Compound 1 was previously termed a “masked two-coordinate” complex, and two-coordinate isomer 1*′* with κ^2^*N*,*N′* bonding (Fig. 4) is never observed experimentally. Our high-level computations with DLPNO-CCSD(T)/def2-TZVPP and M06L/def2-TZVPP show that two-coordinate isomer 1*′* with a κ^2^*N,N′* bonding mode lies at a higher energy than 1 (8.9 kcal mol^−1^ at DLPNO-CCSD(T)/def2-TZVPP and 3.3 kcal mol^−1^ at M06L/def2-TZVPP), consistent with the experimental results.

Furthermore, diketiminate isomerization *without* assistance is experimentally known to have a barrier of >15 kcal mol^−1^ because no isomerization between binding modes was observed in variable-temperature ^1^H NMR studies of L^*t*Bu^Co.^32^ Consistent with these experiments, our DLPNO-CCSD(T)/def2-TZVPP calculations predict a barrier of Δ*G*^‡^ = 24.5 kcal mol^−1^ for TS1 (Fig. 4), which agrees well with the experimental rate that gives Δ*G*^‡^ = 21.0 kcal mol^−1^ using the Eyring equation (see Section C in the ESI‡). The M06L functional with the def2-TZVPP basis set also delivers a similar value of 23.1 kcal mol^−1^, whereas B3LYP-D3BJ considerably underestimates the barrier with Δ*G*^‡^ = 13.6 kcal mol^−1^ (Table 3). It is notable that the B3LYP-D3BJ/def2-TZVPP calculations also erroneously predicted triplet κ^2^*N*,*N′* isomer 1′ to be much lower in energy than κ*N*,η^6^-arene isomer 1 by 10 kcal mol^−1^, in disagreement with the experimental data. Hence, for this specific case, the B3LYP-D3BJ method does not give results consistent with those of the experiment. Please note that our earlier calculations on CO_2_ hydrogenation^59,60^ demonstrate that even with the D3BJ corrections, the B3LYP functional sometimes cannot appropriately describe non-covalent interactions (in the present case, the metal aryl π-bonding), whereas M06L is designed to account for such weak interactions (see Section H in the ESI‡).^61^ Hence, in the following sections we discuss the more reliable M06L energies and verify them on-the-fly by the DLPNO-CCSD(T) method and summarize the results obtained with other functionals in the ESI (Sections K and L‡).

**Table 3:** Selected barriers at different levels of theory, employing the def2-TZVPP basis set and CPCM(benzene) solvent model

	B3LYP	M06L	DLPNO-CCSD(T)
TS1^a^	13.6	23.1	24.5
TS2^b^	30.4	27.2	26.7
TS4^c^	21.6	20.5	20.3
TS7^c^	16.0	15.7	19.4

a Relative to 1.

b Relative to 5.

c Relative to 10.

To summarize, the N1/C^Ar^ movement of the metal center (1 → 1*′*) represents the rate-limiting transition state of the reaction with CO_2_ (TS1, Fig. 4), with a calculated barrier of about 24 kcal mol^−1^. The magnitude of this barrier is in good agreement with the experimentally determined barrier of 21.0 kcal mol^−1^ that is calculated from the first-order rate constant in the kinetic studies presented above (see ESI part C‡), and the identification of the rate-determining transition state prior to CO_2_ binding is also consistent with the independence of the rate on [CO_2_].

The closed-shell singlet κ*N*,η^6^-arene isomer of L′Co (1) lies 10 kcal mol^−1^ higher in energy than the triplet congener at the DLPNO-CCSD(T)/def2-TZVPP level of theory (Δ*G* ∼ 12 kcal mol^−1^ predicted with M06L/def2-TZVPP). Again, the M06L functional provides similar results to the DLPNO-CCSD(T) method and our calculations show that ligand isomerization on the closed shell singlet surface has a significantly high barrier (Δ*G*^‡^ = 49.6 kcal mol^−1^ using DLPNO-CCSD(T)/def2-TZVPP; Δ*G*^‡^ = 46.7 kcal mol^−1^ using M06L/def2-TZVPP). Therefore, the singlet surface is excluded from further consideration.

**Fig. 5:**
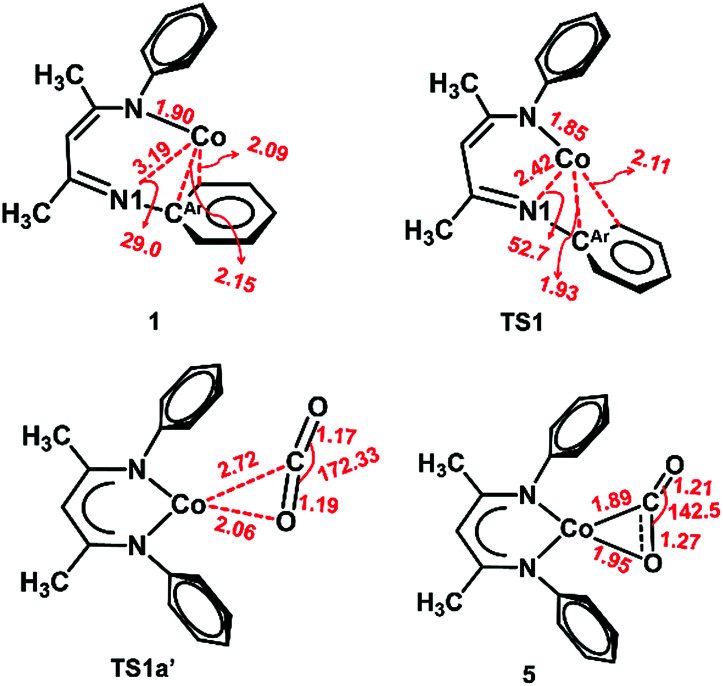
Optimized structures of 1, TS1, TS1a*′* and 5 at the BP86/B1 level of theory. Important interatomic distances (Å) and angles (°) are shown. Isopropyl groups are omitted for clarity.

The slippage of the β-diketiminate ligand furnishes an open site for CO_2_ binding. In contrast to the high endergonicity found for the direct CO_2_ coordination to 1, κ^1^O-bound CO_2_-adduct 1a*′* is only 2 kcal mol^−1^ higher in energy than 1*′*. The κ^1^-O binding mode in 1a*′* has been observed in the uranium complex ((^Ad^ArO)_3_tacn)U(CO_2_) where the CO_2_ ligand accepts an electron from the uranium center to produce a U^IV^–OCO^−^ complex.^62^ As depicted in Fig. 4, conversion of 1a*′* to a more stable η^2^-CO_2_ isomer (5) can easily occur by traversing a moderate barrier of 15 kcal mol^−1^. The two C–O bonds in 5 lengthen relative to those in 1a*′*, and the CO_2_ moiety becomes bent with an O–C–O angle of 142.5° (Fig. 5), indicating a substantial shift of electron density from Co to the CO_2_ π* orbital.^59^ Intermediate 5 is related to PP^Me^PNi(η^2^-CO_2_) (PP^Me^P = PMe[2-P^i^Pr_2_-C_6_H_4_]_2_) and (dtbpe)Ni(η^2^-CO_2_) (dtpbe = 1,2-bis(di-*tert*-butylphosphino)ethane), which also feature η^2^(C,O) coordination.^63,64^ Earlier computational investigations have suggested that CO_2_ coordination to metal β-diketiminate fragments (M = Cr, Ni) favors the η^2^(C,O) binding mode, similar to intermediate 5.^65^

#### Mononuclear dissociative pathway

**Fig. 6:**
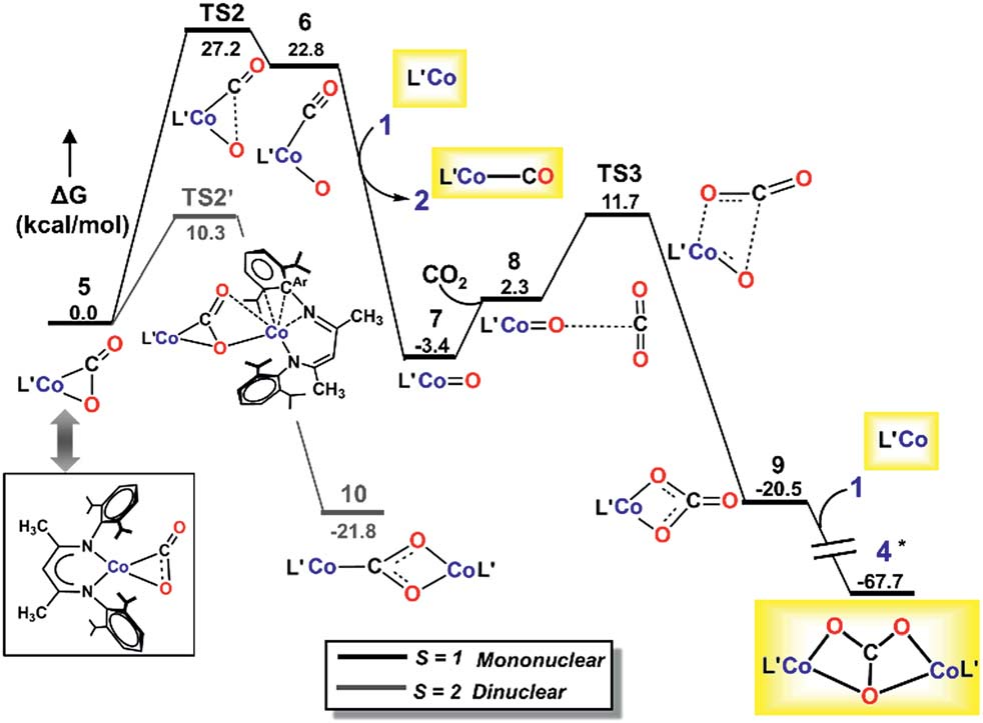
M06L/def2-TZVPP Gibbs free energy (Δ*G*) profile for the mononuclear dissociative pathway. Isolated species are shown in yellow boxes, with L′ depicted instead of L^*t*Bu^. *Marked species has lower relative free energy on the *S* = 3 surface which has been taken to complete the profile.

Two possible pathways may be envisioned for CO_2_ reduction by 1: associative and dissociative. The associative mechanism involves formation of a CO_2_ adduct followed by association of a second molecule of CO_2_*prior to* C–O bond cleavage/rearrangement to generate CO and carbonate. In contrast, adduct formation in the dissociative mechanism is followed by breaking a C–O bond to form CO and oxo, both of which are easily converted to products. We start by considering the mononuclear dissociative pathway (Fig. 6).

Due to the abundance of CO_2_ in solution, an additional CO_2_ molecule can be envisioned to add to intermediate **5** to produce a metal-bound carboxylate dimer. However, our repeated attempts to locate an intermediate resulting from association of a second CO_2_ molecule to 5 failed (see ESI, Section S‡). The lack of a mononuclear associative pathway emphasizes the need for bi-functional activation to engender sufficient nucleophilicity to bound CO_2_.^48^ The rather low nucleophilicity of the bound CO_2_ in 5 is not conducive to electron transfer to another incoming CO_2_. Moreover, the lack of a neighboring Lewis acid, which might help in bending of the incoming CO_2_ prior to activation, also inhibits facile association of a second CO_2_ molecule to the mononuclear adduct **5,** and hence a mononuclear associative pathway is not possible (see ESI, Section S‡).

As depicted in Fig. 6, our DLPNO-CCSD(T) calculations predict that the conversion of 5 to L′Co(CO)(O) (6, see ESI Section M for geometry‡) involves a high free-energy barrier in the mononuclear dissociative pathway (TS2, Table 3). Similar barriers were obtained using DLPNO-CCSD(T) (26.7 kcal mol^−1^), M06L (27.2 kcal mol^−1^) and B3LYP (30.4 kcal mol^−1^) calculations, and the latter value is in reasonable agreement with that (35.1 kcal mol^−1^) calculated in earlier work on a truncated cobalt(i) complex with no substituents on the β-diketiminate (1,3-bis-imino-prop-2-yl).^66^ Once formed, complex 6 easily transfers CO to 1*′*, leading to the mononuclear carbonyl complex (2) and a terminal oxo-cobalt species (7, Fig. 6). Complex 7 may initiate nucleophilic attack of the metal-coordinated oxo on incoming CO_2_ to form a mono-cobalt carbonate species (9) *via* a four-membered transition state (TS3, Δ*G*^‡^ = 9.4 kcal mol^−1^, Fig. 6). One may envision formation of the bridging oxo 3 from 6 or 7 in the presence of 1. The transformations were computed to have favorable driving forces; however, in the mononuclear dissociative pathway, the generation of **6** entails a high barrier for the C–O bond cleavage. Hence, these two pathways were not considered further (for details, see Section S in the ESI‡). Additionally, in the mononuclear pathway, the high barrier found for the C–O bond cleavage would lead to a buildup of 5, in contrast to the observed reaction course in which no intermediates are evident. This inconsistency with experimental evidence thus indicates that the mononuclear pathway is not feasible. We therefore explored the feasibility of the dinuclear dissociative and associative pathways, which are discussed in the following section.

#### Dinuclear pathways

**Fig. 7:**
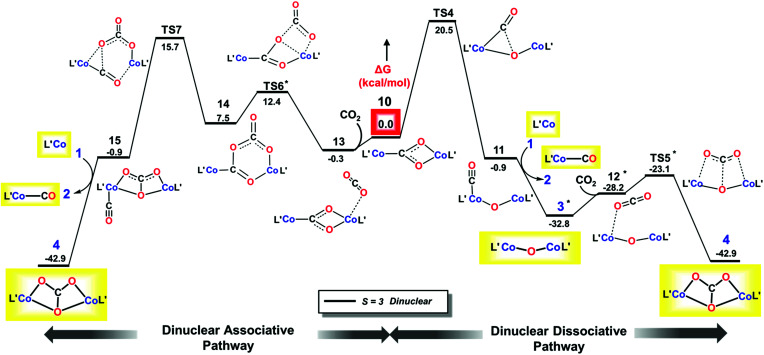
M06L/def2-TZVPP Gibbs free energy (Δ*G*) profile for dinuclear dissociative (right) and associative (left) pathways. Isolated species are shown in yellow boxes, with L′ depicted instead of L^*t*Bu^. *Marked species has lower relative free energy on the *S* = 0 surface which has been taken to complete the profile.

We considered a carboxylate bridged bimetallic species that could lead to reductive disproportionation. As shown in Fig. 6, reaction of L′CoCO_2_ (5) and L′Co (1) to form the dinuclear complex L′Co(CO_2_)CoL′ (10) is strongly exergonic by 21.8 kcal mol^−1^ and has a relatively low activation barrier of 10.3 kcal mol^−1^ (TS2*′*, Fig. 6) on the quintet surface. Isomerization of the diketiminate ligand in the incoming L′Co is assisted by coordination of the bound CO_2_ to the incoming metal, as described previously for pyridine or CO coordination; thus, the partially reduced CO_2_ moiety in 5 is a stronger Lewis base than free CO_2_. In line with this reasoning, the entire process of CO_2_ association with 1 to give 5 is energetically uphill, whereas the addition of 5 to the second molecule of 1 is downhill by more than 20 kcal mol^−1^. This is a key aspect of *bimetallic cooperation* that facilitates CO_2_ activation in this system. For complex 10, we found eight possible isomers, each containing a κ^1^C:κ^2^-O,O carboxylate bridge (CO_2_^2−^) (Chart 1, left), but differing in the local spin states of the two cobalt(ii) centers and exchange coupling (for details see Section J in the ESI‡). The two magneto-structural isomers that are lowest in energy have high spin cobalt(ii) centers with ferromagnetic (*S*_total_ = 3) or antiferromagnetic (*S*_total_ = 0) coupling, and these are nearly isoenergetic because of weak exchange coupling. The *S*_total_ = 2 species (derived from ferromagnetically coupled high spin and low spin cobalt(ii) centers) is only 3 kcal mol^−1^ higher in energy; thus, spin crossover from the quintet to the septet state is feasible (see Section L in the ESI‡). We assume that the efficient spin orbit coupling of the metal centers is sufficient to minimize any spin-crossover barriers, for which only the local spin state of the cobalt center changes. Starting from complex 10, we tested dissociative and associative mechanisms. In the dissociative route (toward the right of Fig. 7), 10 first undergoes C–O bond dissociation to generate CO and a μ-oxo species, and then addition of another CO_2_ molecule to the latter intermediate leads to formation of the carbonate-bridged bimetallic product. This reaction channel has been proposed for reductive cleavage of CO_2_ by L^*t*Bu^Fe–N_2_–FeL^*t*Bu^ and low-valent U(iii) complexes with the intermediacy of an M–O–M species.^62,67^ In the associative route (toward the left of Fig. 7), another CO_2_ molecule first inserts into 10 yielding a (CO_2_)_2_ linker, which then rearranges to CO and CO_3_^2−^. The associative mechanism was described for CO_2_ functionalization using [Re(dmb)(CO)_3_] (where dmb = 4,4′-dimethyl-2,2′-bipyridine) by Agarwal *et al.*^68^

**Fig. 8:**
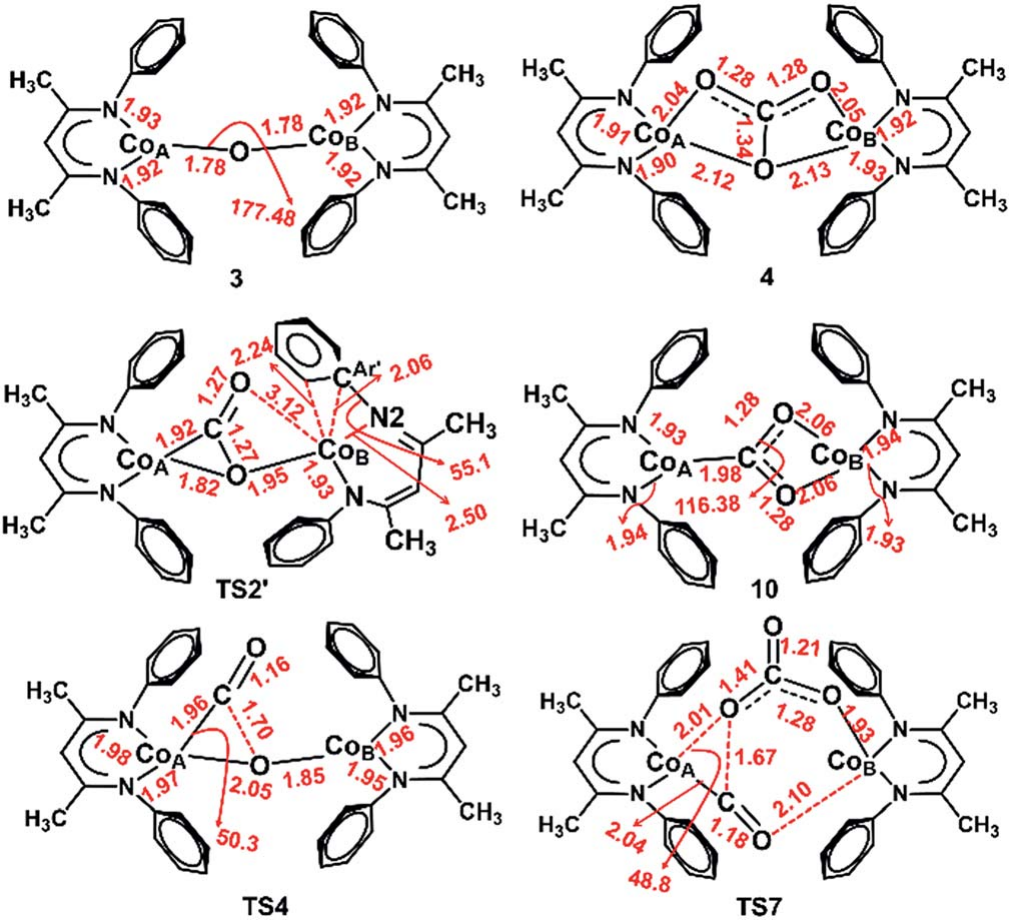
Optimized structures of 3, 4 and TS2*′* at the BP86/B1 level of theory and 10, TS4 and TS7 at the B3LYP/B1 level of theory. (See computational details for basis set information and justification of employing the respective methods.) Important interatomic distances (Å) and angles (°) are also shown. Isopropyl groups are omitted for clarity.

In the dissociative pathway, C–O bond cleavage in 10 passes through TS4 (see the geometry in Fig. 8) to afford an intermediate, L′Co(CO)–O–CoL′ (11), where two cobalt(ii) metal centers are bridged by an oxo group, and a terminal carbonyl ligand is bound to one cobalt(ii) center. The transformation is thermoneutral (Δ*G* = −0.9 kcal mol^−1^ relative to 10) and involves a moderate barrier of 20.3 kcal mol^−1^ at the coupled-cluster level of theory (DLPNO-CCSD(T)/def2-TZVPP, Table 3) on the *S* = 3 surface while M06L/def2-TZVPP also predicts a similar result (Δ*G*^‡^ = 20.5 kcal mol^−1^). Furthermore, M06L calculations using the broken symmetry formalism^69,70^ also furnish a similar barrier (21.9 kcal mol^−1^) on the singlet surface (Fig. S17‡). We note that attempts to independently synthesize 11 by addition of 1 eq or 1 atm CO to the oxo 3 instead furnished the previously reported complex L^*t*Bu^Co(CO)_2_ (see ESI Section D‡).^35^

As depicted in Section L in the ESI,‡ further conversion of complex 11 to final product 4 has similar reaction energies and barriers for the different spin coupling situations. Therefore, we focus on the septet surface only (computed using M06L(CPCM)/def2-TZVPP) and summarize the results of the singlet state in Section L in the ESI.‡ CO transfer from 11 to 1 to form the three-coordinate L′Co(CO) product (2) and an oxodicobalt(ii) species (3) is computed to be highly thermodynamically favored with a reaction free energy of *ca.* −32 kcal mol^−1^. The addition of CO_2_ to the oxo ligand in 3 can easily occur with a low barrier of 5.8 kcal mol^−1^, in agreement with the observation (see above) that L^*t*Bu^CoOCoL^*t*Bu^ reacts rapidly with CO_2_ at room temperature to yield 4.

For the associative mechanism, our calculations show that nucleophilic addition of CO_2_ to the electron-rich oxygen atom of the bridging carboxylate dianion in 10 can form a C(O)–O–C(O)–O^2*−*^ bridge between the two cobalt(ii) centers in 14*via* a four-membered transition state (TS6) that lies 13.5 kcal mol^−1^ above 10. This is followed by rupture of a C–O bond in the original CO_2_ through TS7 (Fig. 8), which has Δ*G*^‡^ = 15.7 kcal mol^−1^ with M06L/def2-TZVPP and 19.4 kcal mol^−1^ with DLPNO-CCSD(T)/def2-TZVPP, leading to intermediate 15. This stepwise insertion/rearrangement process is thermoneutral (Δ*G* = −0.9 kcal mol^−1^) for the septet state. 15 is a dicobalt(ii) carbonate complex with a CO ligand coordinated to one metal center, which is readily transferred to 1 to afford the final products. Comparison of the barriers required for C–O bond breaking as computed for the three pathways shows that the reaction of CO_2_ with L^*t*Bu^Co is not likely to follow the mononuclear dissociative pathway, while either of the dinuclear pathways is possible because the computed barriers for TS4 and TS7 are similar, especially those calculated by the more accurate DLPNO-CCSD(T) approach. In contrast to the mononuclear intermediate 5, which does not undergo an associative mechanism, intermediate 10 contains two cobalt(ii) centers bridged by a CO_2_^2−^ anion (discussed below on the basis of frontier orbitals) and facilitates further CO_2_ addition.

For both dinuclear pathways, free energy barriers calculated with B3LYP and M06L functionals employing the def-TZVPP basis set and conducted using the CPCM solvation model were in good agreement with the DLPNO-CCSD(T) benchmark for important transformations, like C–O bond-cleavage *via*TS2. A similar situation is also observed for TS4 and TS7 (Table 3). Hence, apart from the ligand isomerization step (TS1), our theoretical results are analogous to those computed by the coupled-cluster approach and are evidently *not* functional dependent.

#### Frontier-orbital analysis of C–O cleavage in different reaction pathways

**Fig. 9:**
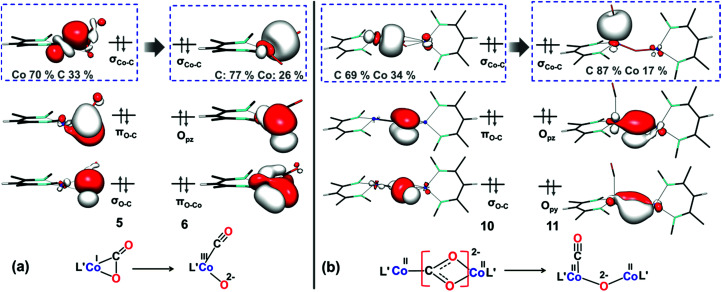
Important orbitals during C–O cleavage mediated by the (a) mononuclear dissociative pathway and (b) dinuclear dissociative pathway at the M06L/def2-TZVPP level of theory.

As shown in Fig. 9a using the M06L/def2-TZVPP technique, coordination of CO_2_ to 1*′* in the mononuclear complex 5 only slightly perturbs the σ and π interactions within CO_2_, because the doubly occupied orbital termed σ_Co–C_ (consisting of the Co d_*z*^2^_ and CO_2_ π* orbitals) contains a mere 30% carbon contribution. This indicates that there is only partial electron transfer from the metal center to CO_2_, consistent with the difference of only 0.06 Å between the two C–O bond distances (Fig. 5). During the transformation of 5 to 6, both σ and π bonds undergo heterolytic cleavage, which is accompanied by two-electron transfer from the Co d_*z*^2^_ orbital to the CO_2_ π* orbital. As a consequence, the CO_2_ π* orbital eventually transforms into the CO lone pair in complex 6, and the cobalt(i) center in 5 is oxidized to cobalt(iii) (Fig. 9a). This is reminiscent of the C–F bond cleavage by 1 which was studied using kinetics^33^ and computations^71^ that indicated oxidative addition of the C–F bond to give a four-coordinate cobalt(iii) product. However, the fluoride-containing product of C–F activation is much more stable than the oxo-containing product of C–O activation, and therefore *the mononuclear pathway is disfavored for CO*_*2*_*cleavage*.

In the dicobalt intermediate 10, the CO_2_ moiety is reduced by nearly two electrons, with each metal center donating one electron (Fig. 9b), and the Co–C σ-bonding orbital is predominantly carbon based. The greater electron transfer from the metal to CO_2_ in 10 relative to 5 reflects the greater ease of oxidizing each metal by only one electron. Furthermore, the dinuclear dissociative pathway entails lower reorganization energy during C–O bond cleavage, because the oxidation states of the metal centers do not change. As shown in ESI, Section Q,‡ the conversion of 14 to 15*via*TS7 in the dinuclear associative pathway involves similar electronic structure changes. The target C–O σ-bond cleavage in TS7 results in conversion of the bonding orbital to the oxygen p orbital (Fig. S19‡). Concurrently the same oxygen starts interacting with one cobalt(ii) center to form a new Co–O bond. This cooperation between the two metals facilitates C–O bond cleavage and makes the bimetallic pathways dominant.

### Literature perspectives

The reaction of CO_2_ with L^*t*Bu^Co to give 2 and 4 represents the net reductive cleavage of two molecules of CO_2_ to CO and CO_3_^2−^. Chemical fixation and transformation of CO_2_ has been reported in many earlier studies of dinuclear macrocyclic complexes where CO_2_ is reduced to form carbonate and CO.^72–75^ One of the earliest studies by Sorrell *et al.* included crystallographic characterization of a carbonate-bridged dinuclear copper complex from the reaction of CO_2_ with a peroxo-dicopper(ii) intermediate.^72^ Homogeneous electrocatalytic CO_2_ reduction to form CO was originally reported in a well-characterized cobalt system by Eisenberg^76^ and developed in the research of DuBois^77,78^ and Kubiak,^79^ and many further advances in cobalt-based CO_2_ reduction to CO have been described by Fujita and others.^20,23–25,30^ This continues to be an active area of research by many groups, and therefore mechanistic insights are important.^13,26–29,31^

**Scheme 4:**
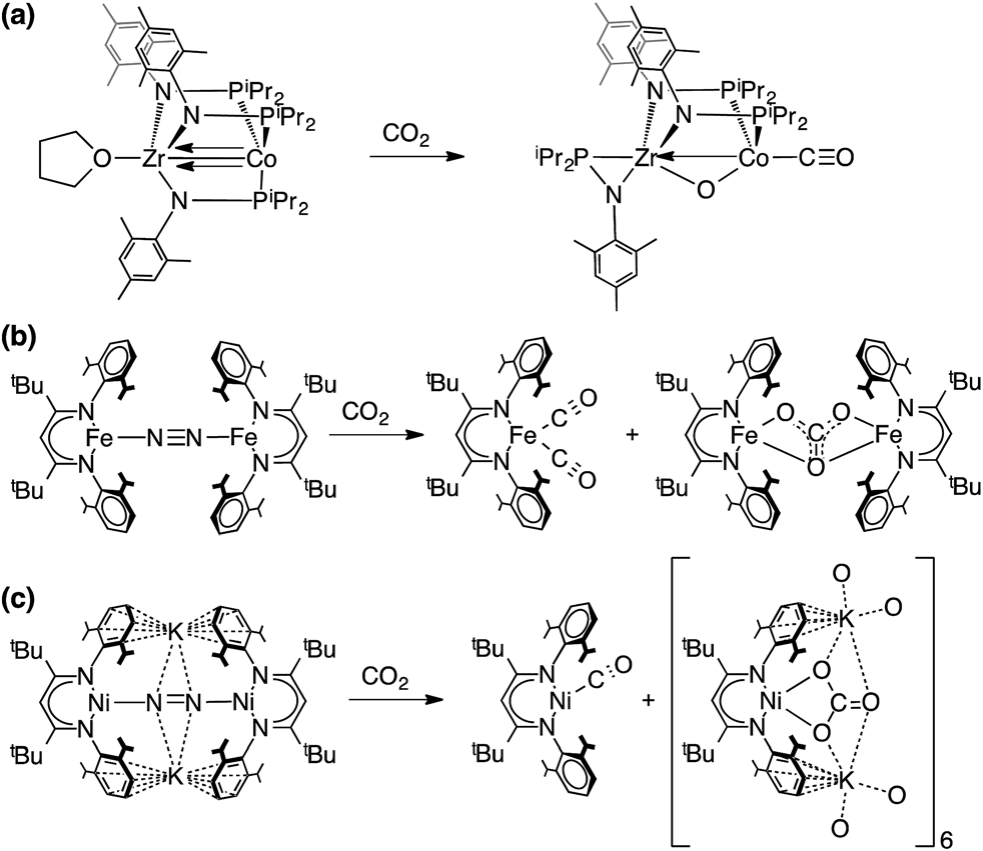
Selected CO_2_ reduction reactions from the literature.

Stoichiometric reduction of CO_2_ to CO using well-defined late 3d metal systems is also relevant. Peters reported that a Fe tris(phosphino)borate complex reacts with CO_2_ to give binuclear μ-O:μ-CO and C–C coupled μ-oxalato complexes, wherein the relative yields of the two products can be controlled by the choice of solvent.^80,81^ CO_2_ reduction to CO using a pyridinediimine stabilized iron(ii) precatalyst has also been reported.^82^ Sadighi *et al.* identified a unique CO_2_/CO bridged dinuclear (NHC)Ni complex.^83^ A particularly cogent precedent is the heterobimetallic cobalt complex Co(^i^Pr_2_PNMes)_3_Zr(THF), which cleaves CO_2_ to CO and oxo (Scheme 4a).^24,84^

The reaction described here between CO_2_ and the cobalt(i) source L^*t*Bu^Co can be compared to the published reactions of CO_2_ with the iron(i) source L^*t*Bu^FeNNFeL^*t*Bu^ (Scheme 4b),^37^ the nickel(i) source L^*t*Bu^NiNNNiL^*t*Bu^ and the nickel(0) source K_2_[L^*t*Bu^NiNNNiL^*t*Bu^] (Scheme 4c).^85^ The carbonate products from the Fe and Co reactions are very similar, but the CO products are different. While the iron dinitrogen complex gave the dicarbonyl complex L^*t*Bu^Fe(CO)_2_,^37^ the cobalt analogue gave instead the monocarbonyl complex L^*t*Bu^Co(CO). This difference can be attributed to the extent of backbonding. L^*t*Bu^Fe(CO)_2_ displays lower CO stretching frequencies (*ν*_CO_ = 1994, 1915 cm^−1^) as compared to L^*t*Bu^Co(CO)_2_ (*ν*_CO_ = 2014, 1949 cm^−1^), and thus backbonding is weaker in L^*t*Bu^Co(CO)_2_ than in the analogous Fe complex. Consistent with weaker bonding in the cobalt dicarbonyl *versus* the iron dicarbonyl, reaction of L^*t*Bu^Co(CO)_2_ with L^*t*Bu^Co afforded the monocarbonyl complex, whereas L^*t*Bu^Fe(CO) is unknown. Thus, we surmise that the difference in the product distribution between iron and cobalt arises from the relative M–C bond energy of the second CO. In the nickel case, Horn *et al.* reported two related reactions.^85^ First, the reaction of the dinickel(i) complex L^*t*Bu^NiNNNiL^*t*Bu^ with CO_2_ resulted in reductive coupling to form an oxalate complex (Ni^2+^C_2_O_4_Ni^2+^), which was not observed in the analogous cobalt system. We speculate that the smaller nickel(i) center cannot access a L^*t*Bu^NiONiL^*t*Bu^ intermediate and thus is diverted to C–C coupling. On the other hand, reaction of the nickel(0) complex K_2_[L^*t*Bu^NiNNNiL^*t*Bu^] with CO_2_ gave a nickel(i)–CO complex and a nickel(ii) complex of CO_3_^2−^, which is more similar to the results described here.

It is also worthwhile to compare our results to the electrochemical reduction of CO_2_ to CO catalyzed by [Ni(cyclam)]^+^, which we have previously examined computationally.^86^ These studies showed that formation of an η^1^-CO_2_ adduct [Ni(η^1^-CO_2_)(cyclam)]^+^ causes partial electron transfer from the metal center to CO_2_, similar to complex 5 in the present work. The second electron reduction of CO_2_ in the [Ni(cyclam)]^+^ case requires proton donors, where Lewis acid stabilization of [Ni^II^(C(O)OH)(cyclam)]^+^ enables facile C–O bond cleavage to generate [Ni(CO)(cyclam)]^2+^ and H_2_O. Lewis acid stabilization facilitating CO_2_ cleavage has also been shown clearly in iridium and iron systems by Bernskoetter and Hazari.^87,88^ Here, the second cobalt stabilizes the growing negative charge on the bound CO_2_ like a Lewis acid; additionally, the second cobalt in the system described here provides a second electron that is crucial for CO_2_ reduction.

## Conclusions

The masked two-coordinate cobalt(i) complex L^*t*Bu^Co cleaves the C–O bond in CO_2_ to form a mononuclear carbonyl complex and a dinuclear carbonate complex. Both DFT and the highly correlated wavefunction-based DLPNO-CCSD(T) calculations show two feasible bimetallic pathways, and the dissociative one is supported by the independent isolation of a kinetically competent dicobalt(ii) oxo complex. The electronic structure of this unusual oxo complex was analyzed using the multi-reference CASSCF approach, which showed that the oxo compound is best described as a dicobalt(ii) complex, in contrast with an earlier literature compound with redox-active oxyl radical character. The negatively charged oxygen in the new dicobalt(ii) oxo compound has nucleophilicity that leads to its rapid reactivity toward carbon dioxide. Overall, the cooperative interactions of the two cobalt centers facilitate charge buildup on CO_2_, leading to facile activation of this strong bond.

## Conflicts of interest

The authors declare no conflicts of interest.

## Supplementary Material

SC-010-C8SC02599A-s001

SC-010-C8SC02599A-s002

## References

[cit1] Nam W., Lee Y. M., Fukuzumi S. (2014). Acc. Chem. Res..

[cit2] Oloo W. N., Que Jr L. (2015). Acc. Chem. Res..

[cit3] Kaizer J., Klinker E. J., Oh N. Y., Rohde J.-U., Song W. J., Stubna A., Kim J., Münck E., Nam W., Que Jr L. (2004). J. Am. Chem. Soc..

[cit4] Hong S., So H., Yoon H., Cho K. B., Lee Y. M., Fukuzumi S., Nam W. (2013). Dalton Trans..

[cit5] Neu H. M., Yang T., Baglia R. A., Yosca T. H., Green M. T., Quesne M. G., de Visser S. P., Goldberg D. P. (2014). J. Am. Chem. Soc..

[cit6] Das D., Pattanayak S., Singh K. K., Garai B., Sen Gupta S. (2016). Chem. Commun..

[cit7] Nguyen A. I., Ziegler M. S., Ona-Burgos P., Sturzbecher-Hohne M., Kim W., Bellone D. E., Tilley T. D. (2015). J. Am. Chem. Soc..

[cit8] Vincent J. B., Olivier-Lilley G. L., Averill B. A. (1990). Chem. Rev..

[cit9] Baik M.-H., Newcomb M., Friesner R. A., Lippard S. J. (2003). Chem. Rev..

[cit10] Bu W.-M., Ye L., Yang G.-Y., Gao J.-S., Fan Y.-G., Shao M.-C., Xu J.-Q. (2001). Inorg. Chem. Commun..

[cit11] Yonke B. L., Reeds J. P., Zavalij P. Y., Sita L. R. (2011). Angew. Chem., Int. Ed..

[cit12] Ying W. L., Emerson J., Clarke M. J., Sanadi D. R. (1991). Biochemistry.

[cit13] Guo Z., Cheng S., Cometto C., Anxolabéhère-Mallart E., Ng S.-M., Ko C.-C., Liu G., Chen L., Robert M., Lau T.-C. (2016). J. Am. Chem. Soc..

[cit14] Kanan M. W., Yano J., Surendranath Y., Dincă M., Yachandra V. K., Nocera D. G. (2010). J. Am. Chem. Soc..

[cit15] Kanan M. W., Surendranath Y., Nocera D. G. (2009). Chem. Soc. Rev..

[cit16] Artero V., Chavarot-Kerlidou M., Fontecave M. (2011). Angew. Chem., Int. Ed..

[cit17] Stauber J. M., Bloch E. D., Vogiatzis K. D., Zheng S. L., Hadt R. G., Hayes D., Chen L. X., Gagliardi L., Nocera D. G., Cummins C. C. (2015). J. Am. Chem. Soc..

[cit18] Zhang R.-L., Zhao J.-S., Xi X.-L., Yang P., Shi Q.-Z. (2008). Chin. J. Chem..

[cit19] Appel A. M., Bercaw J. E., Bocarsly A. B., Dobbek H., DuBois D. L., Dupuis M., Ferry J. G., Fujita E., Hille R., Kenis P. J. A., Kerfeld C. A., Morris R. H., Peden C. H. F., Portis A. R., Ragsdale S. W., Rauchfuss T. B., Reek J. N. H., Seefeldt L. C., Thauer R. K., Waldrop G. L. (2013). Chem. Rev..

[cit20] Wang W.-H., Himeda Y., Muckerman J. T., Manbeck G. F., Fujita E. (2015). Chem. Rev..

[cit21] Francke R., Schille B., Roemelt M. (2018). Chem. Rev..

[cit22] Fujita E., Furenlid L. R., Renner M. W. (1997). J. Am. Chem. Soc..

[cit23] Ogata T., Yanagida S., Brunschwig B. S., Fujita E. (1995). J. Am. Chem. Soc..

[cit24] Yao S. A., Ruther R. E., Zhang L. H., Franking R. A., Hamers R. J., Berry J. F. (2012). J. Am. Chem. Soc..

[cit25] Shaffer D. W., Johnson S. I., Rheingold A. L., Ziller J. W., Goddard W. A., Nielsen R. J., Yang J. Y. (2014). Inorg. Chem..

[cit26] Morlanés N., Takanabe K., Rodionov V. (2016). ACS Catal..

[cit27] Hu X. M., Ronne M. H., Pedersen S. U., Skrydstrup T., Daasbjerg K. (2017). Angew. Chem., Int. Ed..

[cit28] Ouyang T., Huang H.-H., Wang J.-W., Zhong D.-C., Lu T.-B. (2017). Angew. Chem., Int. Ed..

[cit29] Takeda H., Cometto C., Ishitani O., Robert M. (2017). ACS Catal..

[cit30] Shimoda T., Morishima T., Kodama K., Hirose T., Polyansky D. E., Manbeck G. F., Muckerman J. T., Fujita E. (2018). Inorg. Chem..

[cit31] Chapovetsky A., Welborn M., Luna J. M., Haiges R., Miller T. F., Marinescu S. C. (2018). ACS Cent. Sci..

[cit32] Dugan T. R., Sun X., Rybak-Akimova E. V., Olatunji-Ojo O., Cundari T. R., Holland P. L. (2011). J. Am. Chem. Soc..

[cit33] Dugan T. R., Goldberg J. M., Brennessel W. W., Holland P. L. (2012). Organometallics.

[cit34] DeRosha D. E., Mercado B. Q., Lukat-Rodgers G., Rodgers K. R., Holland P. L. (2017). Angew. Chem., Int. Ed..

[cit35] Al-Afyouni M. H., Suturina E., Pathak S., Atanasov M., Bill E., DeRosha D. E., Brennessel W. W., Neese F., Holland P. L. (2015). J. Am. Chem. Soc..

[cit36] Sadique A. R., Brennessel W. W., Holland P. L. (2009). Acta Crystallogr., Sect. C: Cryst. Struct. Commun..

[cit37] Sadique A. R., Brennessel W. W., Holland P. L. (2008). Inorg. Chem..

[cit38] Dai X. L., Kapoor P., Warren T. H. (2004). J. Am. Chem. Soc..

[cit39] Holland P. L., Cundari T. R., Perez L. L., Eckert N. A., Lachicotte R. J. (2002). J. Am. Chem. Soc..

[cit40] Ding K., Holland P. L., Adhikari D., Mindiola D. J. (2010). Inorg. Synth..

[cit41] Corcos A. R., Villanueva O., Walroth R. C., Sharma S. K., Bacsa J., Lancaster K. M., MacBeth C. E., Berry J. F. (2016). J. Am. Chem. Soc..

[cit42] Eckert N. A., Stoian S., Smith J. M., Bominaar E. L., Münck E., Holland P. L. (2005). J. Am. Chem. Soc..

[cit43] Groves J. T., Roman J. S. (1995). J. Am. Chem. Soc..

[cit44] Tolman W. B. (2010). Angew. Chem., Int. Ed..

[cit45] Bar-Nahum I., Gupta A. K., Huber S. M., Ertem M. Z., Cramer C. J., Tolman W. B. (2009). J. Am. Chem. Soc..

[cit46] Kupper C., Mondal B., Serrano-Plana J., Klawitter I., Neese F., Costas M., Ye S., Meyer F. (2017). J. Am. Chem. Soc..

[cit47] Holland P. L. (2008). Acc. Chem. Res..

[cit48] Holland P. L. (2015). Acc. Chem. Res..

[cit49] Shaik S., Hirao H., Kumar D. (2007). Acc. Chem. Res..

[cit50] Schäfer A., Huber C., Ahlrichs R. (1994). J. Chem. Phys..

[cit51] Schäfer A., Horn H., Ahlrichs R. (1992). J. Chem. Phys..

[cit52] Tomasi J., Mennucci B., Cammi R. (2005). Chem. Rev..

[cit53] Decker A., Rohde J.-U., Que Jr L., Solomon E. I. (2004). J. Am. Chem. Soc..

[cit54] Decker A., Clay M. D., Solomon E. I. (2006). J. Inorg. Biochem..

[cit55] Mondal B., Roy L., Neese F., Ye S. (2016). Isr. J. Chem..

[cit56] Ye S., Geng C.-Y., Shaik S., Neese F. (2013). Phys. Chem. Chem. Phys..

[cit57] Ye S., Neese F. (2011). Proc. Natl. Acad. Sci. U. S. A..

[cit58] Ye S., Neese F. (2009). Curr. Opin. Chem. Biol..

[cit59] Mondal B., Neese F., Ye S. (2015). Inorg. Chem..

[cit60] Mondal B., Neese F., Ye S. (2016). Inorg. Chem..

[cit61] Zhao Y., Truhlar D. G. (2006). J. Chem. Phys..

[cit62] Castro L., Lam O. P., Bart S. C., Meyer K., Maron L. (2010). Organometallics.

[cit63] Kim Y. E., Kim J., Lee Y. (2014). Chem. Commun..

[cit64] Anderson J. S., Iluc V. M., Hillhouse G. L. (2010). Inorg. Chem..

[cit65] Liu C., Munjanja L., Cundari T. R., Wilson A. K. (2010). J. Phys. Chem. A.

[cit66] Liu C., Cundari T. R., Wilson A. K. (2011). Inorg. Chem..

[cit67] Ariafard A., Brookes N. J., Stranger R., Boyd P. D., Yates B. F. (2010). Inorg. Chem..

[cit68] Agarwal J., Fujita E., Schaefer III H. F., Muckerman J. T. (2012). J. Am. Chem. Soc..

[cit69] Kirchner B., Wennmohs F., Ye S., Neese F. (2007). Curr. Opin. Chem. Biol..

[cit70] Noodleman L. (1981). J. Chem. Phys..

[cit71] Jiang Q., Cundari T. R. (2017). Comput. Theor. Chem..

[cit72] Sorrell T. N., Allen W. E., White P. S. (1995). Inorg. Chem..

[cit73] Haines R. J., Wittrig R. E., Kubiak C. P. (1994). Inorg. Chem..

[cit74] Chen J.-M., Wei W., Feng X.-L., Lu T.-B. (2007). Chem.–Asian J..

[cit75] Notni J., Schenk S., Görls H., Breitzke H., Anders E. (2008). Inorg. Chem..

[cit76] Fisher B., Eisenberg R. (1980). J. Am. Chem. Soc..

[cit77] Raebiger J. W., Turner J. W., Noll B. C., Curtis C. J., Miedaner A., Cox B., DuBois D. L. (2006). Organometallics.

[cit78] Rakowski Dubois M., Dubois D. L. (2009). Acc. Chem. Res..

[cit79] Benson E. E., Kubiak C. P., Sathrum A. J., Smieja J. M. (2009). Chem. Soc. Rev..

[cit80] Lu C. C., Saouma C. T., Day M. W., Peters J. C. (2007). J. Am. Chem. Soc..

[cit81] Saouma C. T., Lu C. C., Day M. W., Peters J. C. (2013). Chem. Sci..

[cit82] Thammavongsy Z., Seda T., Zakharov L. N., Kaminsky W., Gilbertson J. D. (2012). Inorg. Chem..

[cit83] Lee C. H., Laitar D. S., Mueller P., Sadighi J. P. (2007). J. Am. Chem. Soc..

[cit84] Krogman J. P., Foxman B. M., Thomas C. M. (2011). J. Am. Chem. Soc..

[cit85] Horn B., Limberg C., Herwig C., Braun B. (2013). Chem. Commun..

[cit86] Song J., Klein E. L., Neese F., Ye S. (2014). Inorg. Chem..

[cit87] Schmeier T. J., Dobereiner G. E., Crabtree R. H., Hazari N. (2011). J. Am. Chem. Soc..

[cit88] Bernskoetter W. H., Hazari N. (2017). Acc. Chem. Res..

