# Supplementary material for: Reduction of CO2 by a masked two-coordinate cobalt(i) complex and characterization of a proposed oxodicobalt(ii) intermediate
Source: Chem Sci. 2018 Nov 9;10(3):918–29. doi: 10.1039/c8sc02599a (PMC6346294; doi:10.1039/c8sc02599a)
Supplement: SC-010-C8SC02599A-s001 [file SC-010-C8SC02599A-s001.pdf]

Electronic Supplementary Material (ESI) for Chemical Science.
This journal is © The Royal Society of Chemistry 2018

# Electronic Supporting Information

# Reduction of $CO_2$ by a Masked Two-Coordinate Cobalt(I) Complex and Characterization of a Proposed Oxodicobalt(II) Intermediate

*Lisa Roy,[a,g,†] Malik H. Al-Afyouni,[b,†] Daniel E. DeRosha,[c,†] Bhaskar Mondal,[a] Ida M. DiMucci,[d] Kyle M. Lancaster,[d] Jason Shearer,[e] Eckhard Bill,[a] William W. Brennessel,[b] Frank Neese,[f] Shengfa Ye,[f,]* and Patrick L. Holland[c,]**

[a] *Max Planck Institute for Chemical Energy Conversion, Stiftstraße 34-36, Mülheim an der Ruhr, D-45470, Germany.*
[b] *Department of Chemistry, University of Rochester, Rochester, New York 14618, USA.*
[c] *Department of Chemistry, Yale University, New Haven, Connecticut 06520, USA.*
[d] *Department of Chemistry and Chemical Biology, Baker Laboratory, Cornell University, Ithaca, New York 14853, USA.*
[e] *Department of Chemistry, University of Nevada, Reno, Nevada 89557, USA.*
[f] *Max Planck Institute for Coal Research, Kaiser-Wilhelm-Platz 1, Mülheim an der Ruhr, D-45470, Germany*
[g] *CSIR Central Mechanical Engineering Research Institute, Durgapur 713209, India.*

† *These authors contributed equally.*

*Shengfa Ye, email: shengfa.ye@kofo.mpg.de

*Patrick L. Holland, email: patrick.holland@yale.edu

**Table of Contents**


| | |
|---|---|
| A. Experimental section | Page 3 |
| B. $^{1}H$ NMR spectra | Page 8 |
| C. NMR kinetics of $CO_2$ reduction by $L^{tBu}Co$ | Page 12 |
| D. Attempted synthesis of CO coordinated oxo complex **11** | Page 15 |
| E. X-ray absorbance spectroscopy of $L^{tBu}Co(O)CoL^{tBu}$ (**3**) and reference compounds | Page 16 |
| F. X-ray crystallography | Page 17 |
| G. Justification for truncating the computational ligand model | Page 19 |
| H. Benchmarking the various density functional methods with DLPNO-CCSD(T) | Page 20 |
| I. Different bridging binding modes of $CO_2$ with two metal complexes and their geometrical parameters | Page 21 |
| J. Magneto-structural isomers of intermediate **10** | Page 22 |
| K. B3LYP calculated free energy profiles | Page 23 |
| L. M06L calculated free energy profiles | Page 24 |
| M. Optimized geometry of important intermediates and transition states at BP86/B1 | Page 25 |
| N. XYZ coordinates of optimized geometries | Page 26 |
| O. Comparison of diketiminate bond metrics between **3** and $L^{tBu}CoCl$ | Page 30 |
| P. Electronic structure of **3** from multi-configurational calculations. | Page 31 |
| Q. Frontier orbital analysis during dinuclear associative pathway | Page 35 |
| R. Thermodynamics of additional pathways with mononuclear species | Page 35 |
| S. Why the mononuclear associative pathway is not predicted | Page 36 |
| T. Total energies predicted at M06L level of theory | Page 38 |
| U. Electronic Structure of Co-O-Co at different levels of Density Functional Theory | Page 40 |
| V. References | Page 44 |

## A. Experimental Section

**General Considerations**. All synthetic manipulations were performed under an argon atmosphere using standard Schlenk techniques or in an M. Braun glovebox maintained at or below 1 ppm $O_2$ and $H_2O$. Glassware was dried at 150 °C overnight. Hexane and toluene were purified by passage through activated alumina and Q5 columns from Glass Contour Co. (Laguna Beach, CA). Celite was dried at 300 °C under vacuum overnight. THF was dried by distilling from Na/benzophenone. THF-$d_8$, $C_6D_{12}$, and $C_6D_6$ were dried over $CaH_2$ and then over Na/benzophenone and vacuum transferred and stored over 3 Å molecular sieves. $CO_2$, CO, and $N_2O$ were purchased from Airgas, Inc. and passed through a column of activated alumina. $^{13}CO$ was purchased from Cambridge Isotope Laboratories, Inc. $L^{tBu}CoCl$,[1] $L^{tBu}Co$,[2] and $L^{tBu}Co(CO)_2$[2,3] were synthesized according to published procedures.

$^1H$ NMR spectra were recorded on a Bruker Avance 500 MHz spectrometer. All spectra are referenced to residual protiated solvent signals: $C_6D_6$ (7.16 ppm), THF-$d_8$ (3.58 or 1.73 ppm), $C_6D_{12}$ (1.38 ppm). The NMR probe temperature was calibrated using methanol. IR spectra were recorded on a Shimadzu FTIR spectrophotometer (FTIR-8400S). UV-vis absorption spectra were recorded on a Cary 50 or Cary 60 spectrophotometer using Teflon-sealed quartz cuvettes. Elemental analyses were obtained from the CENTC Elemental Analysis Facility at the University of Rochester. Microanalysis samples were handled under argon, weighed with a PerkinElmer Model AD-6 Autobalance, and their compositions were determined with a PerkinElmer 2400 Series II Analyzer.

**Reaction of $L^{tBu}Co$ with $CO_2$.** A resealable flask was charged with $L^{tBu}Co$ (158 mg, 0.28 mmol) and hexane (15 mL). The dark solution was frozen with liquid $N_2$ and the headspace of the flask was evacuated. While submerged in liquid $N_2$, $CO_2$ (7.73 mL at 891 mBar, 0.28 mmol) was introduced to the flask and allowed to condense for 30 min. The solution was allowed to thaw at room temperature before stirring at 60 °C for 11 h to give a red solution.

The solution was filtered through Celite, and volatiles were removed to give a red solid mixture of **2** and **4**. Cold pentane (5 mL) was used to extract **2**. The volume was reduced to ~2 mL, and cooling the solution to -40 °C caused crystalline **2** to precipitate (36 mg, 66%). The spectroscopic characteristics of **2** ($L^{tBu}$CoCO) matched the literature.[3] The remaining solid residue was extracted with toluene (10 mL) and the volume was reduced to ~5 mL. Cooling to -40 °C gave crystals of **4** (44 mg, 40%). Characterization of **4**: $^1$H NMR (500 MHz, $C_6D_6$): δ 26.8 (36H, t-Bu), 23.0 (8H, *m*-Ar), -4.8 (12H, iPr-C$H_3$), -6.9 (2H, backbone-C*H*), -41.0 (4H, iPr-C*H*), -45.4 (4H, *p*-Ar), -48.8 (12H, iPr-C$H_3$). Anal. Calcd. for $C_{71}H_{106}N_4O_3Co_2$: C, 72.22; H, 9.05; N, 4.74. Found: C, 72.42; H, 9.09; N, 4.51.

**Synthesis of [$L^{tBu}$Co]$_2$(μ-O) (3).** A resealable flask was charged with $L^{tBu}$Co (348.0 mg, 0.6206 mmol) and hexanes (50 mL). The dark brown solution was frozen using a liquid $N_2$ bath, and the headspace of the flask was evacuated. While frozen, $N_2O$ (13.34 mL at 633 mbar, 0.341 mmol) was introduced to the flask and condensed for 30 min. The solution was thawed and stirred at -78 °C for 4 h and then at ambient temperature for 2 h, resulting in precipitation of a red-orange solid. The mixture was filtered through Celite to collect red-orange solid. This solid was washed with hexanes (20 mL), extracted into THF (30 mL), and dried under vacuum to afford crude **3** (228.6 mg, 65%). Analytically pure **3** was obtained by dissolving 76 mg of crude **3** in THF (10 mL), concentrating the red solution to 5 mL under vacuum, and storing at -40 °C overnight to yield crystals. Single crystals for crystallography were grown by slowly evaporating a concentrated toluene solution under reduced pressure. $^1$H NMR ($C_6D_6$): δ 34.0 (br), 12.8, -20.5 (br), -34.0 ppm. $\mu_{eff}$ ($C_6D_6$, 25 °C) 4.4(1) $\mu_B$. IR (KBr): 3049 (w), 2958 (s), 2922 (m), 2900 (m), 2865 (m), 1596 (w), 1539 (m), 1488 (m), 1457 (m), 1429 (m), 1383 (s), 1361 (s), 1309 (m), 1250 (m), 1202 (m), 1179 (m), 1152 (w), 1098 (m), 1018 (w) cm$^{-1}$. Anal. Calcd for $C_{70}H_{106}N_4OCo_2$: C, 73.92; H, 9.39; N, 4.92. Found: C, 73.54; H, 9.52; N, 4.54.

**NMR-scale reaction of 3 with $CO_2$.** A vial was charged with **3** (2.0 mg, 0.0018 mmol) and 1.0 mL $C_6D_{12}$. The orange mixture was stirred vigorously for 5 min before transfering 0.5 mL of this solution to a J. Young NMR tube containing a capillary with a nickelocene integration standard. The J. Young tube was sonicated for 1 min, and then the contents of the tube were flash frozen in a liquid $N_2$ cold bath. The headspace of the tube was evacuated. While frozen, $CO_2$ (13.34 mL at 5 mbar) was introduced to the tube and condensed for 30 min. The solution was thawed and then immediately injected into an NMR spectrometer cooled to 10 °C. NMR analysis using a nickelocene integration standard indicated formation of **4** in 75% spectroscopic yield (see Figure S5).

**Computational details.** Geometry optimizations utilized the unrestricted Kohn Sham theory and employed the GGA density functional BP86[4, 5] in combination with the triple-ζ quality TZVP[6] basis set on Co, O, N, and selected C atoms, and double ζ quality split-valence basis set, def2-SVP[7] on the rest of the atoms (B1 basis set). The broken-symmetry formalism developed by Noodleman[8, 9] was employed to describe states involving antiferromagnetic coupling. The following thresholds were used for optimizations: energy change tolerance of 5 $\times 10^{-6}$ a.u., root mean square gradient of $1 \times 10^{-4}$ a.u., maximum gradient of $3 \times 10^{-4}$ a.u., root mean square displacement of $2 \times 10^{-3}$ a.u., maximum displacement of $4 \times 10^{-3}$ a.u. The DFT integration grid was always set to Grid6 while the Hartree Fock exchange was evaluated by GridX6 for calculations with the B3LYP functional. Tight convergence criteria (energy tolerance $1 \times 10^{-8}$ a.u.) for self-consistent field (SCF) calculations were employed throughout all calculations. The atom-pairwise dispersion correction with Becke-Johnson damping (D3BJ), as implemented in ORCA[10, 11] was used throughout to treat non-covalent interactions. The resolution of identity (RI) approximation[12-14] was used to accelerate the calculations in conjunction with the auxiliary Coulomb-fitting basis set def2/J to accelerate

the calculations at the BP86 level. To account for relativistic effects, scalar relativistic zeroth order approximation (ZORA) was used.[15-18] Geometry optimizations were performed without any constraints. Solvent effects during optimization and single point calculations were modeled by employing the polarizable continuum model (CPCM)[19] using benzene ($\varepsilon$ = 2.3) as the solvent. Harmonic vibrational analyses were undertaken after the geometry optimization of each structure to differentiate local minima from transition states. The local minima were characterized by all positive eigenvalues of the Hessian matrix, whereas the transition states (TSs) have only one negative eigenvalue. The zero-point energies (ZPE), enthalpic corrections and entropic contributions were obtained from the frequency calculations. In order to obtain single-point energies closer to the basis set limit, B3LYP[4, 20] and M06L[21] calculations with the larger def2-TZVPP[22] basis set on all elements were carried out for which the auxiliary basis set def2-TZVPP/J was employed for the RIJCOSX or RI approximation as appropriate.[23] Empirical dispersion correction[24, 25] with the D3BJ keyword was employed all throughout except for calculations with M06L. The BP86 calculations on **TS4** and **11** (at S = 0) leads to an erroneous change in the local spin state of the two cobalt centers, deviating from the desired electronic structure. Since both species are involved in the rate-determining step of the dinuclear dissociative pathway, we re-optimized them (at S = 0 and 3 spin states) at the B3LYP/B1 level of theory. To be consistent, we optimized structures corresponding to rate-determining transition states and the reference intermediates/adducts among various pathways (such as **5, TS2, 6, 10, TS4, 11, 13, 14,** and **TS7)** at B3LYP/B1 and evaluated single point energies at M06L/def2-TZVPP. Reported relative free energies in this work are calculated at the M06L/def2-TZVPP level of theory unless otherwise mentioned. Finally, highly accurate electronic energies were computed by using open-shell DLPNO-CCSD(T)[26, 27] approach in conjunction with the def2-TZVPP basis set and an additional correlation fitting auxiliary basis set, def2-TZVPP/C. For pair natural orbital (PNO)

generation, the default values of *TCutPNO*, *TCutPairs* and *TCutMKN* were used.[28] Localized orbitals according to the Pipek-Mezey scheme were computed before PNO generation. Solvent corrections to coupled-cluster electronic energies were obtained from the M06L calculations. Due to the challenging computational cost, only crucial barriers were computed with coupled-cluster theory and the rest with M06L/def2-TZVPP. The gas phase Gibbs free energy change on addition/binding of $CO_2$ was corrected to the standard state (1 M) by using a correction factor of 1.89 kcal/mol.[29] This value is added to the relative free energy, when one molecule of $CO_2$ interacts with the mononuclear or dinuclear metal complexes to form the corresponding $CO_2$ adducts, which requires transfer of $CO_2$ from the gas phase into benzene solution.

To determine the electronic structure of **3**, we employed the multi-reference CASSCF[30, 31] approach.[32] The crystal structure of **3** was used as a starting point for geometry optimization, and to reduce computational cost, the two *tert*-butyl groups in the ligand were replaced with methyl groups. Geometry optimizations were performed using the BP86 functional and the B1 basis set combination. This approximation does not alter the electronic structure of the Co-O-Co moiety. The state-specific CASSCF (in the singlet spin state) calculations employed the triple-ζ quality def2-TZVPP basis set for all atoms with optimization of the first root. The active space consists of all 3d orbitals of both cobalt centers and the 2p orbitals of the bridging oxo ligand, which results in the active space CASSCF(20,13), 20 electrons distributed into 13 orbitals. Furthermore, to explore the effects of electronic excitations from filled π to vacant π* orbitals of the ligand, larger active spaces were constructed including the bonding and antibonding π-orbitals of the N-C-C-C-N of the β-diketiminate framework. This active space constituted of 24 electrons in 17 orbitals (CASSCF(24,17)). To make the computation of CASSCF(24,17) faster, the configuration interaction here has been treated with the ACCCI approximation.

## B. $^{1}H$ NMR spectra

**Figure S1.** $^{1}H$ NMR spectrum of **3** in $C_6D_6$ at 25 °C.

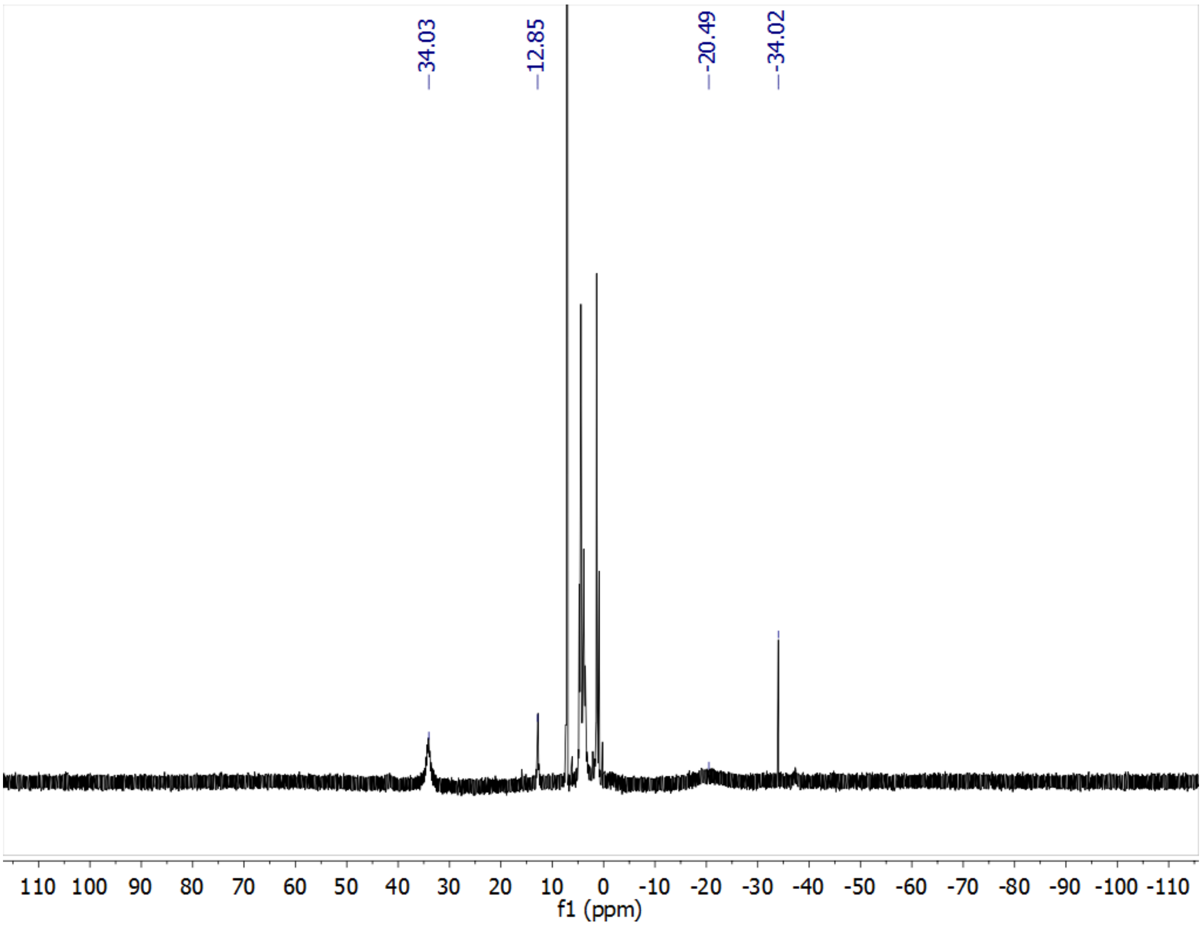

**Figure S2.** $^{1}$H NMR spectrum of **4** in $C_6D_6$ at 25 °C.

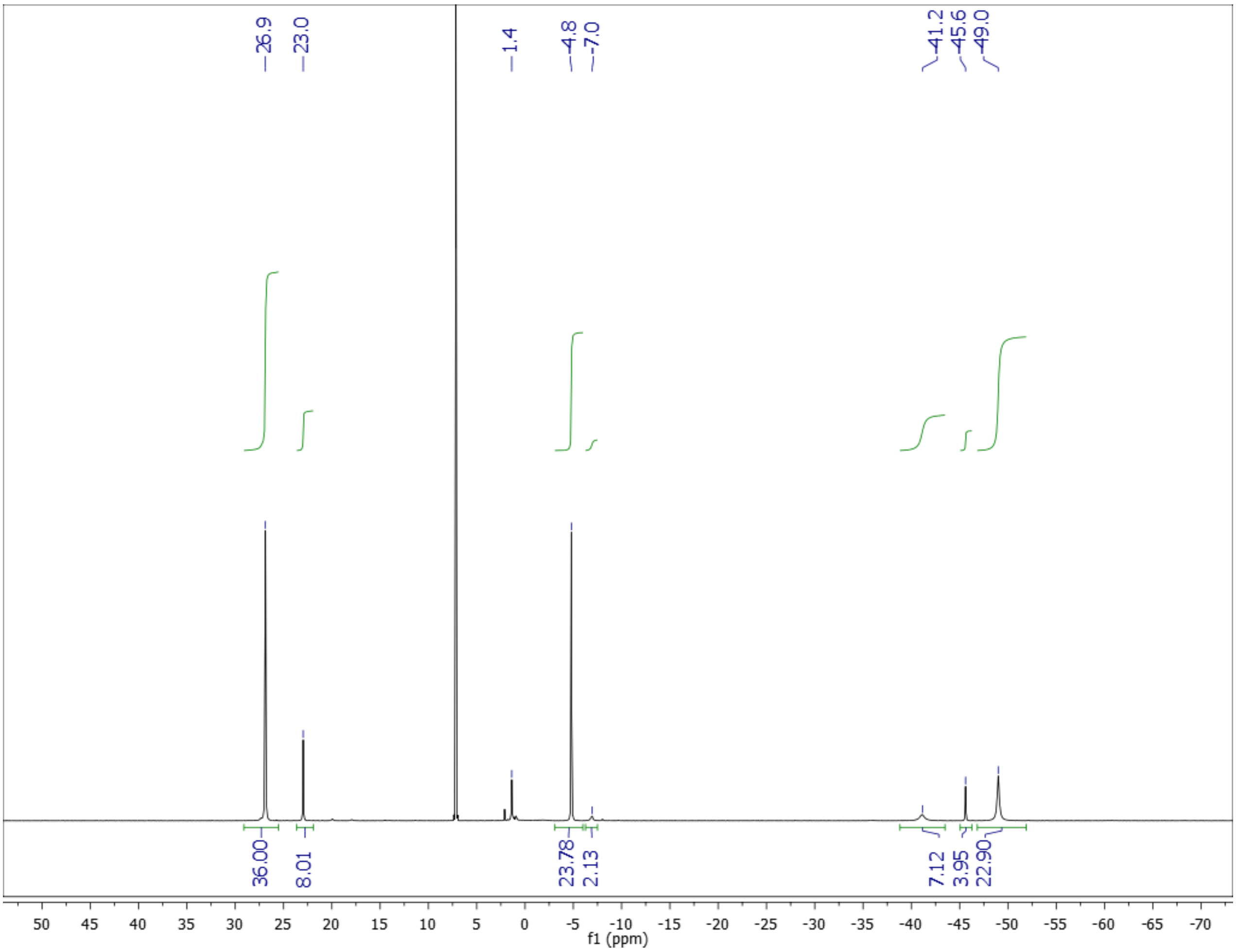

**Figure S3.** $^{1}H$ NMR spectra of reaction mixture of $L^{tBu}Co$ (**1**) with 10 equiv $CO_2$ monitored over time at 10 °C in $C_6D_{12}$. Integration with respect to a nickelocene internal standard shows that $L^{tBu}Co(CO)$ (**2**) is made in 95% spectroscopic and $L^{tBu}Co(OCO_2)CoL^{tBu}$ (**4**) is made in 86% spectroscopic yield after 3 h.

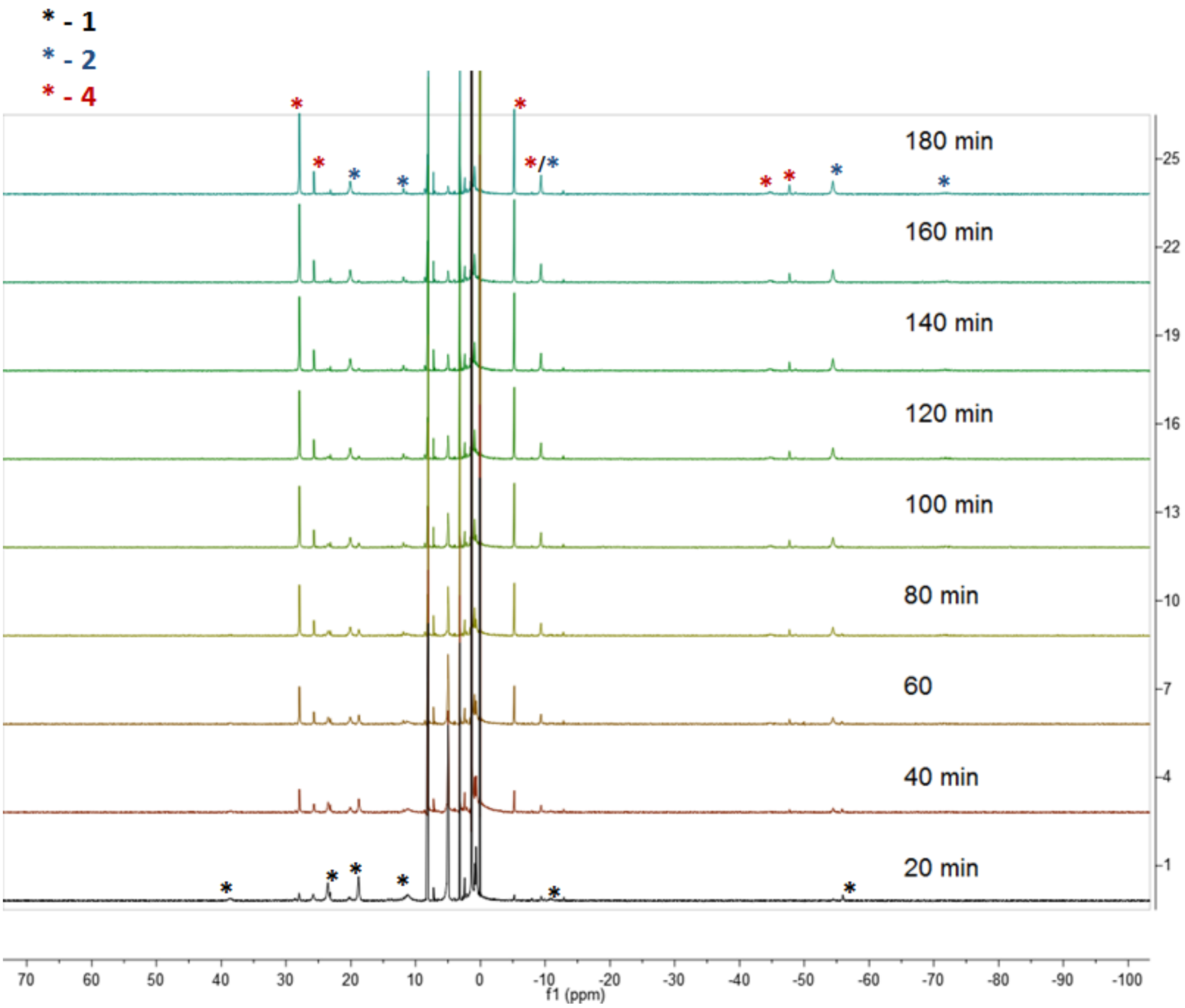

**Figure S4.** $^1$H NMR spectrum (25 °C) of crude product mixture from reaction of $L^{tBu}Co$ with 10 equiv of $CO_2$ in $C_6D_{12}$.

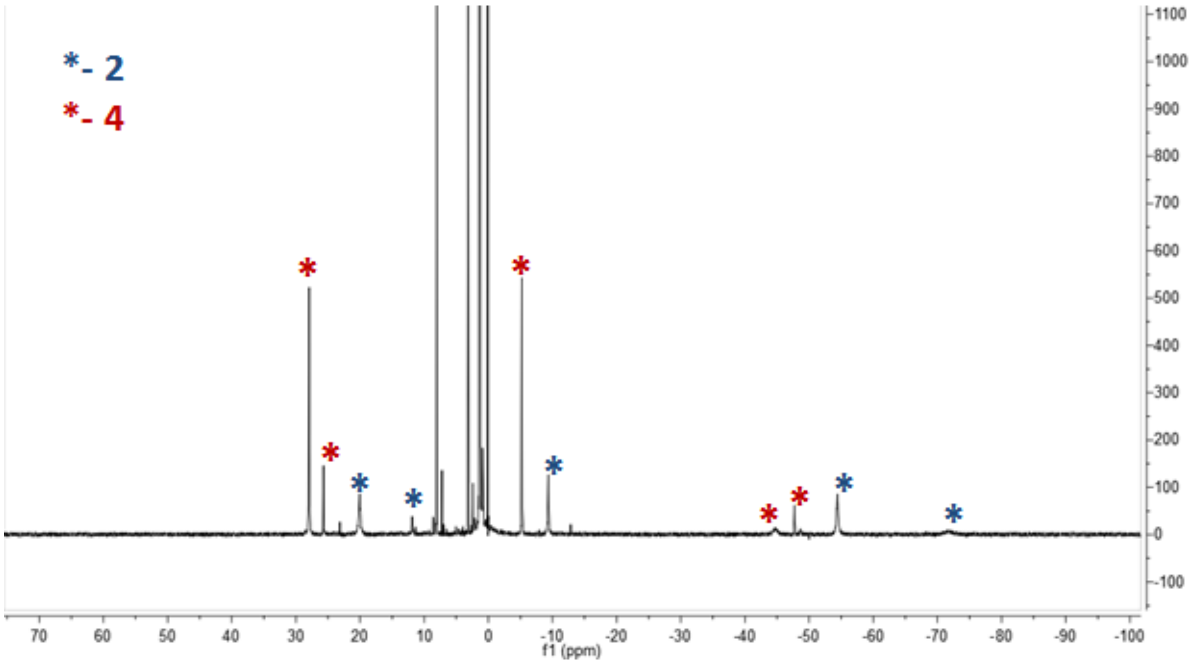


**Figure S5.** $^1$H NMR spectra of a reaction mixture of **3** before (below) and 2 min after (above) adding 1.5 equiv of $CO_2$ at 10 °C in $C_6D_{12}$. Integration relative to a nickelocene internal standard indicates formation of **4** in 75% spectroscopic yield.

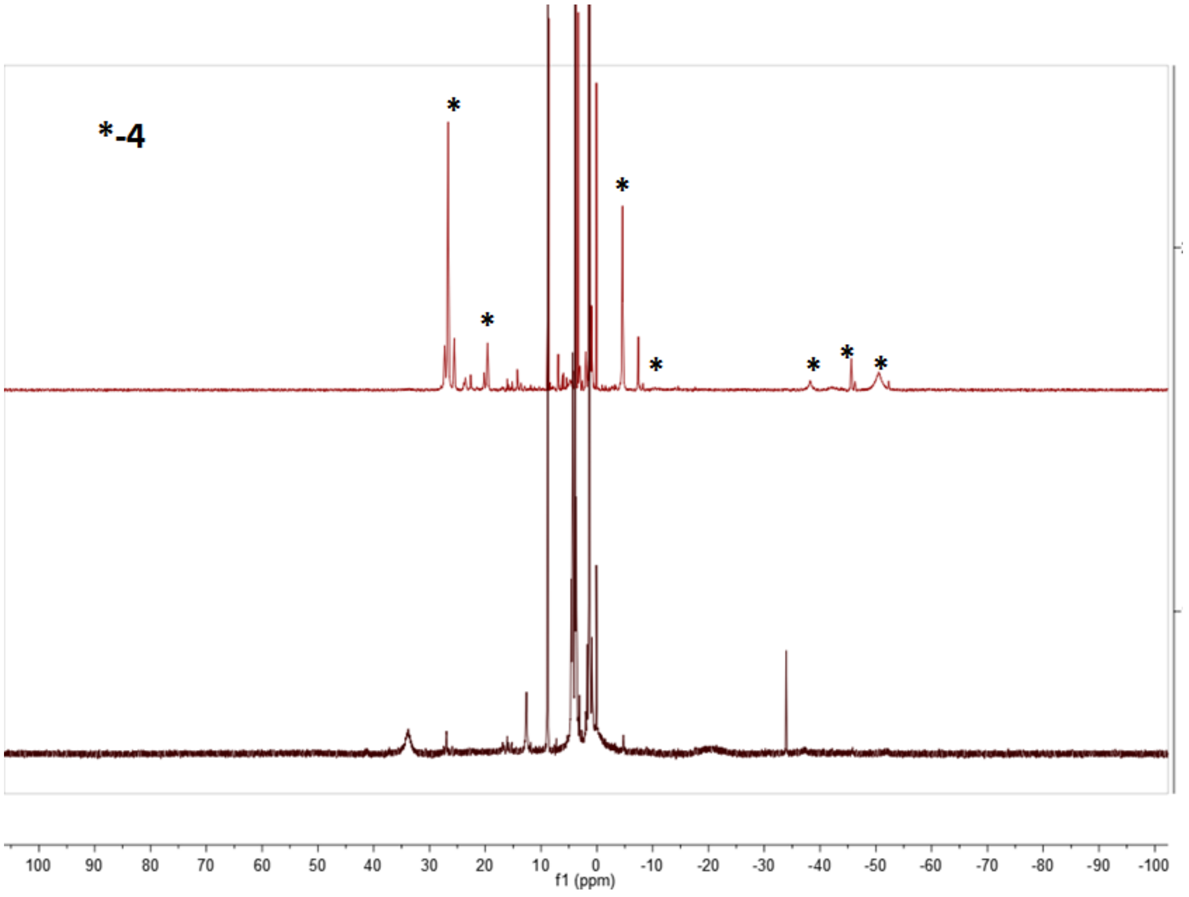

## C. NMR kinetics of $CO_2$ reduction by $L^{tBu}Co$.

A general procedure for one flooding concentration of $CO_2$ is described. Under an Ar atmosphere, a stock solution of **1** in $C_6D_{12}$ (0.200 mL, 13.4 mM, 0.00268 mmol), 0.400 mL of $C_6D_{12}$, and a nickelocene capillary integration standard were added to a J. Young NMR tube. The NMR tube was sealed with a rubber septum and the solution was flash frozen in a liquid $N_2$ bath. A stock solution of $CO_2$ in $C_6D_{12}$ (0.400 mL, 72.9 mM, 0.0292 mmol) was injected anaerobically by syringe. The solution was thawed and injected into an NMR spectrometer pre-cooled to 10 °C. Proton NMR spectra were acquired at 10°C every 10 min for 5 h.

This general procedure was followed to measure two different flooding concentrations of $CO_2$ as well as two initial concentrations of **1** as indicated below. The concentration over time profiles of $L^{tBu}Co$ (**1**), $L^{tBu}Co(CO)$ (**2**), and $L^{tBu}Co(\mu\text{-}OCO_2)CoL^{tBu}$ (**4**), as determined by integration of proton NMR data, were fit to first-order kinetics for each run. The value of the rate constant *k* for each run was then calculated as the average of the three observed rate constants obtained for a given experiment. Using these rate constants in the Eyring equation, a value of $\Delta G^{\ddagger}$=21.0 kcal/mol is obtained.

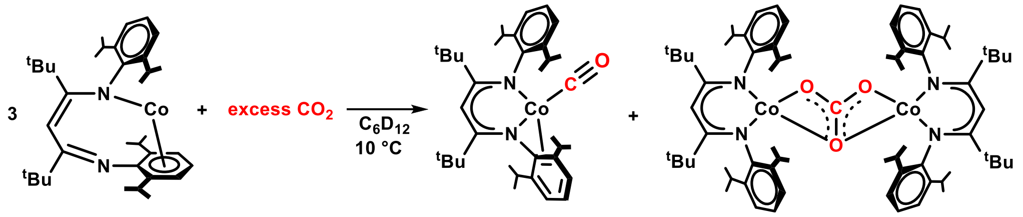


**Kinetics Conditions**

- **Run 1:** [LCo] = 2.67 mM, [$CO_2$] = 29.2 mM
- **Run 2:** [LCo] = 2.67 mM, [$CO_2$] = 58.4 mM
- **Run 3:** [LCo] = 5.34 mM, [$CO_2$] = 29.2 mM

| | LCo fit (k*$10^4$ $s^{-1}$) | LCo(CO) fit (k*$10^4$ $s^{-1}$) | LCo(OCO)CoL fit (k*$10^4$ $s^{-1}$) | Average (k*$10^4$ $s^{-1}$) |
|---|---|---|---|---|
| **Run 1** | 4.21 | 3.65 | 3.86 | 3.91 |
| **Run 2** | 3.79 | 3.81 | 3.55 | 3.72 |
| **Run 3** | 3.21 | 3.38 | 3.74 | 3.44 |

**Figure S6.** Reactant and product concentration over time data as determined by NMR spectroscopy in $C_6D_{12}$ at 10 °C; data and first-order fits correspond to Run 1 above.

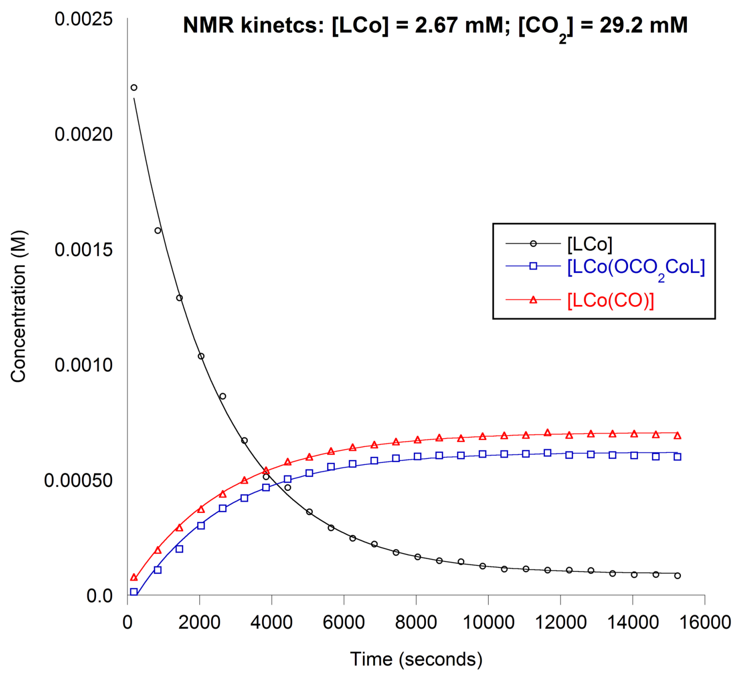


| y = m2*exp(-k*x)+m1 | | |
|---|---|---|
| | Value | Error |
| m1 | 0.00070648 | 2.1242e-6 |
| m2 | -0.00068437 | 5.519e-6 |
| k | 0.0003651 | 6.0158e-6 |
| Chisq | 8.8748e-10 | NA |
| R | 0.9994 | NA |

| y = m2*exp(-k*x)+m1 | | |
|---|---|---|
| | Value | Error |
| m1 | 0.00062019 | 4.3928e-6 |
| m2 | -0.00068194 | 1.2214e-5 |
| k | 0.00038628 | 1.3754e-5 |
| Chisq | 4.1404e-9 | NA |
| R | 0.9971 | NA |

| y = m2*exp(-k*x)+m1 | | |
|---|---|---|
| | Value | Error |
| m1 | 9.1137e-5 | 6.8343e-6 |
| m2 | 0.0022242 | 2.0918e-5 |
| k | 0.00042071 | 7.5843e-6 |
| Chisq | 1.1281e-8 | NA |
| R | 0.99922 | NA |

**Figure S7.** Reactant and product concentration over time as determined by NMR spectroscopy in $C_6D_{12}$ at 10 °C; data and first-order fits correspond to Run 2 above.

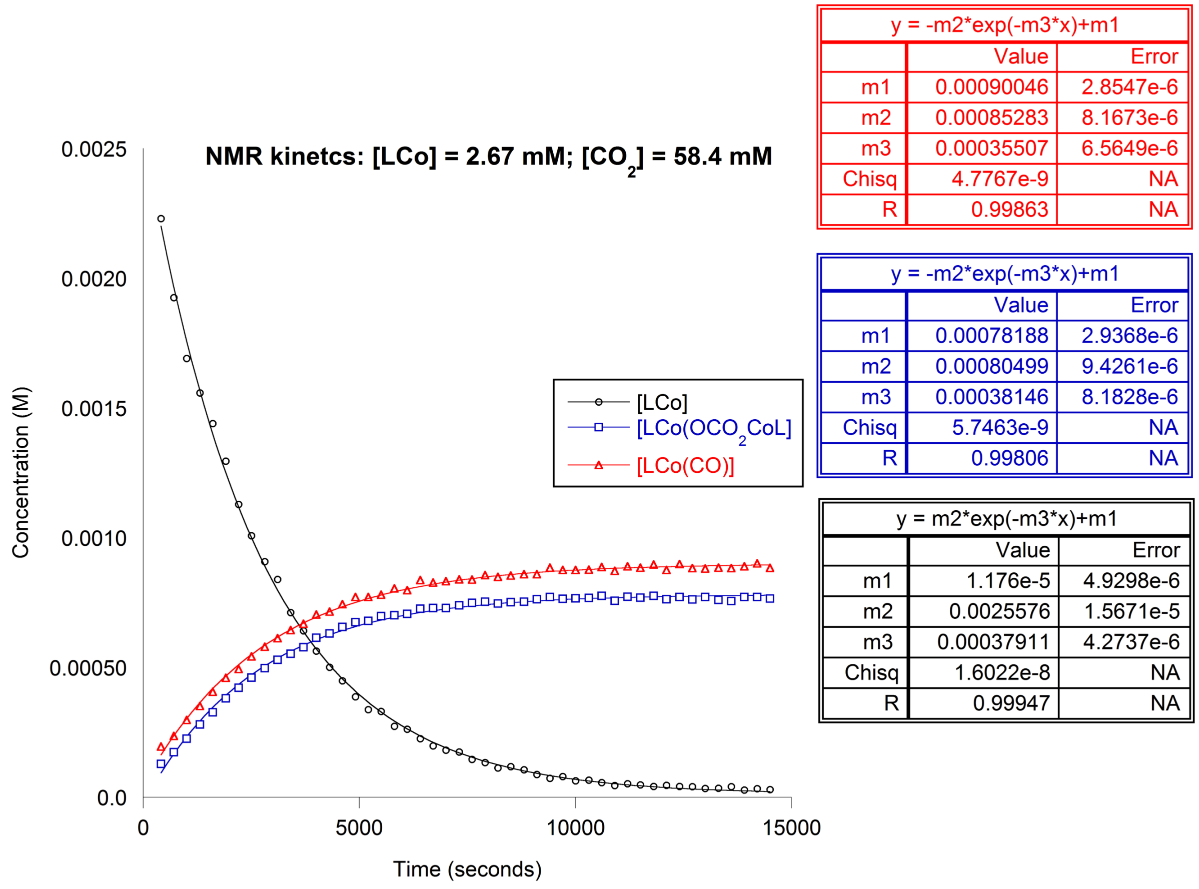


| y = -m2*exp(-m3*x)+m1 | | |
|---|---|---|
| | Value | Error |
| m1 | 0.00090046 | 2.8547e-6 |
| m2 | 0.00085283 | 8.1673e-6 |
| m3 | 0.00035507 | 6.5649e-6 |
| Chisq | 4.7767e-9 | NA |
| R | 0.99863 | NA |

| y = -m2*exp(-m3*x)+m1 | | |
|---|---|---|
| | Value | Error |
| m1 | 0.00078188 | 2.9368e-6 |
| m2 | 0.00080499 | 9.4261e-6 |
| m3 | 0.00038146 | 8.1828e-6 |
| Chisq | 5.7463e-9 | NA |
| R | 0.99806 | NA |

| y = m2*exp(-m3*x)+m1 | | |
|---|---|---|
| | Value | Error |
| m1 | 1.176e-5 | 4.9298e-6 |
| m2 | 0.0025576 | 1.5671e-5 |
| m3 | 0.00037911 | 4.2737e-6 |
| Chisq | 1.6022e-8 | NA |
| R | 0.99947 | NA |

**Figure S8.** Reactant and product concentration over time as determined by NMR spectroscopy in $C_6D_{12}$ at 10 °C; data and first-order fits correspond to Run 3 above.

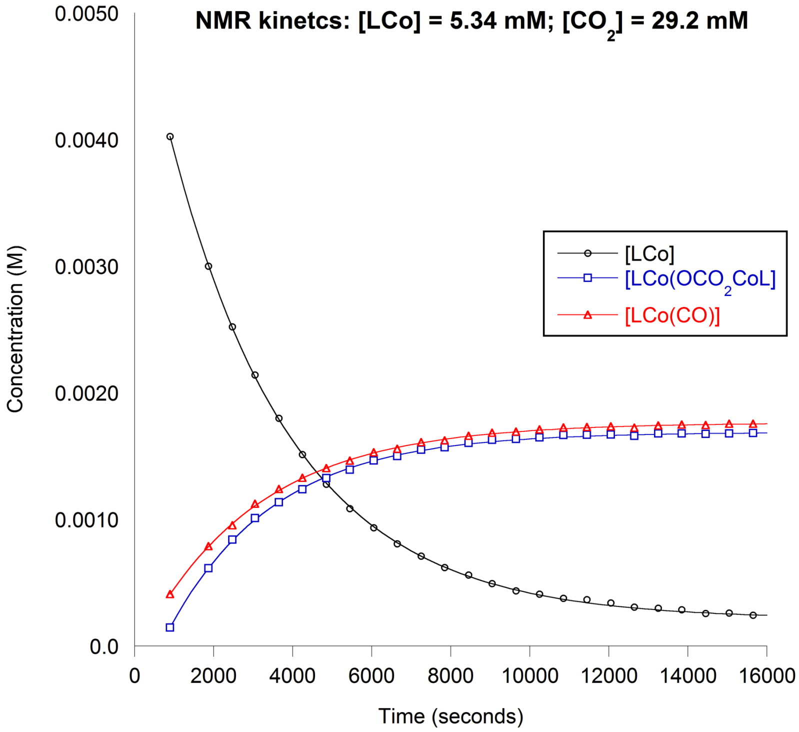


| y = m2*exp(-m3*x)+m1 | | |
|---|---|---|
| | Value | Error |
| m1 | 0.00021326 | 2.6787e-6 |
| m2 | 0.0050861 | 1.6162e-5 |
| m3 | 0.00032073 | 1.2914e-6 |
| Chisq | 7.4237e-9 | NA |
| R | 0.99988 | NA |

| y = -m2*exp(-m3*x)+m1 | | |
|---|---|---|
| | Value | Error |
| m1 | 0.0017634 | 1.4222e-6 |
| m2 | 0.001834 | 9.3184e-6 |
| m3 | 0.00033761 | 2.1087e-6 |
| Chisq | 2.2643e-9 | NA |
| R | 0.9997 | NA |

| y = -m2*exp(-m3*x)+m1 | | |
|---|---|---|
| | Value | Error |
| m1 | 0.0016877 | 1.8803e-6 |
| m2 | 0.0021566 | 1.4427e-5 |
| m3 | 0.00037393 | 2.9169e-6 |
| Chisq | 4.5503e-9 | NA |
| R | 0.99951 | NA |

## D. Attempted synthesis of CO coordinated oxo complex 11

**General procedure for CO gas addition to complex 3:** Solid **3** (2.7 mg, 0.0023 mmol) was dissolved in 0.6 mL benzene-*d*$_6$ and transferred to a J. Young NMR tube containing a nickelocene capillary used as an integration standard. On the Schlenk line, the NMR sample solution was frozen using a liquid nitrogen cold bath. The headspace above the frozen solution was evacuated. CO (7 mbar, 12.55 mL, 0.0035 mmol) was condensed on the frozen solution for 20 min and then the NMR sample was sealed and thawed to ambient temperature with vigorous shaking. Proton NMR indicated formation of the known cobalt bis(carbonyl) complex $L^{tBu}Co(CO)_2$[3] in 34% spectroscopic yield in addition to unidentified minor species.

**Figure S9.** Proton NMR spectra of reactions between **3** and CO.

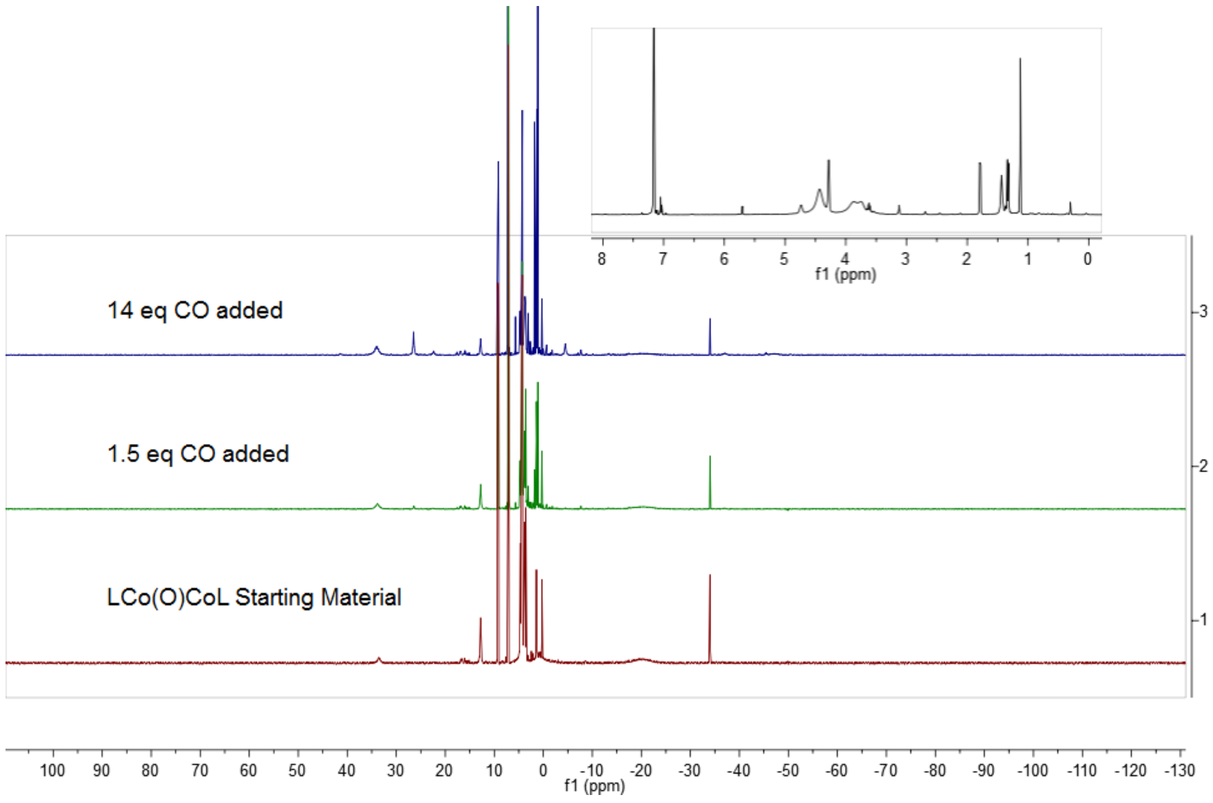

## E. X-ray absorbance spectroscopy of $L^{tBu}Co(O)CoL^{tBu}$ (3) and reference compounds

High Energy Resolution Fluorescence Detection X-ray Absorption Spectroscopy (HERFD-XAS) data were collected at the Cornell High Energy Synchrotron Source at C-line end station. The incident X-rays were monochromated using a Si(220) crystal monochromator. Emitted X-rays were analyzed using a set of three spherically bent Si crystals (531 reflection) mounted on a Rowland circle in combination with a Pilatus Area Detector as previously described.[33, 34] He-filled plastic bags were used to displace air from the beam flight path to minimize attenuation of the fluorescence. The Co HERFD-XAS spectra were collected by monitoring the intensity of the $K\alpha_1$ peak as a function of incident energy. Data were collected from 7650 to 7950 eV. A step size and count time of 10 eV (1 sec), 0.3 eV (5 sec), and 2 eV (2 sec) was used from 7650 to 7690 eV, 7690 to 7750 eV and 7750 to 7950 eV respectively. Data were collected at *ca.* 20 K in a Displex cryostat to minimize photoreduction and to maintain the samples in an inert atmosphere. The data were normalized using PyMCA and six experimental spectra were averaged for each compound. For normalization, the postedge region (E > 7750 eV) was set to an absorbance of 1.0. The first inflection point of Co foil was used as a calibrant, adjusting the energy axis to the reported literature value of 7709.5 eV. All samples were prepared in a nitrogen filled glovebox as 5% Co (wt/wt) solid dilutions in BN.

**Figure S10.** Cobalt K-edge X-ray absorbance spectra of $L^{tBu}Co(O)CoL^{tBu}$ (**3**) and cobalt(II) (LCoCl, three-coordinate; $[LCo(OH)]_2$, four-coordinate) and cobalt(III) ($[LCo(O)]_2$, four-coordinate) reference compounds. The method of preparation for the last two compounds was recently described.[35]

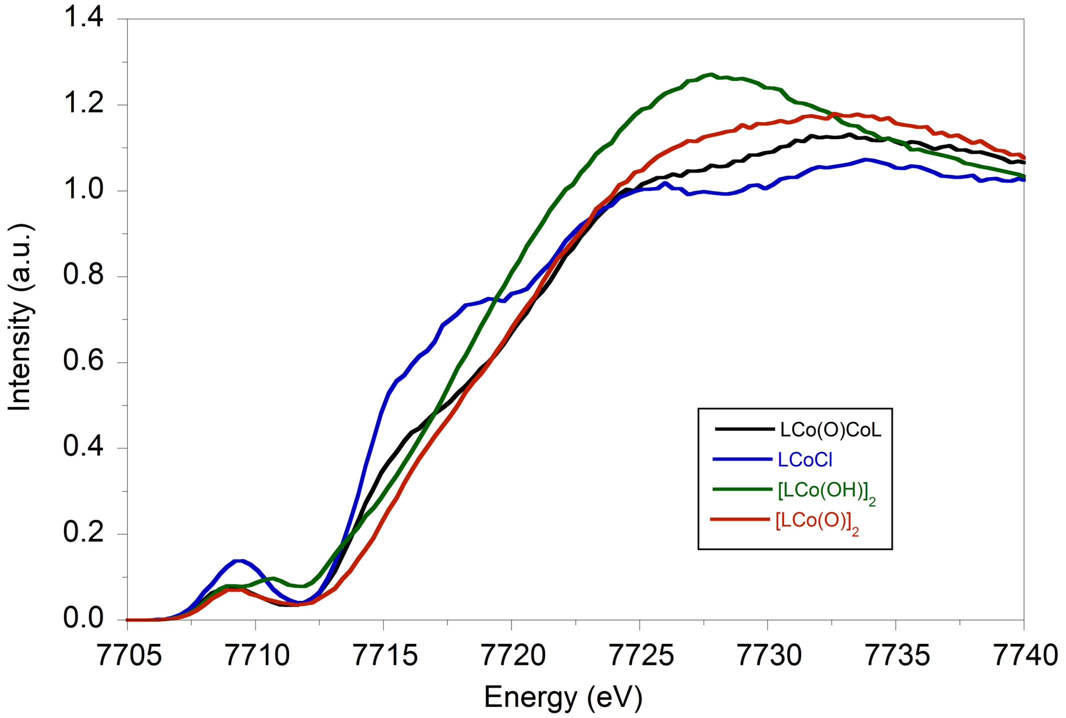

## F. X-ray crystallography

Crystals were placed onto the tip of a 0.1 mm diameter glass capillary tube or fiber and mounted on a Bruker SMART APEX II CCD Platform diffractometer for a data collection at 100.0(5) K.[1] The full data collection was carried out using MoKα radiation (graphite monochromator). A randomly oriented region of reciprocal space was surveyed: six major sections of frames were collected with 0.50° steps in ω at six different ϕ settings and a detector position of -38° in 2θ. The intensity data were corrected for absorption.[2] Final cell constants were calculated from the xyz centroids of >3800 strong reflections from the actual data collection after integration.[3]

Structure (**3**) was solved using SIR97[4] and refined using SHELXL-2012.[5] The space group P-1 was determined based on intensity statistics. A direct-methods solution was calculated which provided most non-hydrogen atoms from the E-map. Full-matrix least squares / difference Fourier cycles were performed which located the remaining non-hydrogen atoms. All non-hydrogen atoms were refined with anisotropic displacement parameters. All hydrogen atoms were placed in ideal positions and refined as riding atoms with relative isotropic displacement parameters. The structure suffered both from non-merohedral twinning and highly disordered solvent. After the non-merohedral twin law, [ -0.510 0.497 -0.020 / 1.470 0.490 -0.061 / -0.490 -0.497 -0.980 ], a 180 degree rotation about reciprocal lattice [ -1 -3 1 ], was determined,[6] the data were re-integrated,[3] and a new absorption correction was applied. There were 21623 unique reflections solely in the first component, 21604 unique reflections solely in the second component, and 4867 unique overlapping reflections. The mass ratio of the two components refined to 60:40. However, since the structure also contained highly disordered solvent (toluene and perhaps hexane), the overlapping reflection contributions from the minor mass component of the twin were removed.[7] Once the data were modified for twinning, they were further modified to remove reflection contributions from the disordered solvent.[8] There were 4 sites of ~200 $Å^3$ each in which around 75 electrons were removed, for a total of 297 electrons in 829 $Å^3$ removed per unit cell. Since the exact amount and identity of solvent were unknown, no solvent was included in the atom list or molecular formula.

Structure (**4**) was solved using SIR97[4] and refined using SHELXL-97.[9] The space group Cc was determined based on systematic absences and intensity statistics. A direct-methods solution was calculated which provided most non-hydrogen atoms from the E-map. Full-matrix least squares / difference Fourier cycles were performed which located the remaining non-hydrogen atoms. All non-hydrogen atoms were refined with anisotropic displacement parameters. All hydrogen atoms were placed in ideal positions and refined as riding atoms with relative isotropic displacement parameters. The structure was refined as an inversion twin, with a final twin occupancy ratio of 87:13.

**Table S1.** Crystallography of **3** and **4**

| **Complex** | **3** | **4** |
|---|---|---|
| **Empirical formula** | $C_{70}H_{106}Co_2N_4O$ | $C_{71}H_{106}Co_2N_4O_3$ |
| **FW** | 1137.44 | 1181.46 |
| **Crystal system** | Triclinic | Monoclinic |
| **Space group** | P-1 | Cc |
| **a (Å)** | 13.475(2) | 20.919(2) |
| **b (Å)** | 21.712(2) | 28.234(3) |
| **c (Å)** | 25.061(3) | 24.420(3) |
| **α (deg)** | 95.195(3) | 90 |
| **β (deg)** | 102.214(2) | 114.237(2) |
| **γ (deg)** | 97.097(3) | 90 |
| **V (Å$^3$)** | 7060(1) | 13152(3) |
| **Z** | 4 | 8 |
| **ρ (g/cm$^3$)** | 1.070 | 1.193 |
| **μ (mm$^{-1}$)** | 0.510 | 0.552 |
| ***R*1, *wR*2 (I > 2σ(I))** | 0.0745, 0.1714 | 0.0603, 0.1053 |
| ***R*1, *wR*2 (all data)** | 0.1348, 0.1960 | 0.1182, 0.1260 |
| **GOF** | 0.918 | 0.973 |

## Crystallographic References


1. *APEX2*, version 2012.4-3; Bruker AXS: Madison, WI, 2012.
2. Sheldrick, G. M. *SADABS*, version 2008/1; University of Göttingen: Göttingen, Germany, 2008.
3. *SAINT*, version 7.68A; Bruker AXS: Madison, WI, 2009.
4. Altomare, A.; Burla, M. C.; Camalli, M.; Cascarano, G. L.; Giacovazzo, C.; Guagliardi, A.; Moliterni, A. G. G.; Polidori, G.; Spagna, R. *SIR97: A new program for solving and refining crystal structures*; Istituto di Cristallografia, CNR: Bari, Italy, 1999.
5. Sheldrick, G. M. *SHELXL-2012* University of Göttingen: Göttingen, Germany, 2012.
6. a) Parsons, S.; Gould, B.; Cooper, R.; Farrugia, L. *ROTAX*; University of Edinburgh: Edinburgh, Scotland, 2003; b) Sheldrick, G. M. *CELL_NOW: A program that analyzes a list of reflections to find a cell and orientation matrix despite the presence of several twin domains or other junk*, version 2008/2; University of Göttingen: Göttingen, Germany, 2008.
7. Sheldrick, G. M. *TWINABS*, version 2008/4; University of Göttingen: Göttingen, Germany, 2008.
8. Spek, A. L. *PLATON: A multipurpose crystallographic tool*, version 300106; Utrecht University: Utrecht, The Netherlands, 2006.
9. Sheldrick, G. M. *Acta. Cryst.* **2008**, *64*, 112-122.

## G. Justification for truncating the computational ligand model

**Figure S11.** Overlay of the optimized geometry of $L^{tBu}Co$ and L'Co with the crystal structure calculated at BP86/B1 with D3BJ correction (see below for basis set information).

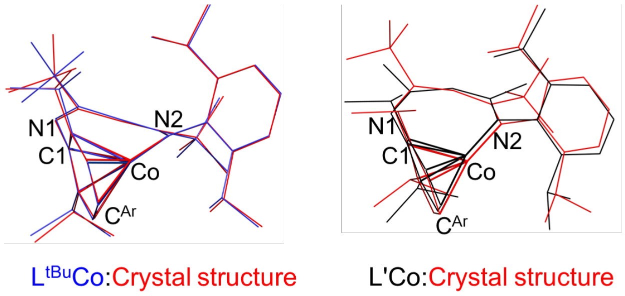


**Table S2.** Crystallographically obtained and DFT calculated interatomic distances (Å) and angles (°) for $L^{tBu}Co$ and L'Co. Deviations from crystal structure are shown by Δ and written in parenthesis.

| | **Crystal Structure** | **$L^{tBu}Co$ (Δ)** | **L'Co (Δ)** |
|---|---|---|---|
| Co-N1 | 3.16 | 3.16 (0.0) | 3.19 (0.03) |
| Co-C1 | 2.09 | 2.08 (-0.01) | 2.09 (0.0) |
| Co-$C^{Ar}$ | 2.15 | 2.16 (0.01) | 2.12 (-0.03) |
| Co-N1-C1 | 30.8 | 30.3 (-0.5) | 29.0 (-1.8) |
| Co-N2 | 1.90 | 1.88 (-0.02) | 1.87 (-0.03) |

Comparing the geometrical parameters of $L^{tBu}Co$ and L'Co (Table S2) obtained at BP86(D3BJ)/B1 level of theory (B1 basis set includes TZVP basis employed on Co and N and def2-SVP basis on C and H atoms) and an overlay of their geometries with the actual crystal structure (Figure S11) show quite good agreement among all the three structures. The bond lengths and angles of the optimized geometries of the model complexes are consistent with the X-ray crystal structure with insignificant deviations (shown in Table S2). This also validates the reliability of the method chosen for optimization. Moreover, the κ-*N*,$\eta^6$-arene ligation is well reproduced in the initial geometry of the complex, i.e. L'Co. The $^{t}$Bu substituents on the crystal structure and the methyl replacers in L'Co are almost collinear. This justifies the validity of the truncated model L'Co to be used in place of the actual $L^{tBu}Co$ framework. However, whenever necessary, the original $L^{tBu}Co$ framework has been used for calculations; for example in comparing the calculated and experimental structure for dinuclear carbonate (shown in Table 1, main text) and also for μ-oxo dicobalt species (shown in Table 2, main text).

## H. Benchmarking the various density functional methods with DLPNO-CCSD(T).

It is observed from Figure S12 that single point energy calculations on optimized geometries (optimized at BP86(D3BJ)/B1) along the reaction coordinate leading to transformation of **1** to **1'** followed by $CO_2$ addition shows variations in the relative barriers depending on the choice of wavefunction based method and density functional. In particular, it is found that B3LYP with and without empirical dispersion correction deviates from the DLPNO-CCSD(T) predicted relative free energies. B3LYP functional predicted that the C-O bond cleavage in the dinuclear dissociative pathway via **TS4** is the rate determining step. However, it would indicate a buildup of the dinuclear carboxylate bridging intermediate **10**, in contrast to the observed reaction. Repeated experimental attempts could not observe the $\kappa^2 N,N'$ isomer (**1′**) of the starting material **1** which indicates that the RDS should be the ligand isomerization. Also kinetics studies showed that the rate does not depend on the concentration of $CO_2$ (as has been discussed in section C of this supporting information), which further emphasized on the experimental identification of only the $\kappa N,\eta^6$-arene isomer. B3LYP on the other hand underestimated the metal-aryl bonding and predicted the $\kappa^2$N,N' isomer to be lower in energy by -10 kcal/mol than the $\kappa$N,$\eta^6$-arene isomer, in disagreement to the experiments (see Figure S12). Interestingly, M06L/def-TZVPP delivers a similar barrier for **TS1** as DLPNO-CCSD(T)/def2-TZVPP. This observation suggests that the B3LYP functional even with the non-covalent corrections cannot properly describe the metal aryl π-bonding in $\kappa$-$N$,$\eta^6$-arene ligated L'Co, whereas M06L is designed to cover the weak interactions. Hence based on this observation, we predict all the important barriers and reaction free energies with DLPNO-CCSD(T)/def2-TZVPP and M06L/def2-TZVPP in the main text.

**Figure S12.** Gibbs free energy (ΔG) profile for isomerization of **1** (L'Co) to **1'** followed by $CO_2$ activation at different levels of theory.

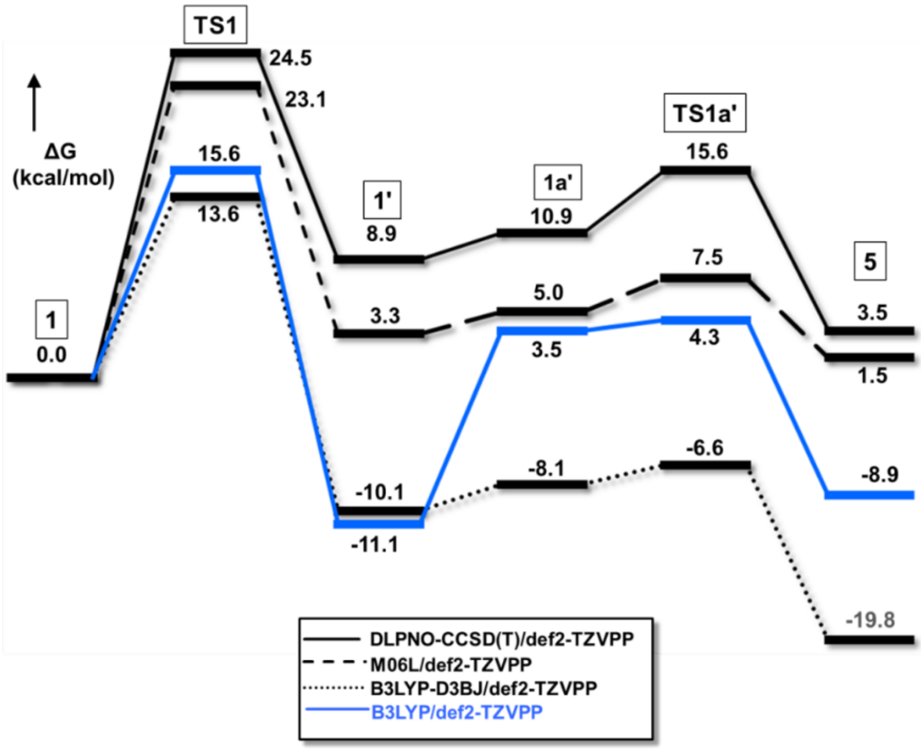

## I. Different bridging binding modes of $CO_2$ with two metal complexes and their geometrical parameters.

**Figure S13.** The two different binding modes of bridging $CO_2$ possible with two metal complexes.

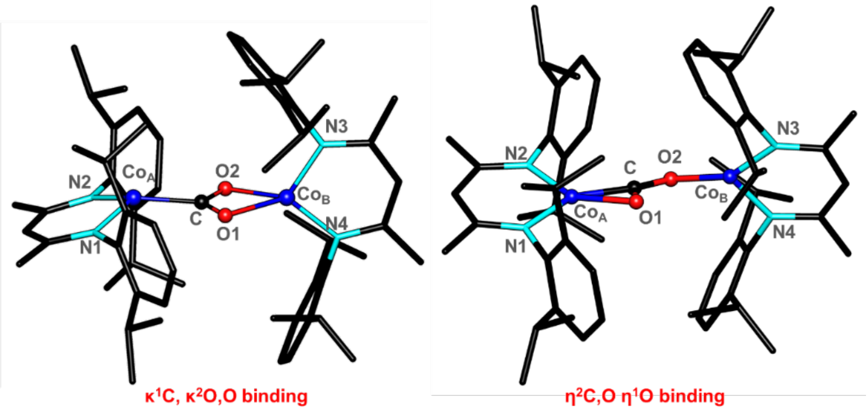


**Table S3.** Binding parameters and geometries of $CO_2$ bridged adducts. Distances in Å and angles in degrees.

| $\eta^1$C, $\eta^2$O,O binding | $Co_A$-N1 | $Co_A$-N2 | $Co_A$-C | C-O1 | C-O2 | O1-C-O2 | $Co_B$-O1 | $Co_B$-O2 | $Co_B$-N3 | $Co_B$-N4 |
|---|---|---|---|---|---|---|---|---|---|---|
| $^{1}\mathbf{10}_{BS33}$ | 1.89 | 1.90 | 1.92 | 1.29 | 1.29 | 117.4 | 2.06 | 2.05 | 1.88 | 1.89 |
| $^{3}\mathbf{10}_{BS31}$ | 1.89 | 1.89 | 1.93 | 1.31 | 1.29 | 113.0 | 1.97 | 1.99 | 1.82 | 1.82 |
| $^{5}\mathbf{10}_{31}$ | 1.88 | 1.90 | 1.94 | 1.29 | 1.30 | 113.1 | 1.99 | 1.97 | 1.82 | 1.82 |
| $^{7}\mathbf{10}$ | 1.90 | 1.90 | 1.93 | 1.30 | 1.30 | 116.7 | 2.06 | 2.06 | 1.89 | 1.90 |
| $\eta^1$C, $\eta^2$O,O binding | | | | | | | | | | |
| $^{1}\mathbf{10}_{BS11}$ | 1.88 | 1.83 | 1.81 | 1.30 | 1.28 | 125.0 | 2.84 | 1.91 | 1.78 | 1.80 |
| $^{3}\mathbf{10}$ | 1.88 | 1.83 | 1.81 | 1.30 | 1.28 | 125.2 | 2.86 | 1.91 | 1.78 | 1.80 |
| $^{3}\mathbf{10}_{BS13}$ | 1.88 | 1.83 | 1.81 | 1.33 | 1.27 | 119.0 | 2.14 | 2.05 | 1.89 | 1.89 |
| $^{5}\mathbf{10}_{13}$ | 1.88 | 1.83 | 1.81 | 1.33 | 1.27 | 119.2 | 2.14 | 2.03 | 1.89 | 1.89 |

The $\kappa^1$C, $\kappa^2$O,nding mode for the S=3 and S=0 (BS33) has been discussed in the main text.

## J. Magneto-structural isomers of intermediate 10

**Figure S14.** The possible electronic structures of intermediate **10**. The overlap between non-orthogonal magnetic orbitals of broken symmetry UHF solution has been marked with the notation "S". The subscript represents the number of unpaired electrons locally on a center.

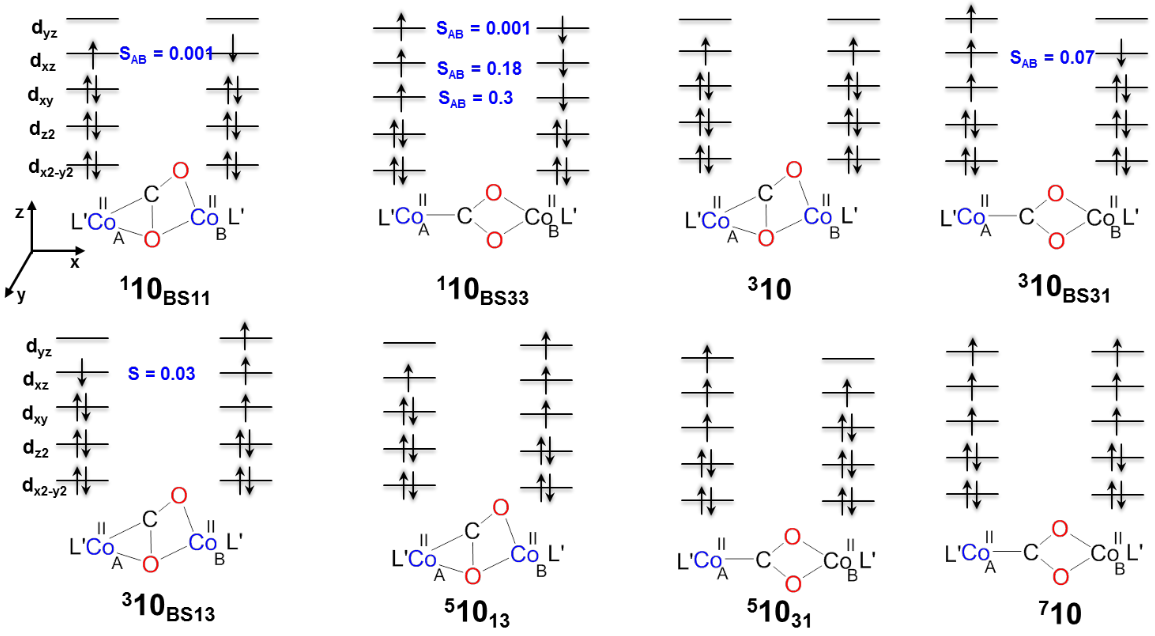


**Table S4.** Mulliken atomic spin population, total spin and free energy values as collected from B3LYP(D3BJ)/def2-TZVPP/B3LYP(D3BJ)/B1 calculations.

| *Intermediate* | *ϱCo$_A$* | *ϱCo$_B$* | S | *ΔG (kcal mol$^{-1}$)* |
|---|---|---|---|---|
| $^{1}$**10**$_{BS11}$ | 1.05 | -1.03 | 0 | 19.1 |
| $^{1}$**10**$_{BS33}$ | 2.60 | -2.62 | 0 | 0.8 |
| $^{3}$**10** | 1.06 | 1.02 | 1 | 18.6 |
| $^{3}$**10**$_{BS31}$ | 2.60 | -1.0 | 1 | 7.4 |
| $^{3}$**10**$_{BS13}$ | -1.10 | 2.66 | 1 | 3.3 |
| $^{5}$**10**$_{13}$ | 1.13 | 2.65 | 2 | 3.3 |
| $^{5}$**10**$_{31}$ | 2.59 | 1.04 | 2 | 9.1 |
| $^{7}$**10** | 2.63 | 2.67 | 3 | 0.0 |

From Figure SI4 and Table S4 we find that the high spin septet intermediate with S=3/2 on both the cobalt (II) centers ($^{7}$**10**) possesses the same energy as the broken symmetry singlet, $^{1}$**10**$_{BS33}$. Also, the ferromagnetically coupled quintet $^{5}$**10**$_{13}$ and its corresponding antiferromagnetic triplet counterpart $^{3}$**10**$_{BS13}$ have slightly higher energies than the high spin states. Hence, to reduce computational effort, we have chosen the lowest energy septet and the broken symmetry singlet to explore the reaction mechanism. It is to be noted that geometries of the chosen intermediate on these two states are also similar.

## K. B3LYP calculated free energy profiles

**Figure S15.** B3LYP/def2-TZVPP Gibbs free energy (ΔG) profile for mononuclear (left) and dinuclear (right) dissociative pathway

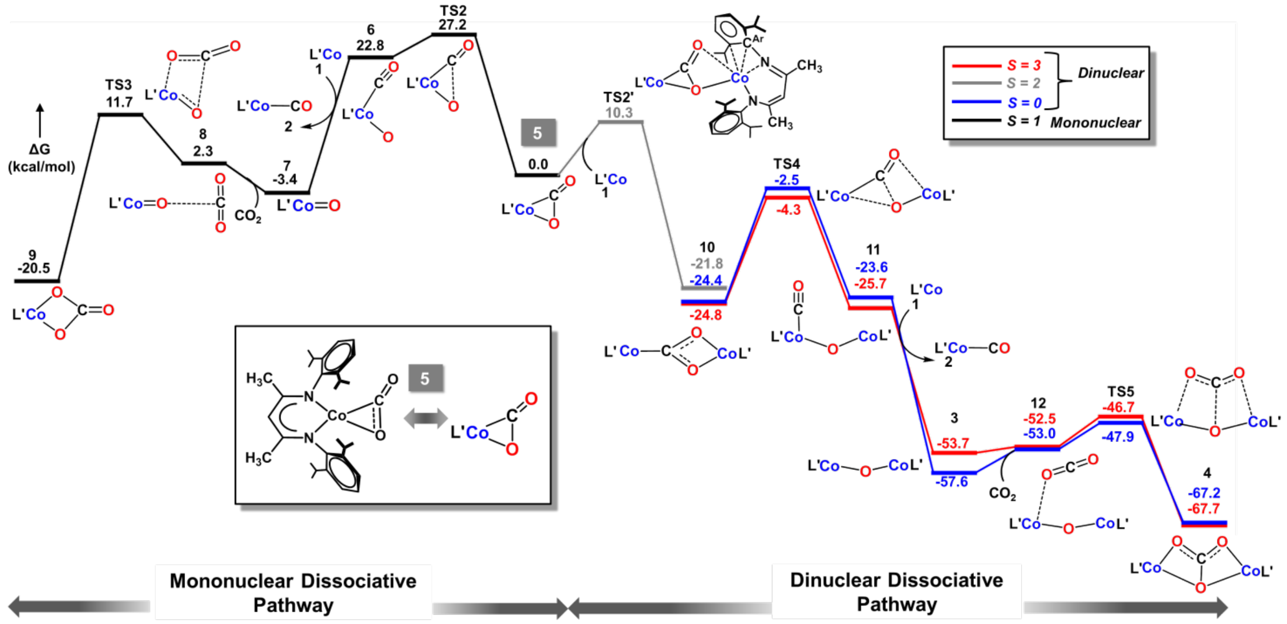


**Figure S16.** B3LYP/def2-TZVPP Gibbs free energy (ΔG) profile for dinuclear associative pathway.

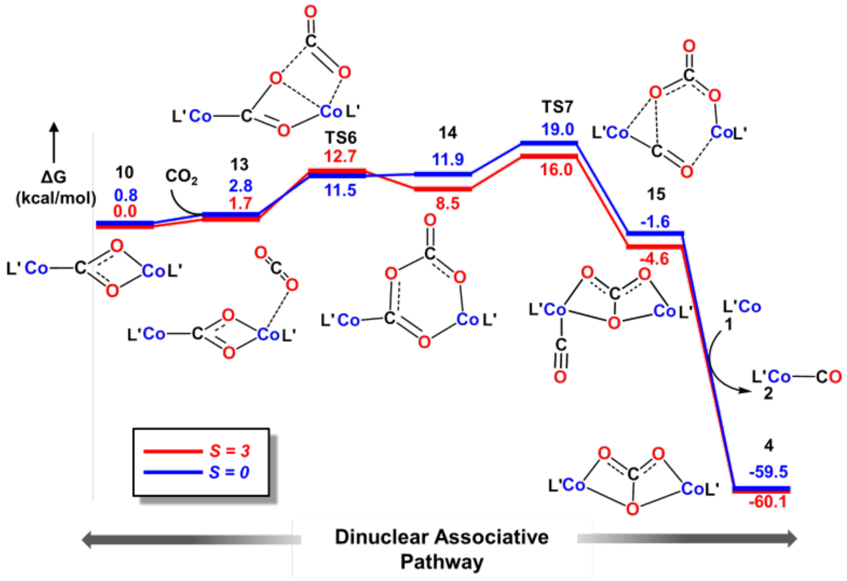

## L. M06L calculated free energy profiles

**Figure S17.** M06L/def2-TZVPP Gibbs free energy (ΔG) profile for mononuclear and dinuclear dissociative and dinuclear associative pathways.

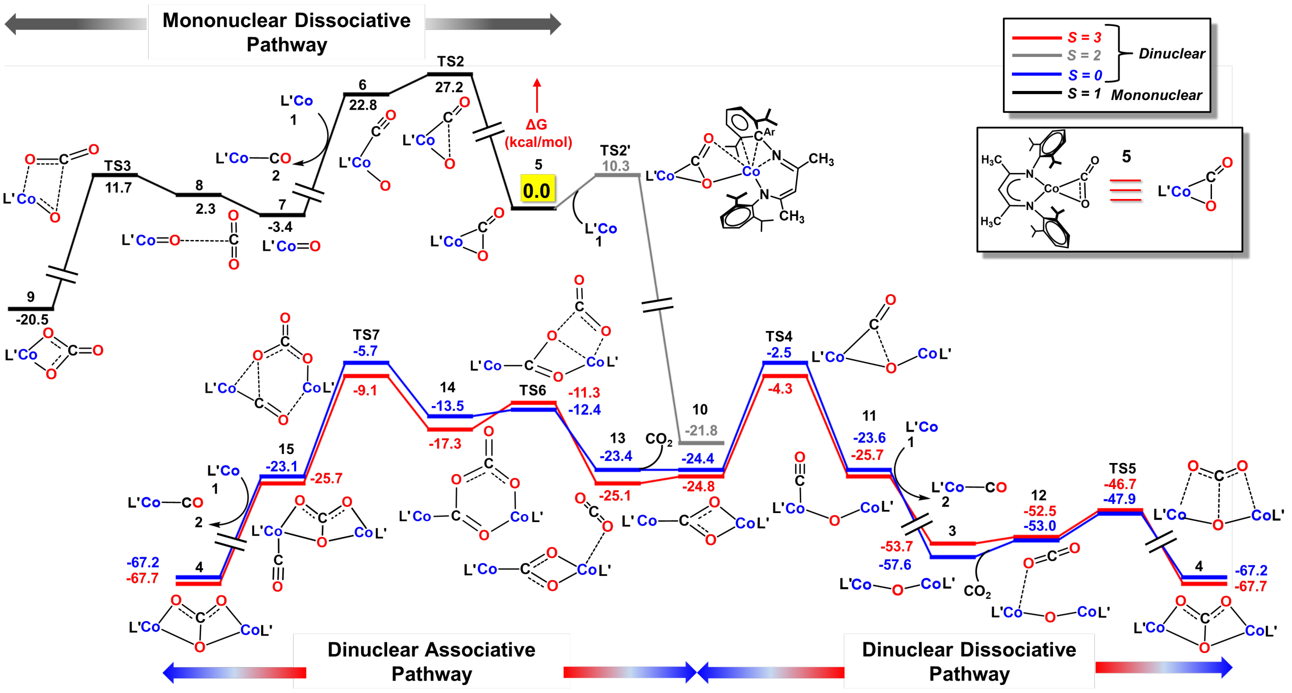

## M. Optimized geometry of important intermediates and transition states with BP86/B1

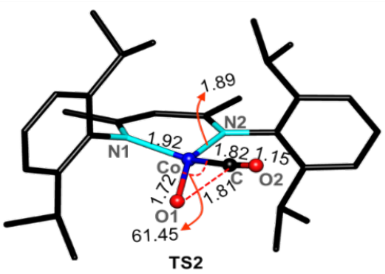


TS2

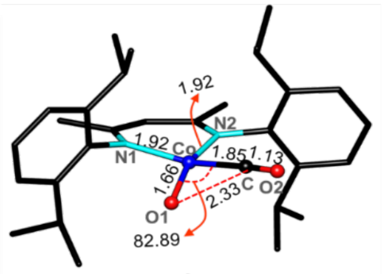


6

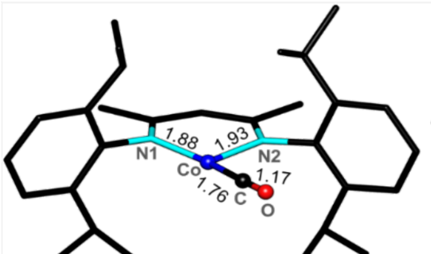


2

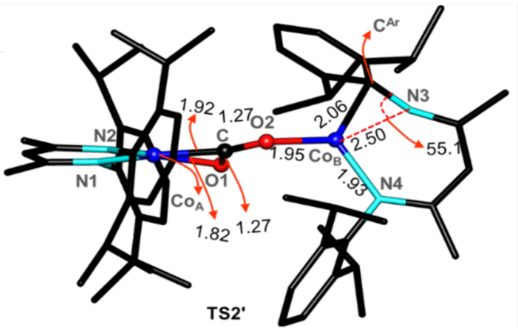


TS2'

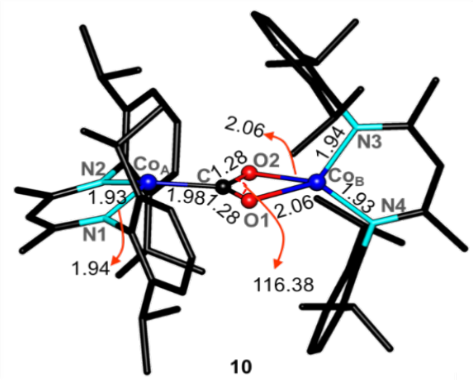


10

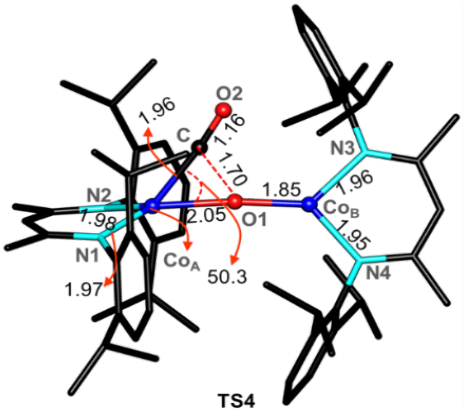


TS4

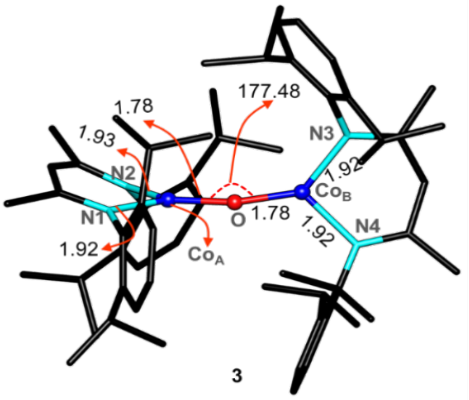


3

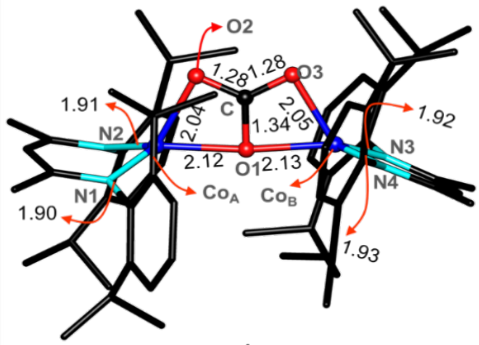


4

## N. XYZ coordinates of optimized geometries.

### $(L^{tBu}Co)_2\mu\text{-}CO_3$

| | | | |
|---|---|---|---|
| Co | 7.13217946770792 | 26.13376195867381 | 17.80263950116836 |
| Co | 9.99228411947636 | 22.70740819360404 | 17.81099296671146 |
| C | 7.79613357253401 | 23.77789028777456 | 18.18569685668649 |
| O | 6.63255194663147 | 24.29161534497207 | 18.36736510878959 |
| O | 8.75122954680276 | 24.56552223585289 | 17.72036234734129 |
| O | 8.09615756722283 | 22.55306062601858 | 18.42345691879625 |
| N | 6.82147517750987 | 27.58974709430260 | 19.03316992701278 |
| N | 6.51147324784066 | 26.90810352819571 | 16.14662318319071 |
| C | 4.91546147990525 | 29.14358748260706 | 19.83687196237873 |
| C | 5.75560022799669 | 28.37314062726186 | 18.75469583016602 |
| C | 5.21137265900650 | 28.44461420378086 | 17.45339207193712 |
| H | 4.35843895847155 | 29.11349082416984 | 17.38262333586399 |
| C | 5.59232290587258 | 27.88617688905426 | 16.20335057295230 |
| C | 4.87575558310163 | 28.56804422154542 | 14.98411154727617 |
| C | 5.56274869533388 | 29.36978320275432 | 21.22050536498660 |
| H | 4.80068234731106 | 29.83227144257115 | 21.87425225433752 |
| H | 6.42652297981954 | 30.05149180676707 | 21.17994021803052 |
| H | 5.89375383658315 | 28.44014346554600 | 21.70194549280776 |
| C | 3.65647605226582 | 28.25843255650254 | 20.05537047301396 |
| H | 2.99319819583949 | 28.72231256027773 | 20.80879792409150 |
| H | 3.94437003148302 | 27.25648035307819 | 20.42033339748997 |
| H | 3.08491266524602 | 28.12632193618841 | 19.12074156632291 |
| C | 4.48299872042992 | 30.54466527435017 | 19.33233680084662 |
| H | 3.95801006384418 | 31.07656109822108 | 20.14578798802812 |
| H | 3.79225450935474 | 30.51158802618719 | 18.47358480287881 |
| H | 5.35928869884159 | 31.15244510419229 | 19.04332174069608 |
| C | 5.10362369450934 | 27.96884929045826 | 13.57873486220450 |
| H | 4.48475619826235 | 28.54454834177291 | 12.86643283216809 |
| H | 4.80241778085780 | 26.91315429160306 | 13.50592437394724 |
| H | 6.14690570887833 | 28.04112107713364 | 13.24325055439005 |
| C | 5.37296971201795 | 30.03863157104664 | 14.94243642956246 |
| H | 4.89141682735863 | 30.57437519408461 | 14.10353343782842 |
| H | 6.46590838255760 | 30.07860367506211 | 14.78994805087569 |
| H | 5.14354248496805 | 30.57834323602059 | 15.87654716190528 |
| C | 3.33847208709145 | 28.55247609262632 | 15.21311244407288 |
| H | 2.83563198236111 | 28.97923576683193 | 14.32685176147011 |
| H | 3.01831042931997 | 29.14536053816269 | 16.08531405230702 |
| H | 2.96699486046171 | 27.52199748875039 | 15.35086025788610 |
| C | 7.69713552209739 | 27.81785307970974 | 20.13575054669482 |
| C | 8.47448013164333 | 29.01702410012469 | 20.15112751954936 |
| C | 9.36843208804165 | 29.21977175186198 | 21.21647394755788 |
| H | 9.96998789345154 | 30.13529619145467 | 21.24647350595522 |
| C | 9.49574974517700 | 28.28001758046542 | 22.25161084966268 |
| H | 10.1932683924476 | 28.46233422747464 | 23.07864475123201 |
| C | 8.72230433175950 | 27.11395720720569 | 22.22348079911131 |
| H | 8.81265135236811 | 26.38166996503741 | 23.03430676717636 |

### $L^{tBu}CoOCoL^{tBu}$

| | | | |
|---|---|---|---|
| Co | -3.13377753809389 | 3.07378229033125 | 17.37966214852013 |
| Co | -5.45924574779238 | 4.23624686024651 | 19.58857466310224 |
| O | -4.18413216818778 | 3.83921027757140 | 18.50491384320510 |
| N | -6.34690670507176 | 3.31758851032229 | 20.98777503472765 |
| N | -6.40361939722532 | 5.89250984026087 | 19.55188773468333 |
| C | -8.67817357511030 | 2.57127624485501 | 21.77392277120834 |
| C | -7.66470566527026 | 3.60422648688880 | 21.16866051691908 |
| C | -8.21850082707133 | 4.80483388166122 | 20.68283901894907 |
| H | -9.27729779331618 | 4.92345358953812 | 20.89671863508452 |
| C | -7.63139607679846 | 5.95883327588207 | 20.08161541448252 |
| C | -8.13994607214257 | 1.66637308678147 | 22.90808653492655 |
| H | -8.97113170351857 | 1.02853796298722 | 23.26055194993374 |
| H | -7.77340038397527 | 2.24883173147741 | 23.76833919732611 |
| H | -7.32694885310256 | 1.00337030043680 | 22.58474846841851 |
| C | -9.09928411031228 | 1.67130442066948 | 20.58322284661856 |
| H | -9.82683152909637 | 0.90820436342624 | 20.91737014921023 |
| H | -8.22764068439481 | 1.14997131382487 | 20.15611396015153 |
| H | -9.56411503503027 | 2.27103745548794 | 19.78100236461809 |
| C | -9.95193631129882 | 3.25318191109959 | 22.33412574560982 |
| H | -10.5853991069010 | 2.48685695635420 | 22.81491958362260 |
| H | -10.5680490530631 | 3.72889959215718 | 21.55233375883298 |
| H | -9.70857730437158 | 4.01435153162156 | 23.09707029471877 |
| C | -8.53285904355505 | 7.23411739114658 | 20.16417215332896 |
| C | -8.84163877735539 | 7.48384278939212 | 21.66587148306774 |
| H | -7.90615290038491 | 7.65598617551916 | 22.22832125317904 |
| H | -9.36421560170676 | 6.63565657224749 | 22.13729721896004 |
| H | -9.47628829811215 | 8.38287311643052 | 21.77267671312661 |
| C | -9.84935214461311 | 6.96037006491053 | 19.39135590852787 |
| H | -10.4958456454772 | 7.85618923284700 | 19.42999964043020 |
| H | -10.4230504376642 | 6.11617102172920 | 19.80921619167432 |
| H | -9.64049851617865 | 6.73556912235462 | 18.33081453691703 |
| C | -7.94891269320021 | 8.55370799962815 | 19.61610617789700 |
| H | -7.01962201211103 | 8.84810177677433 | 20.12743946645366 |
| H | -8.69636812183318 | 9.34914000621658 | 19.79221545453676 |
| H | -7.74114149772433 | 8.52145078382554 | 18.53653203127680 |
| C | -5.57320477251995 | 2.51859038487340 | 21.88148516813296 |
| C | -5.18401118506798 | 3.10121518114822 | 23.12594423426817 |
| C | -4.51620948343821 | 2.29519603426906 | 24.06523386173362 |
| H | -4.23589962439878 | 2.72192749192894 | 25.03568764064683 |
| C | -4.18542994253161 | 0.96280021125886 | 23.77821362455983 |
| H | -3.67478328962965 | 0.34534752379285 | 24.52741093002922 |
| C | -4.46665156675809 | 0.44592793322876 | 22.50701191214421 |
| H | -4.15571254711102 | -0.57695962050492 | 22.25880109796970 |
| C | -5.14162259884707 | 1.20935897788284 | 21.53495370700798 |
| C | -5.43738287212315 | 4.57839123091487 | 23.41507778873095 |

| | | | |
|---|---|---|---|
| C | 7.81935027848898 | 26.85677320466372 | 21.17500866728144 |
| C | 8.33314244783555 | 30.05532239653097 | 19.04086066341879 |
| H | 7.26859638796409 | 30.08183867412973 | 18.74519693273754 |
| C | 8.70934113547510 | 31.48241802684345 | 19.46539871476518 |
| H | 8.46293214578350 | 32.19282069591650 | 18.65587776485578 |
| H | 9.79165412309242 | 31.58232683966904 | 19.66833159203374 |
| H | 8.16298343129658 | 31.79629078389917 | 20.37337784297235 |
| C | 9.11618794606461 | 29.62046571071085 | 17.79109933048700 |
| H | 8.81513226255705 | 28.60755167428628 | 17.47909367427708 |
| H | 10.2016188856433 | 29.60458729014735 | 17.99579468473233 |
| H | 8.93053535022665 | 30.31107184897039 | 16.94817565609616 |
| C | 6.95702448358682 | 25.60329192934051 | 21.22783086125597 |
| H | 6.38741605468324 | 25.54601242208778 | 20.28530410864616 |
| C | 5.93411723453686 | 25.66015346976632 | 22.38103394971981 |
| H | 5.25771775353561 | 26.52813940285606 | 22.29227025002732 |
| H | 6.44196981984812 | 25.72847807451174 | 23.36137567353663 |
| H | 5.31205435975718 | 24.74638278173792 | 22.38530712871357 |
| C | 7.81417330993976 | 24.33048271360625 | 21.33433297109642 |
| H | 8.63794045325565 | 24.32502566026299 | 20.60125480292124 |
| H | 7.19833750030841 | 23.43124214859296 | 21.16434067861612 |
| H | 8.28041652882943 | 24.24186890522660 | 22.33078493685506 |
| C | 6.91568141214656 | 26.23534213018939 | 14.95097649041801 |
| C | 6.26673313060117 | 25.02288453533102 | 14.58911037328125 |
| C | 6.61549069985030 | 24.42219736232154 | 13.36472245199333 |
| H | 6.10610584408714 | 23.49982647278946 | 13.06278685166610 |
| C | 7.58763367729051 | 24.98499070573030 | 12.52805995767318 |
| H | 7.82402470921494 | 24.51648970720871 | 11.56483307790440 |
| C | 8.27611394799098 | 26.13552956932843 | 12.93988743518923 |
| H | 9.06822387079347 | 26.54929991629300 | 12.30403213816550 |
| C | 7.96686773081038 | 26.77045593714027 | 14.15573553572195 |
| C | 5.18608266338552 | 24.39008622336481 | 15.45774041725353 |
| H | 5.18776077935502 | 24.91483809138959 | 16.42889574450689 |
| C | 3.78331448248837 | 24.55434086361420 | 14.84059845710428 |
| H | 3.51844859482283 | 25.61618849559573 | 14.70261325707224 |
| H | 3.01780215948342 | 24.09597514182266 | 15.49311529180715 |
| H | 3.72538581838255 | 24.06205462378556 | 13.85164125425279 |
| C | 5.48688331242873 | 22.90612976436779 | 15.73013225870546 |
| H | 6.52681218857544 | 22.74609050312387 | 16.05806928605168 |
| H | 5.33783412447531 | 22.29311554159280 | 14.82427149071034 |
| H | 4.81939760697604 | 22.51510790311879 | 16.51782608913135 |
| C | 8.75212213725577 | 27.99123810959068 | 14.61276622610262 |
| H | 8.16524834886725 | 28.47579326826424 | 15.41241688300476 |
| C | 10.0904108049376 | 27.53941887638371 | 15.23038166421933 |
| H | 9.92651262746945 | 26.80765901057428 | 16.04000629135776 |
| H | 10.6457570082370 | 28.40023007060021 | 15.64379722328598 |
| H | 10.7250067720190 | 27.05607500721567 | 14.46568920989237 |
| C | 8.97846210850299 | 29.03266184253006 | 13.50352100493142 |
| H | 8.02806027589556 | 29.34304788792945 | 13.03369104332761 |
| H | 9.63791445953592 | 28.64576447698517 | 12.70501458911221 |
| H | 9.46313701238922 | 29.93405827722589 | 13.92065280039687 |
| H | -5.48746959495396 | 5.07661288566134 | 22.42779556456626 |
| C | -4.28517884291169 | 5.22010926675743 | 24.20872093420082 |
| H | -4.39929988089835 | 6.31888400081564 | 24.22123092448779 |
| H | -3.30426074885567 | 4.98013048339727 | 23.76370262266293 |
| H | -4.27318343091934 | 4.88191789798778 | 25.26143128123851 |
| C | -6.77610668254082 | 4.84364021108671 | 24.13126682440911 |
| H | -7.63321360403685 | 4.52480608402490 | 23.51864871854727 |
| H | -6.89711438947328 | 5.92390517331208 | 24.33313341487610 |
| H | -6.81790235326008 | 4.30800982683591 | 25.09832488971422 |
| C | -5.37651772035050 | 0.60515467447966 | 20.15803343815897 |
| H | -5.96623395721180 | 1.33518406378478 | 19.57478103259000 |
| C | -4.03460679474421 | 0.39513056796267 | 19.42984863369396 |
| H | -3.52755990575240 | 1.35779016033502 | 19.23741952984501 |
| H | -4.19075465761482 | -0.09712048494780 | 18.45520306633344 |
| H | -3.35407899728309 | -0.24335616237684 | 20.02264691760847 |
| C | -6.16380362743907 | -0.71805668173546 | 20.22970628841541 |
| H | -7.11149622006610 | -0.60535031373589 | 20.78418516838367 |
| H | -5.57498161047033 | -1.50654621458579 | 20.73338804713643 |
| H | -6.40201773355500 | -1.08243073978290 | 19.21409242597067 |
| C | -5.71889104085603 | 6.94483530062091 | 18.86688821742642 |
| C | -5.92747311390356 | 7.12519687609508 | 17.47398031349889 |
| C | -5.27568705426615 | 8.19727466977124 | 16.83532504410346 |
| H | -5.44212985367990 | 8.35935044543201 | 15.76288891986572 |
| C | -4.41630709340024 | 9.05077425434343 | 17.53875284354761 |
| H | -3.92475951338245 | 9.88594308811551 | 17.02463482875087 |
| C | -4.15693016207967 | 8.80538574970853 | 18.89526038933134 |
| H | -3.44944808554755 | 9.44573356036896 | 19.43575199530516 |
| C | -4.79111795722216 | 7.75252384897659 | 19.57791179829199 |
| C | -6.79170684660028 | 6.17689296767842 | 16.65449793445271 |
| H | -7.26602959061856 | 5.46328786153082 | 17.35269443263545 |
| C | -5.89795804706013 | 5.37116849027988 | 15.69418068691960 |
| H | -5.12156544988433 | 4.81576373560752 | 16.24793206452017 |
| H | -5.38509724665892 | 6.04046860498498 | 14.97951682606158 |
| H | -6.48745615935979 | 4.64279366939216 | 15.11335781827874 |
| C | -7.91306965459134 | 6.90250221026513 | 15.88763074458255 |
| H | -7.50107098901582 | 7.59074597185442 | 15.12656979502312 |
| H | -8.55372555108671 | 7.49709608465768 | 16.56293986303853 |
| H | -8.55392144720258 | 6.17188162827497 | 15.36118809747673 |
| C | -4.44006428427745 | 7.44174958697830 | 21.02453701890841 |
| H | -5.26924029205445 | 6.84243640190120 | 21.44457601172105 |
| C | -3.17131129041669 | 6.56673052762708 | 21.03053779711491 |
| H | -3.33883486622336 | 5.62698300044702 | 20.47344221437472 |
| H | -2.86017052236149 | 6.30813970430126 | 22.05692152828397 |
| H | -2.33436606243427 | 7.08884945150916 | 20.53575951610765 |
| C | -4.27895161457244 | 8.68984006350091 | 21.90679156451470 |
| H | -5.17669659622464 | 9.33345444892334 | 21.86792101140156 |
| H | -3.40989136621815 | 9.30158066291087 | 21.60253439308189 |
| H | -4.11549638039988 | 8.39374936090216 | 22.95877875858672 |
| N | -1.23405792675512 | 3.16338550441589 | 17.51109297131588 |
| N | -3.35829741397303 | 1.96663749222432 | 15.86247668889557 |

| | | | |
|---|---|---|---|
| N | 10.4335659648025 | 21.30787724297569 | 16.56598280498759 |
| N | 11.5623124071407 | 22.65085210029934 | 18.89702726721091 |
| C | 12.1077569933478 | 19.63056115528211 | 15.49158036796572 |
| C | 11.6584463850173 | 20.76249296734719 | 16.48604846178227 |
| C | 12.6735356990644 | 21.14007972847553 | 17.39734911307154 |
| H | 13.6271595992948 | 20.64748850174080 | 17.21872162993402 |
| C | 12.6595765774809 | 21.94778884831942 | 18.55685585646970 |
| C | 14.01378826425431 | 21.91084027929540 | 19.35254867395973 |
| C | 11.07722454754147 | 19.13714759336409 | 14.45229408905308 |
| H | 11.53490743834149 | 18.29475863937584 | 13.90179324790961 |
| H | 10.80912113351713 | 19.90791367969914 | 13.71489839895331 |
| H | 10.14477954874661 | 18.77111784963988 | 14.90610070462866 |
| C | 13.34707786240672 | 20.11313236408850 | 14.69191558044857 |
| H | 13.67742749154000 | 19.31036944839835 | 14.00777430127933 |
| H | 14.20098620669393 | 20.37328901038444 | 15.33911221379823 |
| H | 13.10556078769856 | 20.99951742849574 | 14.07888758237657 |
| C | 12.50832291699759 | 18.39657031048826 | 16.34698300711485 |
| H | 12.81326378555521 | 17.56683309576895 | 15.68326603400294 |
| H | 11.65911963196698 | 18.04877507881016 | 16.96049553993850 |
| H | 13.34638654374173 | 18.61091287643348 | 17.03011817371735 |
| C | 15.12173240277976 | 22.45174909978637 | 18.40758069959684 |
| H | 16.09658381498093 | 22.43141700977983 | 18.92833113113500 |
| H | 14.91307565150347 | 23.49598306964293 | 18.11482089624651 |
| H | 15.21971914712421 | 21.85864843972383 | 17.48330017703257 |
| C | 14.11072335588597 | 22.72755660001237 | 20.65996004330512 |
| H | 15.14916310130273 | 22.64184690426346 | 21.02949478290566 |
| H | 13.44378494217826 | 22.35312522932528 | 21.45005484207259 |
| H | 13.89435340050091 | 23.79568980384829 | 20.52084204366540 |
| C | 14.32873107883546 | 20.43910995946370 | 19.73126780047270 |
| H | 15.26677908416003 | 20.39907664279605 | 20.31477301841853 |
| H | 14.45334115105305 | 19.78757599253159 | 18.85078295055227 |
| H | 13.52267271075340 | 20.01319095252084 | 20.35336337352004 |
| C | 9.36420323663318 | 21.09433892454600 | 15.64944283998992 |
| C | 8.27459941972950 | 20.25791174841426 | 16.01693977357808 |
| C | 7.26437095234863 | 20.03631028393933 | 15.06180113876445 |
| H | 6.42836594448901 | 19.37165499839606 | 15.31208799482610 |
| C | 7.30503007147357 | 20.65105839595110 | 13.80362098690038 |
| H | 6.51058050523048 | 20.45928686201384 | 13.07161530456565 |
| C | 8.35055724874366 | 21.53292918605703 | 13.48870181266183 |
| H | 8.36426068227528 | 22.02360717531872 | 12.51040724827154 |
| C | 9.39331213743764 | 21.77668963712902 | 14.39784931420813 |
| C | 8.21728838887680 | 19.56118713258133 | 17.37249325485350 |
| H | 8.95796251391552 | 20.05891367461623 | 18.02465092378643 |
| C | 6.83744441228449 | 19.70292101939463 | 18.03968263202338 |
| H | 6.55184337997864 | 20.76387376075094 | 18.11594942485301 |
| H | 6.86531475629813 | 19.28002547098404 | 19.06041531680621 |
| H | 6.05585762741749 | 19.15884662108500 | 17.47739327495967 |
| C | 8.61304371028460 | 18.07505679508328 | 17.26092992708166 |
| H | 9.62644461805317 | 17.95065397140116 | 16.84444581665030 |
| H | 7.91131414543947 | 17.52771780168404 | 16.60381544722069 |
| C | -2.69250383022620 | -0.31421457759279 | 14.89163956491143 |
| C | -2.39112894026669 | 1.03753353396002 | 15.62587078309344 |
| C | -1.10011952901475 | 1.18048539170975 | 16.16850733432871 |
| H | -0.41164362487681 | 0.38601100318383 | 15.89360457033514 |
| C | -0.48251845943236 | 2.23404924817751 | 16.90898697156933 |
| C | 1.07977284744354 | 2.19759170842833 | 16.86892958582198 |
| C | -3.28230675313664 | -1.24432384760439 | 15.98390279408232 |
| H | -3.51783381952634 | -2.23675143834741 | 15.55629160901576 |
| H | -4.21051443222679 | -0.82132414967739 | 16.40047714002428 |
| H | -2.56734271432159 | -1.38102720334402 | 16.81421591320236 |
| C | -3.69563836780937 | -0.24736902845268 | 13.71485399657455 |
| H | -3.36215091004607 | 0.43739084198160 | 12.91892503324680 |
| H | -4.70231799737643 | 0.06521968867518 | 14.02234590053869 |
| H | -3.77893762101220 | -1.25941930115618 | 13.27807247989326 |
| C | -1.41117247555437 | -0.98217555835960 | 14.33358936865114 |
| H | -0.86481686460844 | -0.31276351666136 | 13.64497433161003 |
| H | -1.69663987689528 | -1.88585860950770 | 13.76652050939149 |
| H | -0.71549189369242 | -1.31006992760701 | 15.12466486502756 |
| C | 1.49520468663032 | 2.30508894777476 | 15.37623142787738 |
| H | 1.08506201319312 | 1.48387367149309 | 14.76581284159687 |
| H | 2.59737709235021 | 2.28436729334883 | 15.29013793069032 |
| H | 1.13531009934256 | 3.25636615225282 | 14.94400634145584 |
| C | 1.56718402029816 | 0.84858470977684 | 17.45964625135410 |
| H | 1.22938920319268 | 0.73448241956870 | 18.50472109909392 |
| H | 2.67207865581364 | 0.81957378249721 | 17.45270305955048 |
| H | 1.20883602840644 | -0.02547292141921 | 16.89079276066968 |
| C | 1.83663843526221 | 3.31485712832474 | 17.61799721902207 |
| H | 1.65588852354529 | 3.30649217018102 | 18.70300251694960 |
| H | 1.58729082502284 | 4.31982407795045 | 17.24565511925551 |
| H | 2.91833094305136 | 3.15450157735045 | 17.45477846973992 |
| C | -4.39640172228455 | 2.28249882455433 | 14.93723397127185 |
| C | -4.03968554238276 | 3.02012833844505 | 13.76802012609627 |
| C | -5.02581793498367 | 3.25122623551321 | 12.79237520475465 |
| H | -4.75851751377980 | 3.79233354687121 | 11.87648187735451 |
| C | -6.34741935928019 | 2.81880084023840 | 12.97600797574082 |
| H | -7.10132213706317 | 2.99697791358241 | 12.19937105844951 |
| C | -6.70577380818360 | 2.20275467968028 | 14.18249396726038 |
| H | -7.75143060949502 | 1.91577126173763 | 14.35268150512192 |
| C | -5.75333835654647 | 1.94067029411574 | 15.18610500104839 |
| C | -2.63483669090446 | 3.59268763559343 | 13.59844483627409 |
| H | -2.20571170614926 | 3.66034144182182 | 14.61618109423502 |
| C | -2.66334738544475 | 5.01402168616909 | 13.00831405254918 |
| H | -1.66098331978617 | 5.47368906545733 | 13.07434291165505 |
| H | -3.37509270555393 | 5.65875485086409 | 13.55161221085119 |
| H | -2.95203749186485 | 5.01090570716322 | 11.94105858974017 |
| C | -1.70178804122881 | 2.69211475056575 | 12.76638274074387 |
| H | -2.11439020260610 | 2.52484882501560 | 11.75361026587358 |
| H | -1.55312965990436 | 1.71242759228358 | 13.24622008746585 |
| H | -0.70747742246906 | 3.16298078035478 | 12.65791220958459 |
| C | -6.21131543407471 | 1.30839057649145 | 16.49232729998469 |

| | | | |
|---|---|---|---|
| H | 8.59417544860859 | 17.59225361931011 | 18.25541427289296 |
| C | 10.56279429892097 | 22.69589155865462 | 14.05651930368936 |
| H | 11.47878378771620 | 22.20173607748261 | 14.42972945137906 |
| C | 10.45491953681399 | 24.04228133295679 | 14.79438136722198 |
| H | 10.48122119168918 | 23.91379338341399 | 15.89318489670643 |
| H | 9.51055115048130 | 24.55464148900304 | 14.55370397397726 |
| H | 11.29482262625532 | 24.70553269410523 | 14.52139934007844 |
| C | 10.75805099395491 | 22.91739357091087 | 12.54928140542847 |
| H | 10.82960765741099 | 21.96092058443335 | 12.00006025597737 |
| H | 11.68914453194513 | 23.48439410131644 | 12.37053282546247 |
| H | 9.92904317975868 | 23.50314586154816 | 12.11509237495664 |
| C | 11.36256527838074 | 23.28758625541096 | 20.15620740420825 |
| C | 11.50318348251458 | 24.69834514676661 | 20.25482409862782 |
| C | 11.38374960933366 | 25.28774707485305 | 21.52627457497033 |
| H | 11.51485161261246 | 26.37081555582690 | 21.62751910528804 |
| C | 11.09213745680638 | 24.51679876554835 | 22.65956673332593 |
| H | 11.01354426990360 | 24.99446610494986 | 23.64406131325707 |
| C | 10.86203346592256 | 23.13930496787288 | 22.52637218232016 |
| H | 10.58993028246512 | 22.54883874934733 | 23.40989661224917 |
| C | 10.98345986657829 | 22.49880369286501 | 21.28057804143193 |
| C | 11.82022199797994 | 25.55958068712108 | 19.03774952116796 |
| H | 11.67682244624901 | 24.92580993645467 | 18.14223715581927 |
| C | 10.85517176526689 | 26.74877913692743 | 18.93012720210307 |
| H | 9.80950744817566 | 26.40612968541098 | 18.88111093213745 |
| H | 11.06702143123120 | 27.32909687287755 | 18.01647833508195 |
| H | 10.94376240338186 | 27.43450161930009 | 19.79059645159733 |
| C | 13.28080671587268 | 26.05323807706038 | 19.03559090866313 |
| H | 14.00025852906713 | 25.21750820898186 | 19.02413860136532 |
| H | 13.49065117766052 | 26.66701675159558 | 19.93160148996356 |
| H | 13.47463754825979 | 26.67765254728672 | 18.14415085012659 |
| C | 10.69999768371516 | 21.00623747898424 | 21.14318672198624 |
| H | 11.13628893543413 | 20.66929360219353 | 20.18541501072445 |
| C | 11.33644937362258 | 20.16595622153847 | 22.26505077179236 |
| H | 12.41897895964505 | 20.36399274017827 | 22.36250104386745 |
| H | 11.20245627696320 | 19.08850745282490 | 22.05915252996247 |
| H | 10.86853159217330 | 20.37230639555635 | 23.24516497036085 |
| C | 9.17831980038271 | 20.75939643327582 | 21.06326600881891 |
| H | 8.71756803492985 | 21.33582230826914 | 20.24381425563770 |
| H | 8.68748139753894 | 21.06330747097008 | 22.00675916010428 |
| H | 8.96651531421888 | 19.68679744439182 | 20.89736156709949 |

| | | | |
|---|---|---|---|
| H | -5.30993532149189 | 1.14329362919719 | 17.10969819278245 |
| C | -7.13722798248775 | 2.27116152281904 | 17.26087176105831 |
| H | -6.59811989928135 | 3.18614962230982 | 17.56598676011670 |
| H | -7.52378527705249 | 1.79517633940212 | 18.17787503366599 |
| H | -8.00589887104337 | 2.56874334414670 | 16.64524263693800 |
| C | -6.90725925160550 | -0.04815911835851 | 16.26734530435836 |
| H | -6.27667427589237 | -0.74379341013803 | 15.68681724787746 |
| H | -7.85848511765458 | 0.07677146237574 | 15.71797070641166 |
| H | -7.14467058911513 | -0.52645192589281 | 17.23467565564552 |
| C | -0.76418509137279 | 4.23121384739746 | 18.33820022762301 |
| C | -0.55774985455594 | 4.00131472231357 | 19.72448899210690 |
| C | -0.05941933014002 | 5.05812960640213 | 20.50908283833911 |
| H | 0.11969645694736 | 4.88979825645709 | 21.57859477740587 |
| C | 0.19849044909104 | 6.31894620563768 | 19.95470722404410 |
| H | 0.59338141707663 | 7.12788448305770 | 20.58172229523653 |
| C | -0.09315862895617 | 6.55072003668564 | 18.60274547318977 |
| H | 0.06240783439120 | 7.55022944164844 | 18.17789892069773 |
| C | -0.58429598346847 | 5.52308356323889 | 17.77752816161487 |
| C | -0.90591004841303 | 2.67443511192133 | 20.38463840155710 |
| H | -1.22325391536003 | 1.97514984134829 | 19.58977041597430 |
| C | -2.09982674816550 | 2.87644391304618 | 21.33500344775019 |
| H | -2.96900758169191 | 3.28683545062478 | 20.79384380234558 |
| H | -1.84488124946252 | 3.58460223882394 | 22.14439599020665 |
| H | -2.40761815759438 | 1.92587135195547 | 21.80090266808357 |
| C | 0.28845632184490 | 2.04043490731812 | 21.12179936145933 |
| H | 1.15396634690660 | 1.89328946515998 | 20.45149895146899 |
| H | 0.00551420787110 | 1.05578023472013 | 21.53662869249078 |
| H | 0.62315391380925 | 2.67206305121626 | 21.96545577625398 |
| C | -0.97035932895089 | 5.81566455010066 | 16.33618144739172 |
| H | -1.12798944700659 | 4.84533846700180 | 15.83173677987173 |
| C | -2.31266589049147 | 6.57064211099749 | 16.33755846249015 |
| H | -3.09669255598194 | 5.98513232415480 | 16.84914360854848 |
| H | -2.65674154508873 | 6.78593036020383 | 15.31115925138454 |
| H | -2.22288065218473 | 7.52895768925434 | 16.87788010721153 |
| C | 0.11637485017316 | 6.57480974670198 | 15.55642102613021 |
| H | 0.29517473346916 | 7.58193500928809 | 15.97592761724832 |
| H | -0.18927579705350 | 6.70828856748602 | 14.50281309606158 |
| H | 1.07782907858994 | 6.02977865798034 | 15.56666447260357 |

## O. Comparison of diketiminate bond metrics between 3 and $L^{tBu}CoCl$

For additional evidence that oxo complex **3** contains cobalt(II) ions, we compared the diketiminate bond metrics of the oxo (right ORTEP diagram) to the known three-coordinate cobalt(II) complex $L^{tBu}CoCl$ (left ORTEP diagram; from ref. 1 of ESI). All ligand backbone bonds are within error between the two compounds, arguing against ligand redox activity within the diketiminate supporting ligand.

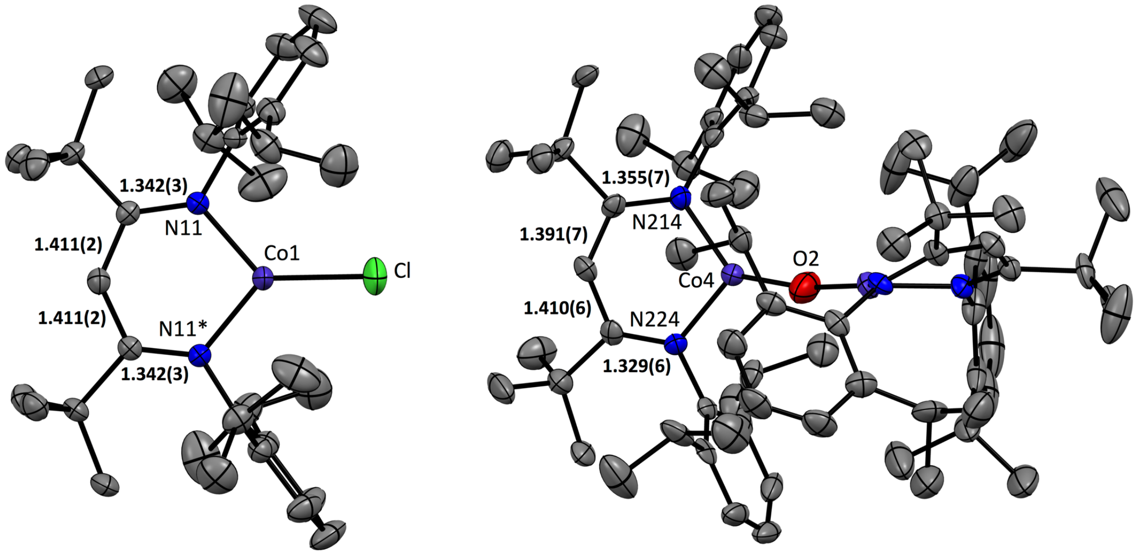

## P. Electronic structure of 3 from multi-configurational calculations.

**Table S5**. Configuration state functions with weight % for the first root calculation of complex **3**, obtained by **CASSCF(20,13)**. (2: doubly occupied MO, 0: unoccupied MO, 1: singly occupied MO with charge = 0, multiplicity = 1, E= -5297.8932652040 hartree)

| CSF | Weight % |
|---|---|
| 2221111121222 | 0.29 |
| 2221111212122 | 0.34 |
| 2221111221122 | 0.45 |
| 2221112212112 | 0.90 |
| 2221112221112 | 1.22 |
| 2221112222111 | 0.45 |
| 2221121121221 | 0.94 |
| 2221121221121 | 1.48 |
| 2221121221211 | 0.55 |
| 2221122121211 | 0.41 |
| 2221122212111 | 0.45 |
| 2221122221111 | 3.95 |
| 2221211112122 | 0.51 |
| 2221211112212 | 1.06 |
| 2221211121122 | 0.68 |
| 2221211121212 | 1.45 |
| 2221211122211 | 0.54 |
| 2221212112112 | 0.94 |
| 2221212121112 | 1.30 |
| 2221212122111 | 0.47 |
| 2221221112121 | 0.26 |
| 2221221112211 | 0.53 |
| 2221221121121 | 2.23 |

| CSF | Weight % |
|---|---|
| 2221221121211 | 4.65 |
| 2221221221111 | 0.50 |
| 2221222112111 | 0.47 |
| 2221222121111 | 4.15 |
| 2222111111222 | 0.45 |
| 2222111112221 | 1.24 |
| 2222111211122 | 0.70 |
| 2222111211212 | 0.26 |
| 2222111212121 | 1.98 |
| 2222111212211 | 0.75 |
| 2222111221121 | 0.32 |
| 2222112112211 | 0.55 |
| 2222112211112 | 1.89 |
| 2222112212111 | 5.24 |
| 2222112221111 | 0.84 |
| 2222121111221 | 1.15 |
| 2222121211121 | 1.85 |
| 2222121211211 | 0.68 |
| 2222122111211 | 0.51 |
| 2222122211111 | 4.87 |
| 2222211111122 | 1.08 |
| 2222211111212 | 2.23 |
| 2222211112121 | 2.97 |

| CSF | Weight % |
|---|---|
| 2222211112211 | 6.20 |
| 2222211121121 | 0.49 |
| 2222211121211 | 0.98 |
| 2222211212111 | 0.65 |
| 2222212111112 | 2.01 |
| 2222212112111 | 5.51 |
| 2222212121111 | 0.88 |
| 2222221111121 | 2.78 |
| 2222221111211 | 5.74 |
| 2222221211111 | 0.61 |
| 2222222111111 | 5.09 |

Table S5 shows that the first root of the CAS(20,13) calculation on intermediate 3 has several competing electronic configurations and has significant multiconfigurational character. However, all the CSFs reveal that the oxygen-based ligand orbitals, which are lowest in energy, are never singly occupied; they have predominantly oxygen $n_\pi$ orbital character. This analysis confirms that the oxygen center has all p-orbitals filled and is best described as $O^{2-}$, i.e, intermediate **3** has relatively polar bonds and is best described as $Co^{2+}$-$O^{2-}$-$Co^{2+}$.

**Figure S18.** Natural orbitals for CASSCF(24,17) calculation at S=0 with def2-TZVPP basis set. Atomic contributions and occupancy (in parenthesis) are also shown.

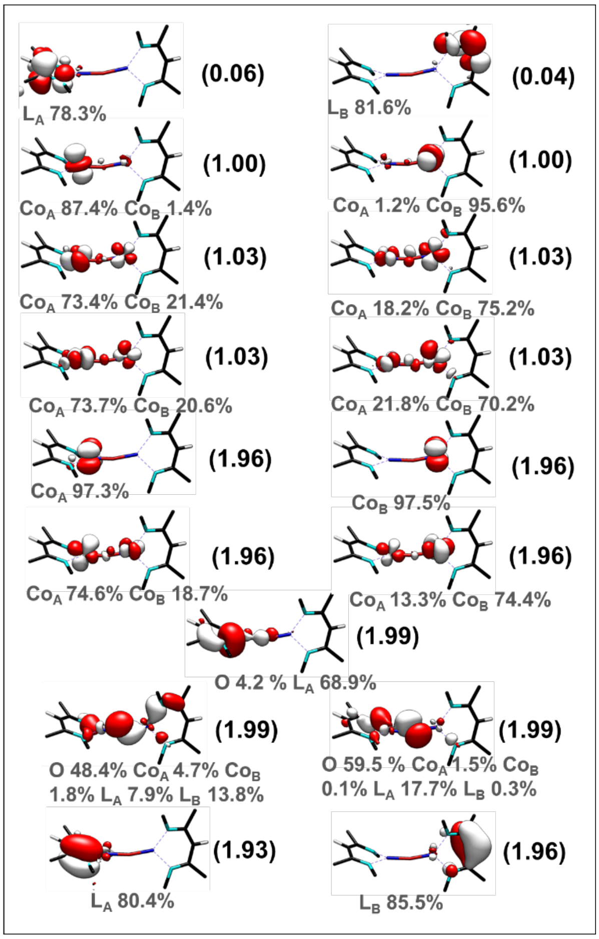


We constructed a larger active space considering the doubly-filled π and vacant π* orbitals of the β-diketiminate ligand in order to allow relaxation of the wave-function and explore excitation between these orbitals. The active space now consists of all metal based d-orbitals, the O centered p orbitals, and two high-lying π orbitals: one on each of the β-diketiminate ligands and their corresponding anti-bonding π* orbitals. This yielded an active space consisting of 24 electrons in 17 orbitals. In Figure S18, we have compared how the active space changes before and after calculation of CASSCF(24,17). Importantly, the π* orbitals of the β-diketiminate ligand were essentially vacant with occupation numbers in the range 0.04-0.06. Moreover, the π-orbitals of β-diketiminate ligand are not correlated to the Co-O-Co core (Figure S18) and the bonding between Co and the ligand N-C-N is primarily ionic in nature as evident from strong localization on the N-C-N motif (80-85%). Thus, inclusion of bonding and antibonding π orbitals of the β-diketiminate ligand does not change the overall description of the electronic structure of the cobalt centers as high-spin Co(II)**. This justifies the suitability of CASSCF(20,13) calculation for complex 3**.

## Q. Frontier orbital analysis during dinuclear associative pathway

**Figure S19.** Important orbitals during C-O cleavage mediated by dinuclear associative pathway.

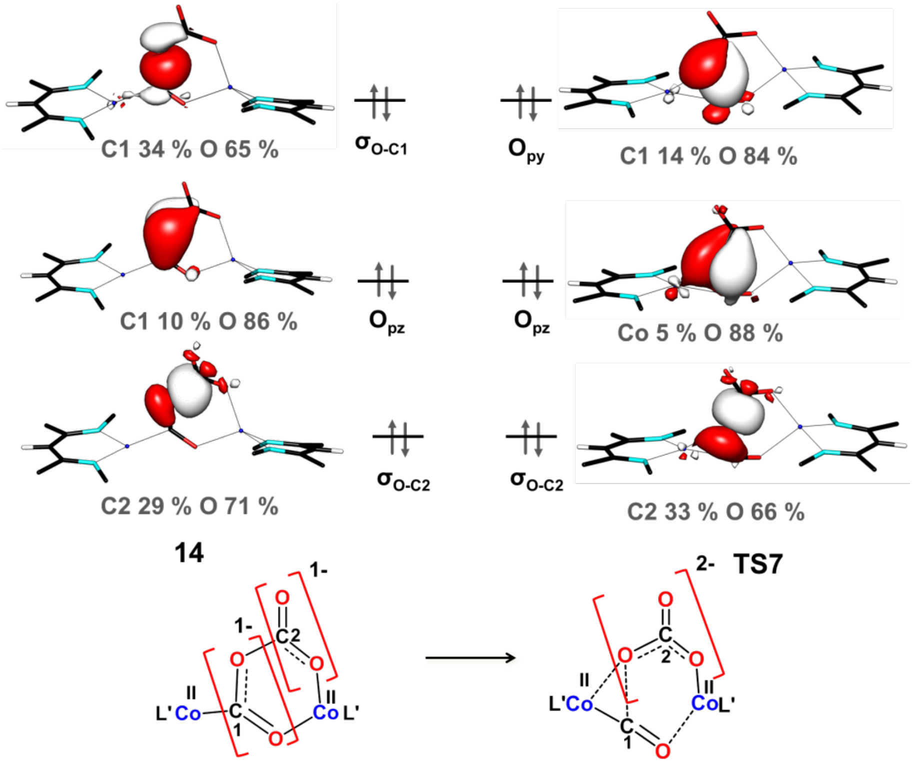


The dinuclear associative pathway involves heterolytic C-O bond cleavage via **TS7**. Here charge neutralization around the target oxygen center occurs due to interaction with the nearest metal center. The Lewis acidity of the metal center therefore plays a key role in facilitating the C-O bond scission. The Co-O interaction polarizes the target C-O bond, which may be responsible for the lower barrier pathway in the bimetallic species.

## R. Thermodynamics of additional pathways with mononuclear species

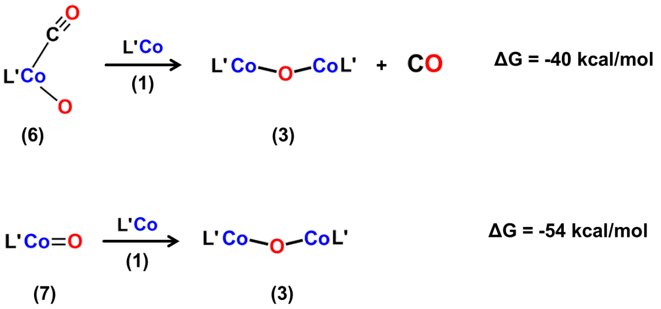


The formation of **3** from **6** or **7** in the presence of **1**, involving release of a CO molecule in **6** in the former reaction, was computed to have favorable driving forces; however, in the mononuclear dissociative pathway the generation of **6** and **7** has to transvers a high barrier for the C-O bond cleavage. Hence, these two pathways were not considered in the free energy profile in the main text.

## S. Why the mononuclear associative pathway is not predicted

$CO_2$ is a linear non-polar molecule featuring very short C-O bond lengths (1.16 Å). Due to the electronegativity difference between C and O, it has partially positive carbon and partially negative oxygen. Activation of $CO_2$ is manifested by bending of the O-C-O angle (~ 130 °) and a decrease in the C-O bond order. To be noted that $CO_2$ has a carbon-localized LUMO and the large HOMO-LUMO gap in linear molecule is not conducive for nucleophilic/electrophilic attack. Bending of the molecule lowers the energy of its LUMO, primarily of the in-plane orbital, and increases the carbon weight in its LUMO and oxygen content in its HOMO. These factors enhance the electron accepting ability of $CO_2$. Hence reduction of $CO_2$ requires a synchronous bi-functional activation.[36]

To the best of our knowledge, the mononuclear associative pathway has not been discussed in the literature in the context of a $(CO_2)_2$ dimer formation. Agarwal and others have proposed a mononuclear associative pathway consisting of association of **formic acid with $CO_2$**.[37] In that paper, the presence of a proton on HCOOH ensures strong hydrogen bonding with oxygen of incoming $CO_2$ which should have promoted overall electron transfer process in the bent $CO_2$. However, the dissociative pathway is a more common prediction and there are several examples of both mononuclear and dinuclear dissociative pathways studied theoretically by several groups with the β-diketiminate ligand based metal complexes of Fe, Co and Mn.[38-41] However, only a single study by Agarwal and others discusses the dinuclear associative pathway for insertion of $CO_2$ into a rhenium carboxylate dimer, $[Re(dmb)(CO)_3]_2$, where dmb = 4,4′-dimethyl-2,2′-bipyridine, to release CO and form a carbonate-bridged rhenium dimer.[42] Hence to say associative pathway is a unique mechanistic channel studied in the present report which has very few precedence both for mononuclear and dinuclear cases.

**Figure S20**. Various $CO_2$ activation pathways studied in this report: (a) $CO_2$ addition to the starting material, (b) $CO_2$ activation by cobalt-oxo species in the mononuclear dissociative pathway, (c) $CO_2$ activation by bridging $CO_2$ complex in intermediate **10** during dinuclear associative pathway, (d) $CO_2$ activation by the μ-oxo dicobalt complex **3** in the dinuclear dissociative pathway and (e) the computationally unobserved association of $CO_2$ to the $\eta^2$-(C,O) adduct in intermediate **5** to a hypothetical **5'**.

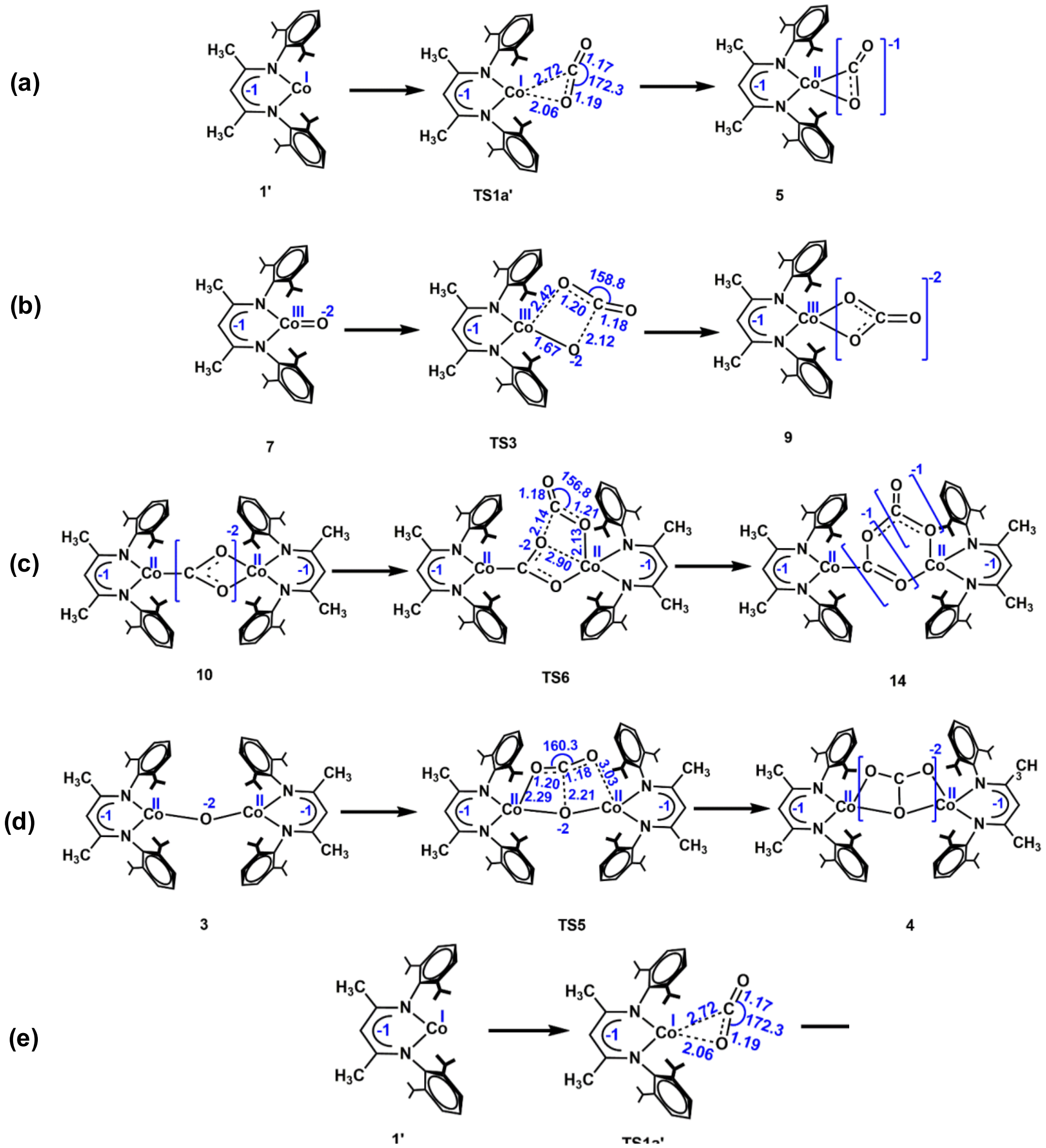


In the various $CO_2$ activation pathways that we have summarized above in Figure S20, we found that in each of the feasible pathways, $CO_2$ activation is manifested by cooperative effects of a Lewis acid (like a second metal center) and a Lewis base, electron-rich negatively charged oxo group or a two electron-reduced $[CO_2]^{2-}$. In intermediate **5**, there is only partial electron transfer from $Co^{I}$ to bound $CO_2$ which makes it $[CO_2]^{-1}$ in charge. Therefore, the nucleophilicity or basicity of this bound $CO_2$ is not conducive to promote electron transfer to another incoming $CO_2$. Moreover, the lack of a neighbouring Lewis acid group which might help in bending of the incoming $CO_2$ prior to activation is also a reason that inhibits facile association of a second $CO_2$ unit to the mononuclear adduct **5** and hence a mononuclear associative pathway is not possible. However, during dinuclear associative pathway, the bridging $CO_2$ which is actually $[CO_2]^{2-}$ has conducive basicity for nucleophilic attack to

the carbon of an incoming $CO_2$. Secondly, the second $CO_2$ could properly anchor on one of the cobalt centres, which decreases the O-C-O angle to 156 °, and enhances the possibility of electron acceptance through the carbon-based $CO_2$ LUMO. These two factors are responsible for a plausible dinuclear associative pathway.

## T. Total energies predicted at M06L(CPCM)/def-2TZVPP level of theory

| **Species** | **Energy in a.u.** |
|---|---|
| **1 (S = 1)** | -2639.75459 |
| **TS1 (S = 1)** | -2639.713401 |
| **1' (S = 1)** | -2639.746122 |
| **$CO_2$ (S = 0)** | -188.8549092 |
| **1a' (S = 1)** | -2828.612371 |
| **TS1a' (S = 1)** | -2828.609281 |
| **5 (S = 1)** | -2828.618511 |
| **TS2 (S=1)** | -2828.582323 |
| **6 (S = 1)** | -2828.591359 |
| **2 (S = 1)** | -2753.283541 |
| **7 (S = 1)** | -2715.090548 |
| **8 (S = 1)** | -2903.950627 |
| **TS3 (S = 1)** | -2903.938293 |
| **9 (S = 1)** | -2903.99207 |
| **TS2' (S = 2)** | -5467.278893 |
| **10 (S = 3)**<br>**(S = 0)** | -5468.453273<br>-5468.454382 |
| **TS4 (S = 3)**<br>**(S = 0)** | -5468.417772<br>-5468.420127 |

| | |
|---|---|
| **11 (S = 3)**<br>**(S = 0)** | -5468.448698<br>-5468.45327 |
| **3 (S = 3)**<br>**(S = 0)** | -5354.956605<br>-5354.964939 |
| **12 (S = 3)**<br>**(S = 0)** | -5543.822192<br>-5543.825493 |
| **TS5 (S = 3)**<br>**(S = 0)** | -5543.813262<br>-5543.817719 |
| **4 (S = 3)**<br>**(S = 0)** | -5543.847435<br>-5543.848663 |
| **13 (S = 3)**<br>**(S = 0)** | -5657.309157<br>-5657.309298 |
| **TS6 (S = 3)**<br>**(S = 0)** | -5657.286463<br>-5657.290172 |
| **14 (S = 3)**<br>**(S = 0)** | -5657.297302<br>-5657.29552 |
| **TS7 (S = 3)**<br>**(S = 0)** | -5657.284506<br>-5657.284082 |
| **15 (S = 3)**<br>**(S =0)** | -5657.314354<br>-5657.314219 |

## U. Electronic structure of Co-O-Co at different levels of Density Functional Theory.

**Figure S21.** Electronic structure of L′Co-O-CoL′ at (a) BP86 (S=3), (b) BP86 (S=0), (c) M06L (S=3), (d) M06L (S=0), (e) B3LYP (S=3) and (f) B3LYP (S=0) level of theory with the def2-TZVPP basis set.

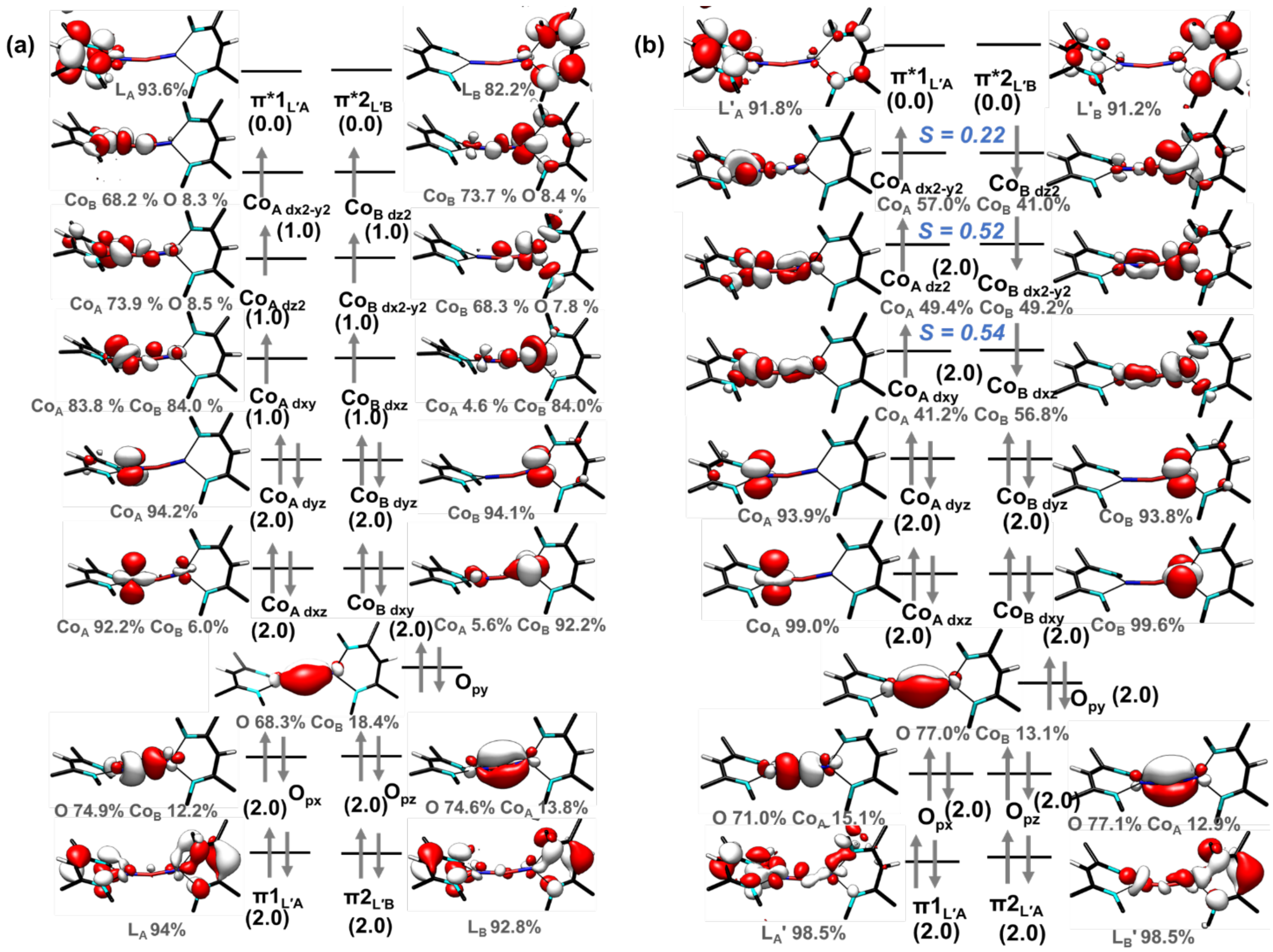

(c)

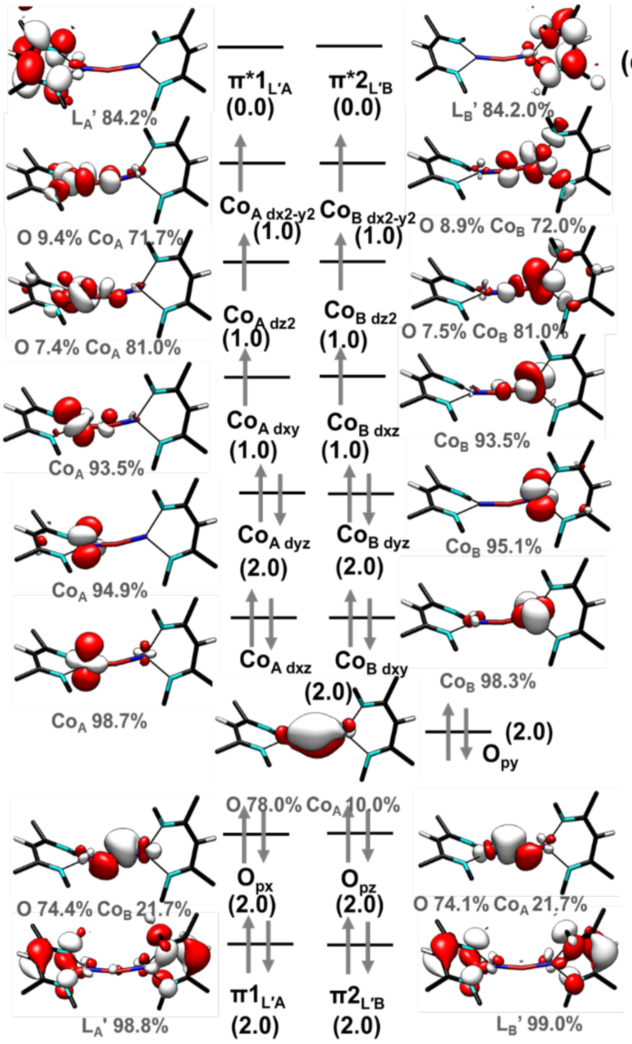

L_A' 84.2%
π*1 L'A (0.0)
π*2 L'B (0.0)
L_B' 84.2%
Co_A dx2-y2 (1.0)
Co_B dx2-y2 (1.0)
O 9.4% Co_A 71.7%
O 8.9% Co_B 72.0%
Co_A dz2 (1.0)
Co_B dz2 (1.0)
O 7.4% Co_A 81.0%
O 7.5% Co_B 81.0%
Co_A dxy (1.0)
Co_B dxz (1.0)
Co_A 93.5%
Co_B 93.5%
Co_A dyz (2.0)
Co_B dyz (2.0)
Co_A 94.9%
Co_B 95.1%
Co_A dxz
Co_B dxy
Co_A 98.7%
Co_B 98.3%
(2.0)
O_py (2.0)
O 78.0% Co_A 10.0%
O_px (2.0)
O_pz (2.0)
O 74.4% Co_B 21.7%
O 74.1% Co_A 21.7%
π1 L'A (2.0)
π2 L'B (2.0)
L_A' 98.8%
L_B' 99.0%


(d)

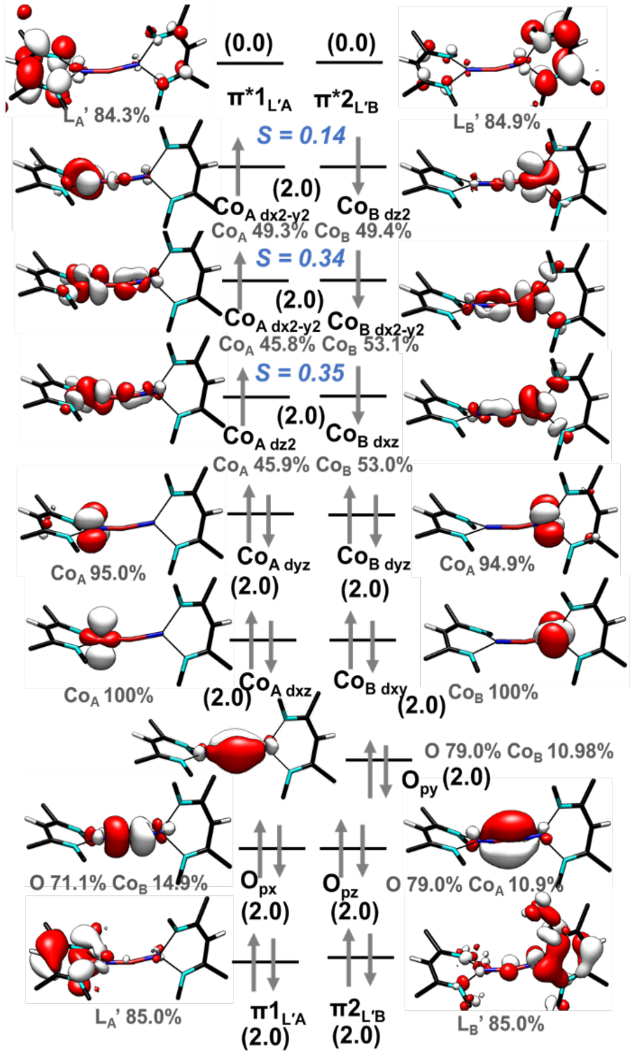

(0.0)
(0.0)
π*1 L'A
π*2 L'B
L_A' 84.3%
L_B' 84.9%
S = 0.14
Co_A dx2-y2
(2.0)
Co_B dz2
Co_A 49.3% Co_B 49.4%
S = 0.34
(2.0)
Co_A dx2-y2
Co_B dx2-y2
Co_A 45.8% Co_B 53.1%
S = 0.35
(2.0)
Co_A dz2
Co_B dxz
Co_A 45.9% Co_B 53.0%
Co_A dyz
Co_B dyz
(2.0)
(2.0)
Co_A 95.0%
Co_A 94.9%
Co_A dxz
Co_B dxy
(2.0)
(2.0)
Co_A 100%
Co_B 100%
O 79.0% Co_B 10.98%
O_py (2.0)
O_px (2.0)
O_pz (2.0)
O 71.1% Co_B 14.9%
O 79.0% Co_A 10.9%
π1 L'A (2.0)
π2 L'B (2.0)
L_A' 85.0%
L_B' 85.0%

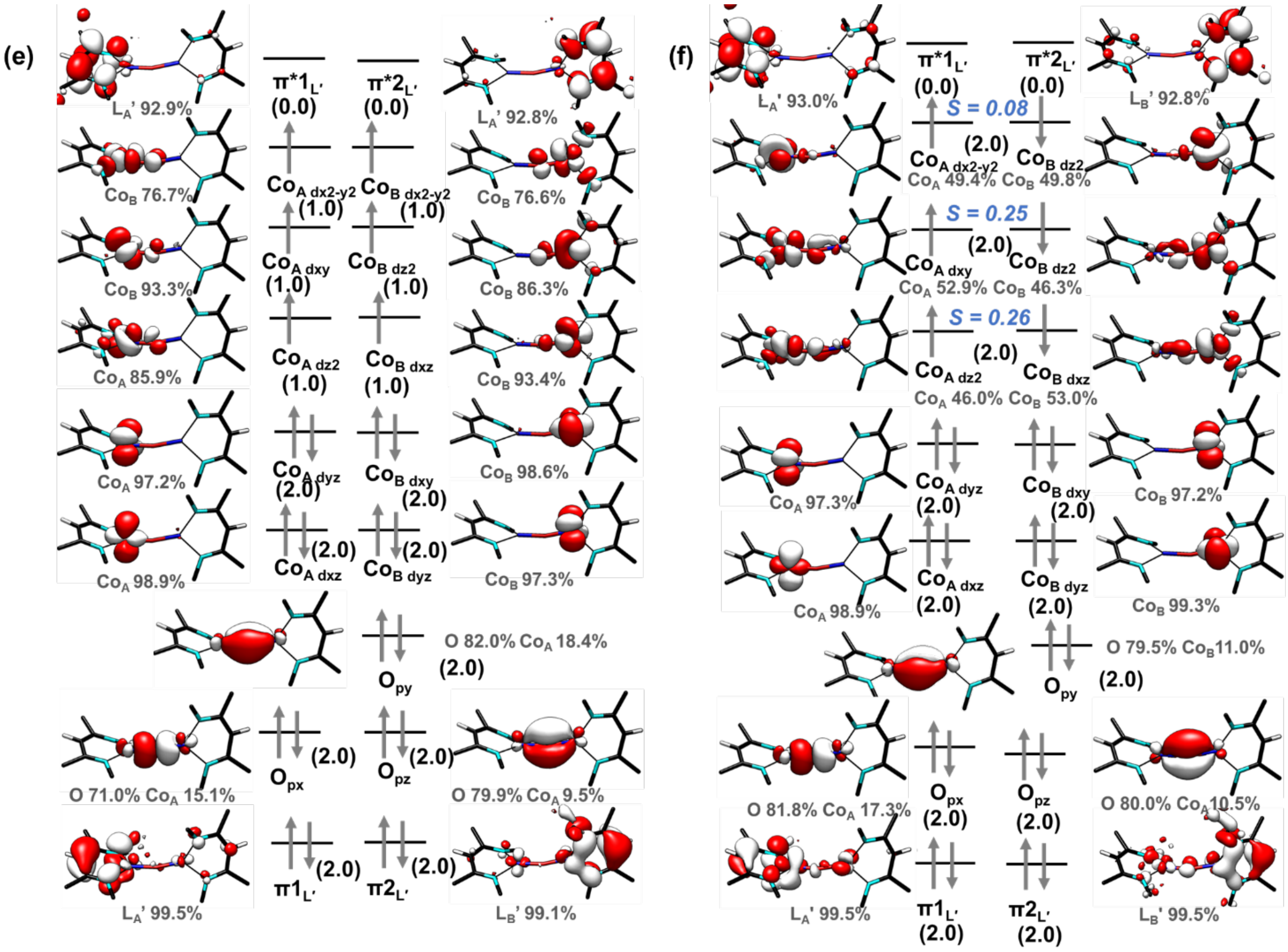


The choice of functional can have important influences on the result of a computational study, and therefore we used several different density functionals: BP86 (which is a local gradient corrected functional consisting of Becke's exchange and Perdew's correlation), M06L (the local meta-GGA functional developed by Truhlar and co-workers) and the most popular hybrid functional, B3LYP (consisting of 20% Hartree Fock exchange) to evaluate the electronic structure of intermediate **3**. High-spin models using **each of these functionals** gave quasi-restricted DFT orbitals consistent with a $d^7$ configuration at cobalt, with 4 electrons in the doubly-occupied region and 3 electrons in the singly occupied region. The doubly occupied orbitals have > 90% Co d-parentage while the singly occupied orbitals have ~ 70-90% contribution from metal-based d-orbitals. This is also true for the broken-symmetry models, where we also found three anti-ferromagnetic coupling (for e.g. small overlap in the range of 0.5 to 0.2 at BP86/def-TZVPP level) between the non-orthogonal orbitals of either metal site, in addition to four uncoupled doubly occupied orbitals. **Therefore, analysis of electron occupancy with different HF exchange indicate unanimously the dicobalt(II) oxo electronic structure**. The schematic representation of the electronic structure of species **3** (both with S=0 and S=3) along with occupancy and atomic contributions to each orbitals with these three different functionals containing different % HF exchange is provided above in Figure S21.

**Figure S22.** Molecular electrostatic potential (MEP) surface of intermediate **3** at BP86/def2-TZVPP. Value of isosurface = 0.02 au. The color spectrum ranges from negative (blue) to positive (red) electrostatic potential within ±1.0e-2 a.u.

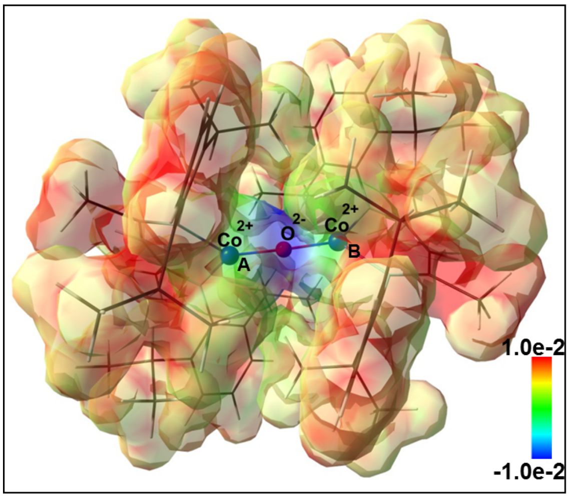


The MEP plot in Figure S22 reveals distribution of negative electrostatic potential (blue colored isosurface) centrally on oxygen and positive electrostatic potential (red colored isosurface) on some parts of the ligand. The cobalt centers are in the borderline region (green colored isosurface). The MEP mapping clearly shows that the oxygen in species **3** is enveloped by a negative electronic potential and explains the reason for its high reactivity.

**Table S6**. Mulliken gross atomic charges on intermediate **3** with different density functionals employing the def2-TZVPP basis set.

**S = 3**

| **Atom** | **BP86** | **B3LYP** | **M06L** |
|---|---|---|---|
| **$Co_A$** | 0.6380 | 0.7852 | 0.6762 |
| **O** | -0.6812 | -0.8504 | -0.6881 |
| **$Co_B$** | 0.6411 | 0.7829 | 0.6857 |

**S = 0**

| **Atom** | **BP86** | **B3LYP** | **M06L** |
|---|---|---|---|
| **$Co_A$** | 0.6036 | 0.7706 | 0.6593 |
| **O** | -0.6195 | -0.8169 | -0.6645 |
| **$Co_B$** | 0.5989 | 0.7689 | 0.6503 |

## V. References for ESI


1. K. Ding, P. L. Holland, D. Adhikari and D. J. Mindiola, *Inorg. Synth.*, 2010, 35, 43-45.
2. T. R. Dugan, X. Sun, E. V. Rybak-Akimova, O. Olatunji-Ojo, T. R. Cundari and P. L. Holland, *J. Am. Chem. Soc.*, 2011, 133, 12418-12421.
3. M. H. Al-Afyouni, E. Suturina, S. Pathak, M. Atanasov, E. Bill, D. E. DeRosha, W. W. Brennessel, F. Neese and P. L. Holland, *J. Am. Chem. Soc.*, 2015, 137, 10689-10699.
4. A. D. Becke, *Phys. Rev. A*, 1988, 38, 3098-3100.
5. J. P. Perdew, *Phys. Rev. B*, 1986, 33, 8822-8824.
6. A. Schäfer, C. Huber and R. Ahlrichs, *J. Chem. Phys.*, 1994, 100, 5829-5835.
7. A. Schäfer, H. Horn and R. Ahlrichs, *J. Chem. Phys.*, 1992, 97, 2571-2577.
8. B. Kirchner, F. Wennmohs, S. Ye and F. Neese, *Curr. Opin. Chem. Biol.*, 2007, 11, 134-141.
9. L. Noodleman, *J. Chem. Phys.*, 1981, 74, 5737-5743.
10. F. Neese, *WIREs Comput. Mol. Sci.*, 2012, 2, 73-78.
11. F. Neese, *WIREs Comput. Mol. Sci.*, 2018, 8:e1327.
12. K. Eichkorn, Weigend, F., Treutler, O. et al., *Theor. Chem. Acta.*, 1997, 97, 119-124.
13. K. Eichkorn, O. Treutler, H. Öhm, M. Häser and R. Ahlrichs, *Chem. Phys. Lett.*, 1995, 242, 652-660.
14. K. Eichkorn, O. Treutler, H. Öhm, M. Häser and R. Ahlrichs, *Chem. Phys. Lett.*, 1995, 240, 283-290.
15. E. v. Lenthe, E. J. Baerends and J. G. Snijders, *J. Chem. Phys.*, 1993, 99, 4597-4610.
16. E. van Lenthe, E. J. Baerends and J. G. Snijders, *J. Chem. Phys.*, 1994, 101, 9783-9792.
17. E. van Lenthe, R. van Leeuwen, E. J. Baerends and J. G. Snijders, *Int. J. Quantum Chem.*, 1996, 57, 281-293.
18. D. A. Pantazis, X.-Y. Chen, C. R. Landis and F. Neese, *J. Chem. Theory Comput.*, 2008, 4, 908-919.
19. J. Tomasi, B. Mennucci and R. Cammi, *Chem. Rev.*, 2005, 105, 2999-3094.
20. C. Lee, W. Yang and R. G. Parr, *Phys. Rev. B*, 1988, 37, 785-789.
21. Y. Zhao and D. G. Truhlar, *J. Chem. Phys.*, 2006, 125, 194101.
22. F. Weigend and R. Ahlrichs, *Phys. Chem. Chem. Phys.*, 2005, 7, 3297-3305.
23. F. Neese, F. Wennmohs, A. Hansen and U. Becker, *Chem. Phys.*, 2009, 356, 98-109.
24. S. Grimme, S. Ehrlich and L. Goerigk, *J. Comput. Chem.*, 2011, 32, 1456-1465.
25. S. Grimme, J. Antony, S. Ehrlich and H. Krieg, *J. Chem. Phys.*, 2010, 132, 154104.
26. M. Saitow, U. Becker, C. Riplinger, E. F. Valeev and F. Neese, *J. Chem. Phys.*, 2017, 146, 164105.
27. C. Riplinger and F. Neese, *J. Chem. Phys.*, 2013, 138, 034106.
28. B. Mondal, F. Neese and S. Ye, *Inorg. Chem.*, 2015, 54, 7192-7198.
29. J. Song, E. L. Klein, F. Neese and S. Ye, *Inorg. Chem.*, 2014, 53, 7500-7507.
30. K. Ruedenberg, L. M. Cheung and S. T. Elbert, *Int. J. Quantum Chem.*, 1979, 16, 1069-1101.
31. B. O. Roos, P. R. Taylor and P. E. M. Siegbahn, *Chem. Phys.*, 1980, 48, 157-173.
32. C. Angeli, R. Cimiraglia, S. Evangelisti, T. Leininger and J. P. Malrieu, *J. Chem. Phys.*, 2001, 114, 10252-10264.
33. K. D. Finkelstein, C. J. Pollock, A. Lyndaker, T. Krawcyk and J. Conrad, *AIP Conf. Proc.*, 2016, 1741, 030009.
34. R. C. Walroth, J. T. Lukens, S. N. MacMillan, K. D. Finkelstein and K. M. Lancaster, *J. Am. Chem. Soc.*, 2016, 138, 1922-1931.
35. D. E. DeRosha, B. Q. Mercado, G. Lukat-Rodgers, K. R. Rodgers and P. L. Holland, *Angew. Chem. Int. Ed.*, 2017, 56, 3211-3215.
36. B. Mondal, J. Song, F. Neese and S. Ye, *Curr. Opin. Chem. Biol.*, 2015, 25, 103-109.
37. J. Agarwal, B. C. Sanders, E. Fujita, H. F. Schaefer Iii, T. C. Harrop and J. T. Muckerman, *Chem. Commun.*, 2012, 48, 6797-6799.

38. A. Ariafard, N. J. Brookes, R. Stranger, P. D. W. Boyd and B. F. Yates, *Inorg. Chem.*, 2010, 49, 7773-7782.
39. C. Liu, L. Munjanja, T. R. Cundari and A. K. Wilson, *J. Phys. Chem. A*, 2010, 114, 6207-6216.
40. C. Liu, T. R. Cundari and A. K. Wilson, *Inorg. Chem.*, 2011, 50, 8782-8789.
41. V. Canale, R. Robinson, A. Zavras, G. N. Khairallah, N. d'Alessandro, B. F. Yates and R. A. J. O'Hair, *J. Phys. Chem. Lett.*, 2016, 7, 1934-1938.
42. J. Agarwal, E. Fujita, H. F. Schaefer and J. T. Muckerman, *J. Am. Chem. Soc.*, 2012, 134, 5180-5186.
